# Persistent burden and health inequalities of disease in women of childbearing age attributable to Intimate Partner Violence, 1990–2021

**DOI:** 10.3389/fpsyt.2025.1515828

**Published:** 2025-08-20

**Authors:** Yingda Song, Juan Wang, Jiaxuan Wang, Yan Ren, Jun Ma

**Affiliations:** ^1^ Cardiac Center Department, Shanxi Provincial People's Hospital, Taiyuan, Shanxi, China; ^2^ Thoracic Surgery Department, Shanxi Provincial People's Hospital, Taiyuan, Shanxi, China; ^3^ Fifth Clinical Medical College, Shanxi Medical University, Taiyuan, Shanxi, China; ^4^ Psychiatry Department, Shanxi Provincial People's Hospital, Taiyuan, Shanxi, China; ^5^ First Clinical Medical College, Changzhi Medical College, Changzhi, Shanxi, China

**Keywords:** Intimate Partner Violence, epidemiology, women of childbearing age, COVID-19, Global Burden of Disease 2021

## Abstract

**Aims:**

Intimate Partner Violence (IPV) presents a significant global public health issue, particularly affecting women of childbearing age (WBCA). The COVID-19 pandemic has exacerbated the IPV globally. This study aimed to assess the global burden and health inequalities attributable to IPV among WBCA from 1990 to 2021.

**Methods:**

We utilized data from the Global Burden of Disease (GBD) 2021 to assess the disease burden attributable to IPV among WBCA, by age group, GBD region, and country, measured using mortality, disability-adjusted life years (DALYs), age-standardized mortality rate (ASMR), and age-standardized DALY rate (ASDR). Joinpoint regression was utilized to examine trends over time. To assess both absolute and relative health disparities, the Slope Index of Inequality (SII) and the Concentration Index were computed.

**Results:**

In 2021, global IPV-related mortality and DALYs among WBCA were 44,661 and 5.35 million, respectively. The IPV-attributable ASDR declined from 292.88 per 100,000 population in 1990 (95% uncertainty interval [UI]: 167.98 to 439.65) to 272.08 per 100,000 population in 2021 (95% UI: 148.65 to 422.82), with average annual percentage change (AAPC) of -0.19 (95% confidence interval [CI]: -0.39 to -0.10). Significant inflection points were identified in the years 2000 and 2019. In 2019, the IPV-attributable ASDR began to rise. This upward trend was primarily driven by IPV-related depressive disorders burden. The IPV-attributable ASMR decreased from 2.61 per 100,000 population in 1990 (95% UI: 1.73 to 3.71) to 2.26 per 100,000 population in 2021 (95% UI: 1.45 to 3.21), with AAPC of -0.46 (95% CI: -0.60 to -0.33). This downward trend was also observed in the burden caused by IPV-related HIV/AIDS and interpersonal violence. In 2021, the highest disease burden attributable to IPV was reported among the 30–34 age group, low and low-middle Socio-Demographic Index (SDI) regions, and Eastern Sub-Saharan Africa. The largest increase in disease burden attributable to IPV occurred in the 40–44 age group, Oceania, and low-middle SDI region. The absolute value of the SII for ASMR increased from 2.04 in 1990 to 4.59 in 2021, while that of the SII for ASDR decreased from 321 in 1990 to 190 in 2021. The relative Concentration Index for ASMR and ASDR dropped from -0.33 and -0.2 in 1990 to -0.46 and -0.26 in 2021, respectively. The worsening of health inequalities was mainly concentrated in low- and middle-income countries (LMICs) and IPV-related HIV/AIDS burden.

**Conclusions:**

Since 1990, the burden attributable to IPV among WBCA has generally declined. However, the COVID-19 pandemic reversed this trend, particularly in IPV-related depressive disorders burden. Health inequalities have worsened, particularly in relation to IPV-related HIV/AIDS burden. Increased attention must be given to eliminating the preventable burden of IPV in LMICs, with coordinated global efforts required to mitigate its health impact.

## 1 Introduction

Intimate Partner Violence (IPV) represents a major concern in global public health, posing a particularly severe threat to women (1). IPV encompasses multiple forms of abuse, including physical, sexual, psychological, and economic, that commonly occur within the context of current or former intimate relationships (2). Extensive research has demonstrated that IPV not only profoundly affects women’s physical health but also contributes to a broad spectrum of mental health disorders and adverse social outcomes. According to the World Health Organization (WHO), approximately 30% of women globally will experience some form of IPV during their lifetime, with women of childbearing age (WBCA) being particularly vulnerable (3).

The disease burden attributable to IPV among WBCA has been recognized as multifaceted. First, IPV has been linked to multiple severe health issues, including sexually transmitted infections, adverse pregnancy outcomes, and chronic diseases (4). Second, IPV significantly impacts women’s mental health, with studies indicating a strong correlation between IPV and mental illnesses such as depression, anxiety, and post-traumatic stress disorder (PTSD) (5). Such health issues not only impact women directly but also have profound negative effects on their children, families, and society as a whole. Given that WBCA represent a critical population for reproductive health and family planning, effective interventions at this stage are crucial for improving public health outcomes.

Globally, interventions and prevention strategies for IPV have made certain progress, but significant regional differences persist. In North America and Europe, there has been long-term attention to developing comprehensive plans, including providing legal, social, and economic support for abused women through shelters and hotlines. In the healthcare sector, significant progress has been made, such as implementing screening as a secondary prevention strategy for identifying and referring female IPV victims, particularly routine screening and home visits during pregnancy (6–9). In contrast, research and prevention of IPV remains limited in most low- and middle-income countries, where existing evidence demonstrates typically higher IPV prevalence rates (10–12).

The COVID-19 pandemic has been documented to further exacerbate these disparties. The COVID-19 pandemic has resulted in increased social isolation, economic pressure, and psychological stress, with many countries reporting a significant rise in IPV-related cases. Quarantine measures and lockdown policies compelled many women to remain with their abusers for prolonged periods, while opportunities to seek external help decreased, leading to a substantial increase in IPV exposure (13). Moreover, COVID-19 not only increased the frequency of IPV but also hindered IPV victims’ access to healthcare services. As healthcare resources were massively redirected to combat the pandemic, many IPV-related medical services were delayed or canceled, thereby limiting victims’ ability to receive timely medical assistance. Studies have shown that during the pandemic, mental health issues related to IPV also increased, with victims reporting heightened anxiety and depression symptoms (14).

The impact of COVID-19 on the burden attributable to IPV has been particularly pronounced in low- and middle-income countries (LMICs). In these regions, women were already facing elevated risks of IPV, and the pandemic exacerbated this due to limited healthcare resources, inadequate legal protections, and increasing socio-economic pressures (15). As a result, the IPV burden in these countries has worsened considerably. For instance, a study conducted in India reported a more than 50% increase in domestic violence cases during the pandemic, with rural areas experiencing the most significant surge (16).

However, despite existing research documenting the global distribution and influencing factors of IPV, with some studies beginning to explore the impact of the COVID-19 pandemic on IPV, several critical research gaps remain: (1) lack of comprehensive assessment of the burden of various IPV-related diseases on women of childbearing age, a high-risk population; (2) limited analysis of health inequalities in the disease burden attributable to IPV; (3) regional differences in the impact of the COVID-19 pandemic on IPV burden have not been adequately quantified; (4) insufficient research on the effectiveness of IPV interventions across different socioeconomic contexts.

This study aims to address these gaps by utilizing Global Burden of Disease (GBD) 2021 data to provide the first comprehensive global research perspective on IPV-related disease burden among women of childbearing age, enabling us to evaluate patterns of change in global IPV burden before and after the pandemic, identify the most vulnerable populations and regions, and provide evidence for developing more effective and targeted global and regional IPV prevention and intervention strategies. Therefore, our main research objectives include: (1) to investigate the patterns and trends of the disease burden attributable to IPV among WBCA, and (2) to assess the trends in health inequalities related to the IPV-attributable disease burden from 1990 to 2021.

## 2 Methods

### 2.1 Overview

This study is based on the GBD 2021 study, which is currently the most comprehensive disease burden estimation project worldwide, led by the Institute for Health Metrics and Evaluation at the University of Washington. The GBD data collection integrates information from multiple sources, including epidemiological surveys, hospital records, vital registration systems, disease surveillance networks, as well as academic literature and policy reports. Regarding methodological framework, the GBD 2021 database employed the Bayesian, regularized, and trimmed meta-regression to quantify the effect size of each risk-outcome pair. Nonlinear variations in relative risk (RR) functions were captured through integrated spline functions and adjusted for systematic bias. RR estimates were derived from data obtained through systematic reviews and meta-analyses, with inclusion criteria determined by the 95% uncertainty interval (UI). Additionally, spatio-temporal Gaussian process regression and disease model meta-regression 2.1 were applied to aggregate heterogeneous data and correct for biases, thus estimating the exposure levels and distributions for each specific risk factor. The described methods of the GBD 2021 study and the risk assessment involving the disease burden attributable to IPV were comprehensively detailed in the collaborator group’s article (17). This study extracts and analyzes relevant data based on these established methods to assess the impact of IPV on women’s health globally.

In this study, estimates and the 95% UI for mortality and disability-adjusted life years (DALYs) disease burden attributable to IPV were extracted. The dataset from GBD 2021 encompasses global levels, five Socio-Demographic Index (SDI) level regions, 21 GBD regions, and 204 countries and territories from 1990 to 2021. Notably, all attribution estimates are based on the best available evidence from systematic reviews and meta-analyses, with inclusion criteria strictly determined by the 95% UI, ensuring the scientific validity and reliability of the analysis results.

### 2.2 Data sources

In the GBD 2021 study, IPV is defined as the experience of one or more instances of physical and/or sexual violence perpetrated by a current or former intimate partner from the age of 15 onward. An intimate partner includes a spouse, cohabiting partner, or, in regions where dating is common, a dating partner with whom one has an intimate or sexual relationship. Physical violence includes actions such as slapping, pushing, hitting, kicking, choking, or threats/attacks with a weapon. Sexual violence refers to being forced into intercourse, engaging in sexual acts out of fear, or being coerced into humiliating or degrading acts, with definitions varying by regional and cultural context. The WBCA group includes individuals aged between 15 and 49 years (18). The 371 diseases and injuries in GBD 2021 are organized into hierarchical structure with four levels. In GBD 2021, three diseases and injuries of level 3 disease groups were judged to meet scientific evidence of causal relationships attributable to IPV, including HIV/AIDS, interpersonal violence, and depressive disorders, where depressive disorders are non-fatal outcomes, while HIV/AIDS and interpersonal violence are fatal outcomes. Therefore, depressive disorders are measured only by DALYs, while HIV/AIDS and interpersonal violence are assessed using both DALYs and mortality indicators.

HIV/AIDS is classified in the International Classification of Diseases 10 (ICD-10) by B20-B23.8, B24-B24.0, B97.81, C46-C46.52, C46.7- C46.9, F02.4, O98.7-O98.73, Z11.4, Z20.6-Z21, and Z83.0. Depressive disorder is classified in the ICD-10 by F32-F33.9, and F34.1. Interpersonal violence is classified in the ICD-10 by T74.2-T76.22, X85-Y08.9, and Y87.1-Y87.2 (19). The SDI, developed by GBD researchers, is a composite measure used to assess socio-economic conditions affecting health outcomes across regions. In the GBD 2021 study, SDI values range from 0 to 1, with higher values indicating better socio-economic conditions and improved health outcomes. Based on 2021 SDI values, regions are divided into five quintiles: low (0-0.466), low-middle (0.466-0.619), middle (0.619-0.712), high-middle (0.712-0.810), and high (0.810-1) (19).

### 2.3 Statistics analysis

The estimates were age-standardized using direct age-standardization. The purpose of age-standardization is to eliminate the effect of differences in population age structure (20, 21).

The joinpoint regression model was used to analyze the temporal trends of the age specific rate and age-standardized rate (ASR) from 1990 to 2021. The trend was quantified using the annual percentage change (APC) and the average annual percentage change (AAPC). APC is an indicator used to describe the year-to-year change, while AAPC is an indicator used to describe the average changes over a longer period of time. The ASRs and absolute numbers for mortality and DALYs were extracted for inequality analysis. According to the World Health Organization Health Equity Assessment guidelines, the Slope Index of Inequality (SII) and Concentration Index serve as two fundamental measures for evaluating absolute and relative income-related inequalities, which are used to assess health inequalities of disease burden across countries. The SII reflects the absolute difference in the above indicators between the lowest-SDI and highest-SDI countries, with higher absolute values of SII indicating greater inequality. The Concentration Index was calculated using the Lorenz curve based on per capita SDI and the corresponding national burden metrics. It reflects the area between the 45° line and the Lorenz curve. A negative index indicates that the burden is higher in low-income countries. Conversely, a positive index indicates a higher burden in high-income countries.

ASR was reported per 100,000 population, with data presented as values with 95% UI. Temporal trends were assessed using joinpoint software (version 5.0.2) from the National Cancer Institute. The values of the AAPC and APC are expressed as percentages, with data presented as values accompanied by 95% confidence intervals (CI). All statistical analyses and mapping were performed using R statistical software (version 4.3.3). A two-sided *P* value < 0.05 was set as the significance threshold.

## 3 Results

### 3.1 Global burden trends

**Figure 1:**
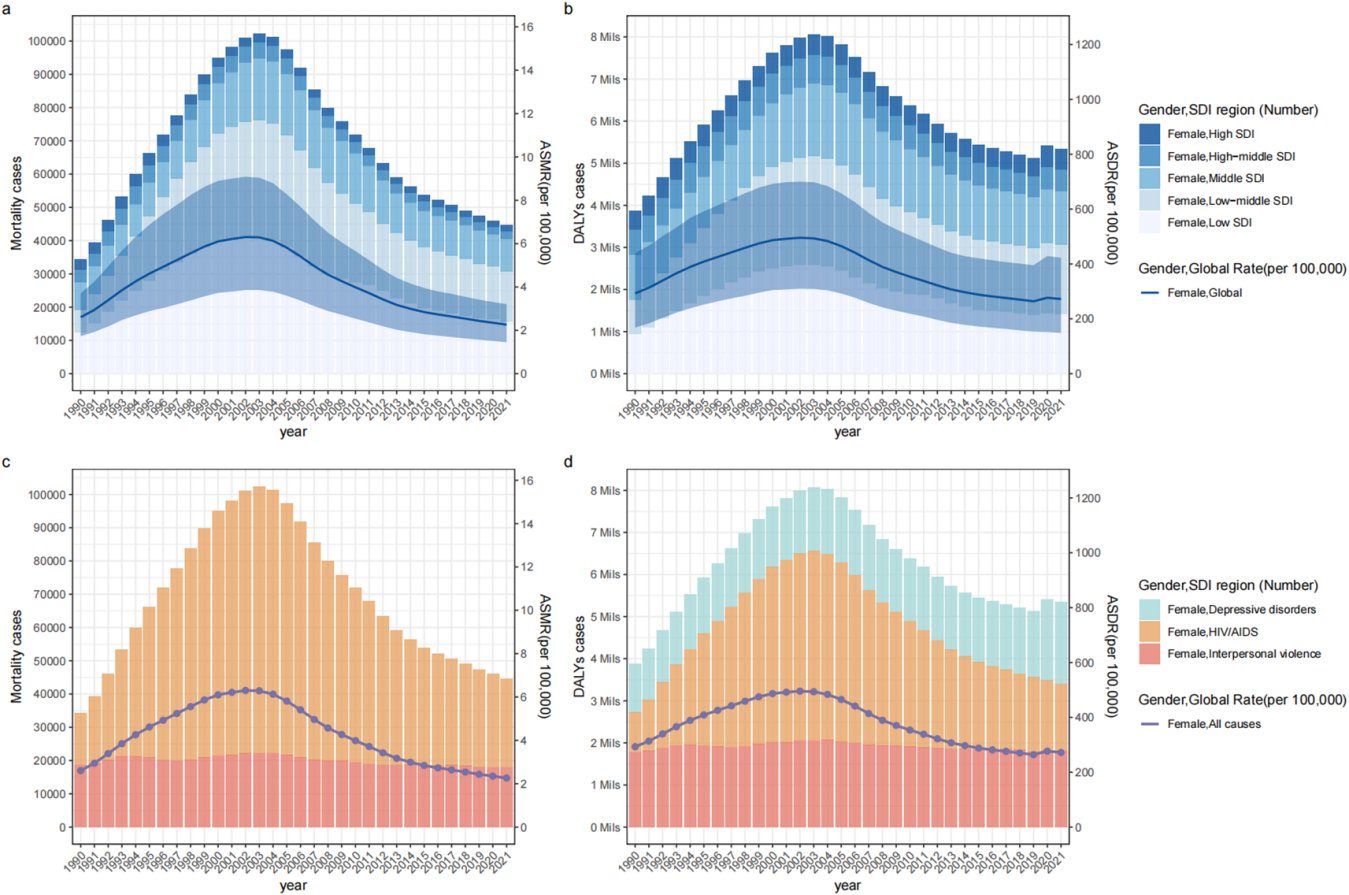
The disease burden of mortality **(a, c)** and DALYs **(b, d)** attributable to IPV among women of childbearing age from 1990 to 2021 by Cause **(c, d)** or SDI region **(a, b)**. DALYs, disability-adjusted life-years; SDI, sociodemographic index; ASMR, age-standardized mortality rate; ASDR, age-standardized DALYs rate; IPV, Intimate Partner Violence.

Globally, the number of IPV-attributable mortality and DALYs among WBCA in 2021 were 44,661 (95% UI: 28,636 to 63,421) and 5,348,948 (95% UI: 2,915,444 to 8,317,919), respectively. The IPV-attributable ASDR among WBCA in 2021 (272.08 cases per 100,000 population, 95% UI: 148.65 to 422.82) was lower compared to 1990 (292.88 cases per 100,000 population, 95% UI: 167.98 to 439.65; AAPC = -0.19, 95% CI: -0.39 to -0.10, *P* = 0.003); the ASMR among WBCA in 2021 (2.26 cases per 100,000 population, 95% UI: 1.45 to 3.21) was lower compared to 1990 (2.61 cases per 100,000 population, 95% UI: 1.73 to 3.71; AAPC = -0.46, 95% CI: -0.60 to -0.33, P < 0.001) (**Table 1**, **Figures 1a, b**). Compared to 1990, these rates have significantly decreased, while the number of cases has markedly increased.

**Table 1:** Mortality and DALYs of disease burden attributable to IPV among women of childbearing age in 1990 and 2021, and AAPC from 1990 to 2021, by SDI quintile and region level.

Location	Mortality					DALYs				
	Mortality cases in 1990 (95% UI)	ASMR in 1990 (95% UI)	Mortality cases in 2021 (95% UI)	ASMR in 2021 (95% UI)	AAPC% (95%CI), 1990–2021	DALYs cases in 1990 (95% UI)	ASDR in 1990 (95% UI)	DALYs cases in 2021 (95% UI)	ASDR in 2021 (95% UI)	AAPC% (95%CI), 1990–2021
Global	34372 (22722 to 48826)	2.61 (1.73 to 3.71)	44661 (28636 to 63421)	2.26 (1.45 to 3.21)	-0.46 (-0.60 to -0.33)	3885331 (2248276 to 5817775)	292.88 (167.98 to 439.65)	5348948 (2915444 to 8317919)	272.08 (148.65 to 422.82)	-0.19 (-0.40 to 0.02)
Age										
15–19 years	3498 (2188 to 5000)	1.37 (0.86 to 1.96)	3409 (2195 to 4756)	1.12 (0.72 to 1.57)	-0.65 (-0.94 to -0.36)	425415 (269159 to 624653)	166.48 (105.33 to 244.45)	447453 (278546 to 664743)	147.36 (91.73 to 218.92)	-0.36 (-0.53 to -0.20)
20–24 years	5620 (3527 to 7916)	2.30 (1.44 to 3.24)	5062 (3124 to 7077)	1.72 (1.06 to 2.41)	-0.97 (-1.16 to -0.77)	697589 (411528 to 1041067)	285.74 (168.57 to 426.43)	722003 (394256 to 1135053)	245.79 (134.21 to 386.40)	-0.43 (-0.64 to -0.22)
25–29 years	7088 (4616 to 9873)	3.22 (2.10 to 4.49)	6278 (4182 to 8563)	2.16 (1.44 to 2.94)	-1.30 (-1.48 to -1.11)	798940 (484168 to 1170093)	362.99 (219.98 to 531.62)	841511 (463847 to 1292978)	289.19 (159.40 to 444.34)	-0.70 (-0.96 to -0.44)
30–34 years	6602 (4536 to 9224)	3.47 (2.39 to 4.85)	8094 (5395 to 11135)	2.71 (1.80 to 3.72)	-0.85 (-1.15 to -0.54)	696430 (425243 to 1030417)	366.33 (223.68 to 542.01)	959866 (532297 to 1478842)	321.10 (178.07 to 494.71)	-0.39 (-0.60 to -0.19)
35–39 years	5014 (3457 to 7311)	2.89 (1.99 to 4.22)	7888 (5149 to 11339)	2.84 (1.85 to 4.08)	-0.01 (-0.34 to 0.33)	553522 (302676 to 839289)	319.12 (174.50 to 483.87)	900170 (502630 to 1384408)	324.03 (180.93 to 498.34)	0.10 (-0.16 to 0.35)
35–39 years	3641 (2461 to 5287)	2.60 (1.76 to 3.77)	7513 (4765 to 10907)	3.03 (1.92 to 4.40)	0.54 (0.33 to 0.76)	406152 (207813 to 629944)	289.64 (148.20 to 449.24)	797344 (434650 to 1252579)	321.39 (175.20 to 504.89)	0.38 (0.01 to 0.74)
45–49 years	2908 (1937 to 4216)	2.56 (1.70 to 3.70)	6418 (3827 to 9644)	2.72 (1.62 to 4.09)	0.20 (0.00 to 0.41)	307283 (147689 to 482313)	270.02 (129.78 to 423.82)	680602 (309217 to 1109315)	288.83 (131.22 to 470.76)	0.23 (-0.02 to 0.48)
SDI regions										
High SDI	3150 (2110 to 4433)	1.37 (0.91 to 1.94)	1898 (1231 to 2739)	0.77 (0.49 to 1.12)	-1.88 (-2.18 to -1.59)	455469 (203585 to 759143)	198.88 (89.48 to 331.12)	494771 (146597 to 949121)	202.50 (61.45 to 389.11)	0.08 (-0.26 to 0.42)
High-middle SDI	3750 (2415 to 5527)	1.35 (0.87 to 1.99)	2219 (1525 to 3016)	0.69 (0.47 to 0.94)	-1.99 (-2.83 to -1.14)	608960 (301794 to 984152)	219.14 (107.93 to 354.24)	514772 (223108 to 881988)	162.81 (72.45 to 278.12)	-0.87 (-1.21 to -0.53)
Middle SDI	8144 (5333 to 11482)	1.82 (1.20 to 2.55)	9549 (6460 to 13004)	1.51 (1.02 to 2.06)	-0.57 (-0.88 to -0.26)	1065977 (586242 to 1638237)	240.14 (130.02 to 370.61)	1265688 (674689 to 1983361)	201.05 (107.75 to 314.70)	-0.53 (-0.82 to -0.25)
Low-middle SDI	6863 (4162 to 10708)	2.57 (1.56 to 4.04)	15142 (9188 to 22576)	3.08 (1.87 to 4.61)	0.62 (0.40 to 0.84)	811249 (441062 to 1257847)	304.87 (162.16 to 476.87)	1647101 (894910 to 2574650)	331.94 (179.83 to 519.32)	0.28 (-0.02 to 0.59)
Low SDI	12428 (6557 to 20372)	12.17 (6.38 to 20.04)	15801 (9214 to 23906)	6.39 (3.69 to 9.78)	-2.08 (-2.57 to -1.60)	940119 (544986 to 1431313)	893.44 (512.91 to 1364.23)	1421483 (843231 to 2058998)	557.68 (328.46 to 809.74)	-1.54 (-1.96 to -1.11)
Regions										
Andean Latin America	194 (120 to 284)	2.03 (1.27 to 2.97)	279 (177 to 410)	1.59 (1.01 to 2.34)	-0.90 (-1.51 to -0.29)	22481 (11928 to 35122)	240.83 (124.73 to 379.77)	40422 (18305 to 68335)	230.22 (104.21 to 389.10)	-0.08 (-0.38 to 0.22)
Australasia	57 (35 to 84)	1.05 (0.66 to 1.56)	32 (20 to 47)	0.43 (0.26 to 0.64)	-2.98 (-4.77 to -1.15)	10794 (3711 to 20438)	199.78 (69.38 to 377.55)	13495 (2728 to 30008)	187.13 (38.59 to 417.67)	-0.16 (-0.42 to 0.10)
Caribbean	607 (367 to 925)	6.51 (3.96 to 9.95)	812 (474 to 1244)	6.74 (3.92 to 10.34)	0.09 (-0.29 to 0.47)	48839 (29718 to 72985)	519.23 (314.64 to 778.95)	66762 (38377 to 100887)	555.12 (318.86 to 839.24)	0.23 (-0.10 to 0.56)
Central Asia	220 (134 to 341)	1.36 (0.83 to 2.09)	122 (77 to 185)	0.49 (0.31 to 0.75)	-3.34 (-4.10 to -2.58)	21846 (12308 to 34861)	132.85 (74.78 to 211.19)	21935 (8416 to 41972)	88.07 (33.97 to 168.21)	-1.28 (-1.73 to -0.83)
Central Europe	254 (167 to 365)	0.81 (0.53 to 1.18)	69 (45 to 99)	0.26 (0.17 to 0.37)	-3.86 (-4.12 to -3.59)	37226 (17685 to 61936)	119.74 (57.05 to 199.14)	24891 (7382 to 48903)	91.73 (28.75 to 179.31)	-0.88 (-1.12 to -0.65)
Central Latin America	494 (324 to 734)	1.19 (0.79 to 1.74)	1082 (688 to 1618)	1.59 (1.01 to 2.37)	0.94 (0.07 to 1.81)	66502 (26955 to 117465)	164.32 (63.41 to 292.82)	146610 (57810 to 271480)	214.20 (84.90 to 396.12)	0.90 (0.26 to 1.54)
Central Sub-Saharan Africa	1827 (895 to 3303)	16.76 (8.20 to 30.27)	2629 (1425 to 4296)	9.31 (5.05 to 15.18)	-1.93 (-2.66 to -1.20)	145053 (77365 to 242903)	1274.79 (675.58 to 2141.43)	244756 (131211 to 385061)	823.49 (444.36 to 1289.46)	-1.46 (-1.86 to -1.06)
East Asia	3925 (2240 to 6216)	1.18 (0.68 to 1.86)	1343 (828 to 2001)	0.39 (0.24 to 0.59)	-3.54 (-3.76 to -3.32)	771613 (392926 to 1256972)	233.13 (117.54 to 380.06)	459154 (226179 to 761014)	135.07 (67.67 to 223.55)	-1.72 (-1.88 to -1.56)
Eastern Europe	1328 (836 to 2022)	2.34 (1.46 to 3.58)	1055 (718 to 1479)	1.93 (1.29 to 2.74)	-0.44 (-1.55 to 0.69)	132304 (71069 to 212179)	233.34 (125.16 to 375.55)	101675 (54453 to 162908)	191.08 (101.97 to 308.49)	-0.55 (-1.46 to 0.36)
Eastern Sub-Saharan Africa	10353 (5101 to 17777)	27.18 (13.26 to 46.75)	13312 (7217 to 21260)	14.32 (7.63 to 23.18)	-2.06 (-2.67 to -1.46)	711118 (382247 to 1150077)	1795.70 (957.64 to 2908.96)	1012709 (605980 to 1502834)	1050.61 (623.12 to 1569.20)	-1.73 (-2.32 to -1.13)
High-income Asia Pacific	121 (71 to 191)	0.27 (0.16 to 0.42)	55 (32 to 88)	0.14 (0.08 to 0.23)	-1.89 (-2.49 to -1.28)	34483 (10621 to 68779)	75.92 (23.48 to 151.15)	30469 (5869 to 68712)	79.60 (16.15 to 180.41)	0.20 (0.01 to 0.39)
High-income North America	2081 (1386 to 2979)	2.75 (1.81 to 3.97)	1239 (785 to 1840)	1.46 (0.92 to 2.19)	-2.07 (-2.45 to -1.68)	230052 (113731 to 373662)	305.62 (151.01 to 497.21)	240541 (74718 to 496944)	287.52 (89.70 to 593.23)	-0.10 (-0.43 to 0.23)
North Africa and Middle East	839 (537 to 1162)	1.09 (0.71 to 1.52)	2188 (1445 to 3130)	1.37 (0.90 to 1.96)	0.62 (0.05 to 1.19)	195822 (79270 to 345738)	261.08 (101.68 to 465.38)	460750 (180075 to 833634)	287.62 (112.79 to 520.14)	0.36 (0.00 to 0.72)
Oceania	30 (15 to 57)	1.96 (0.95 to 3.63)	161 (83 to 279)	4.67 (2.42 to 8.07)	2.80 (2.18 to 3.43)	4420 (2248 to 7256)	282.94 (143.78 to 461.88)	16214 (9101 to 25662)	466.63 (262.68 to 735.78)	1.60 (1.30 to 1.90)
South Asia	2603 (1465 to 4190)	0.99 (0.56 to 1.59)	4024 (2467 to 6008)	0.82 (0.50 to 1.22)	-0.58 (-1.03 to -0.13)	511235 (196436 to 924899)	204.46 (75.09 to 373.63)	951684 (314527 to 1757627)	194.54 (63.44 to 359.77)	-0.04 (-0.29 to 0.21)
Southeast Asia	1640 (1052 to 2382)	1.35 (0.88 to 1.95)	1494 (991 to 2105)	0.81 (0.53 to 1.14)	-1.64 (-2.09 to -1.20)	175342 (101617 to 265458)	144.83 (83.64 to 219.53)	210821 (106149 to 342662)	114.19 (57.63 to 185.55)	-0.75 (-1.01 to -0.48)
Southern Latin America	142 (97 to 203)	1.15 (0.79 to 1.64)	177 (112 to 259)	1.00 (0.63 to 1.48)	-0.35 (-0.84 to 0.15)	21640 (9109 to 39451)	175.19 (73.42 to 319.98)	30725 (11065 to 59615)	174.74 (63.31 to 339.10)	0.03 (-0.26 to 0.32)
Southern Sub-Saharan Africa	3406 (2182 to 5015)	26.31 (16.77 to 39.18)	5020 (2957 to 7716)	23.28 (13.66 to 35.89)	-0.50 (-1.10 to 0.10)	228944 (151185 to 327099)	1726.96 (1137.33 to 2489.62)	333424 (204466 to 494563)	1537.20 (939.55 to 2283.64)	-0.49 (-1.11 to 0.12)
Tropical Latin America	1008 (625 to 1553)	2.47 (1.56 to 3.78)	1379 (861 to 2079)	2.29 (1.41 to 3.48)	-0.29 (-0.71 to 0.13)	129929 (56060 to 227642)	326.48 (136.21 to 575.56)	157767 (72809 to 282935)	259.36 (121.00 to 462.02)	-0.67 (-0.98 to -0.37)
Western Europe	701 (487 to 938)	0.73 (0.50 to 0.97)	269 (185 to 363)	0.28 (0.19 to 0.38)	-3.03 (-3.98 to -2.07)	175264 (56771 to 328616)	181.04 (59.53 to 338.54)	187962 (31943 to 392145)	195.75 (35.65 to 407.92)	0.17 (-0.12 to 0.47)
Western Sub-Saharan Africa	2544 (1354 to 4295)	6.36 (3.34 to 10.79)	7920 (4462 to 12202)	7.72 (4.33 to 11.91)	0.59 (0.10 to 1.09)	210424 (121645 to 326178)	509.04 (290.35 to 792.87)	596182 (365379 to 874687)	556.63 (340.96 to 816.44)	0.27 (-0.46 to 1.00)

Rates are reported per 100,000 person-years. Data in parentheses are 95% uncertainty intervals for cases and age-standardized rates of mortality and DALYs, and 95% confidence intervals for AAPCs. IPV, Intimate Partner Violence; DALYs, disability-adjusted life-years; ASMR, age-standardized mortality rate; ASDR, age-standardized DALYs rate; AAPC, average annual percent change; SDI, socio-demographic index; UI, uncertainty interval; CI, confidence interval.

**Figure 2:**
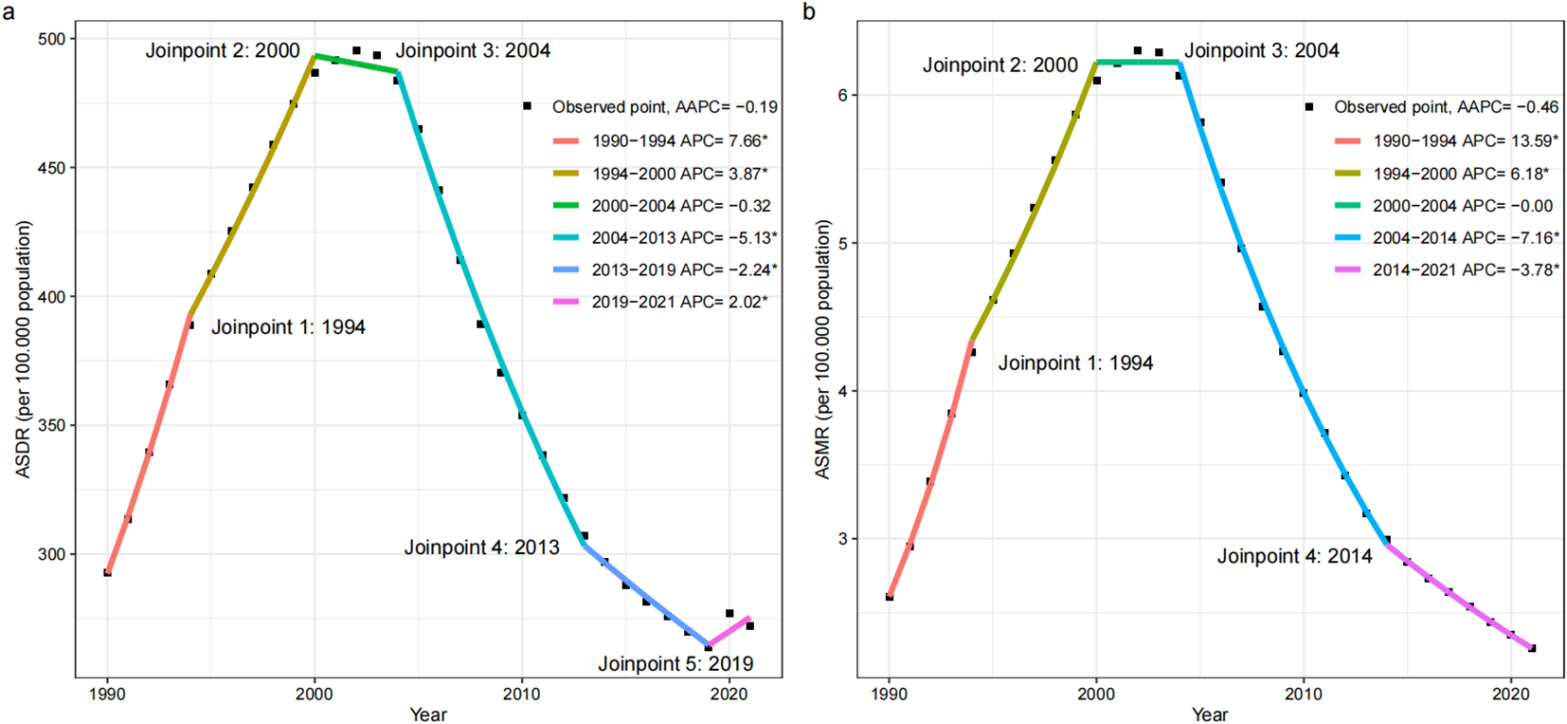
Joinpoint regression analysis in ASDR **(a)** and ASMR **(b)** of global disease burden attributable to IPV among women of childbearing age from 1990 to 2021. AAPC, average annual percent change; APC, annual percentage change; DALYs, disability-adjusted life-years; ASMR, age-standardized mortality rate; ASDR, age-standardized DALYs rate; IPV, Intimate Partner Violence.

The joinpoint regression analysis revealed significant inflection points in ASDR occurred in 1994, 2000, 2004, 2013, and 2019. The ASDR increased between 1990 and 2000, declined dramatically between 2000 and 2019, and increased at a relatively slow rate between 2019 and 2021 (APC= 2.01, 95% CI: 0.14 to 3.92, *P* = 0.036) (**Figure 2a**). The ASMR among WBCA followed a similar pattern, initially rising between 1990 and 2000 and then declining between 2000 and 2021, but no significant turning point was observed in 2019 (**Figure 2b**).

In 2021, depressive disorders accounted for the greatest disease burden attributable to IPV in terms of global DALY cases and ASDR. HIV/AIDS represented the greatest disease burden attributable to IPV in terms of global mortality cases and ASMR (**Supplementary Tables S1**-**S3**; **Figures 1c, d**). The ASDR due to IPV-related depressive disorders in 2021 was higher compared to 1990 (AAPC = 0.33, 95% CI: 0.17 to 0.48, *P =* 0.007). The joinpoint regression analysis revealed significant inflection point in 2019, indicating a significant increase in ASDR due to IPV-related depressive disorders after 2019 (APC= 12.96, 95% CI: 9.95 to 14.01, *P* < 0.007) (**Supplementary Table S1**, **Supplementary Figure S5a**). The ASDR and ASMR due to IPV-related HIV/AIDS as a whole showed an increasing trend, with a significant increase from 1990 to 2004, followed by a significant decrease from 2004 to 2021 (**Supplementary Table S2**, **Supplementary Figures S5g**, **S6a**). Contrarily, there was a downward trend for ASDR and ASMR due to IPV-related interpersonal violence (**Supplementary Table S3**, **Supplementary Figures S5m**, **S6g**).

### 3.2 Global burden trends by age group

**Figure 3:**
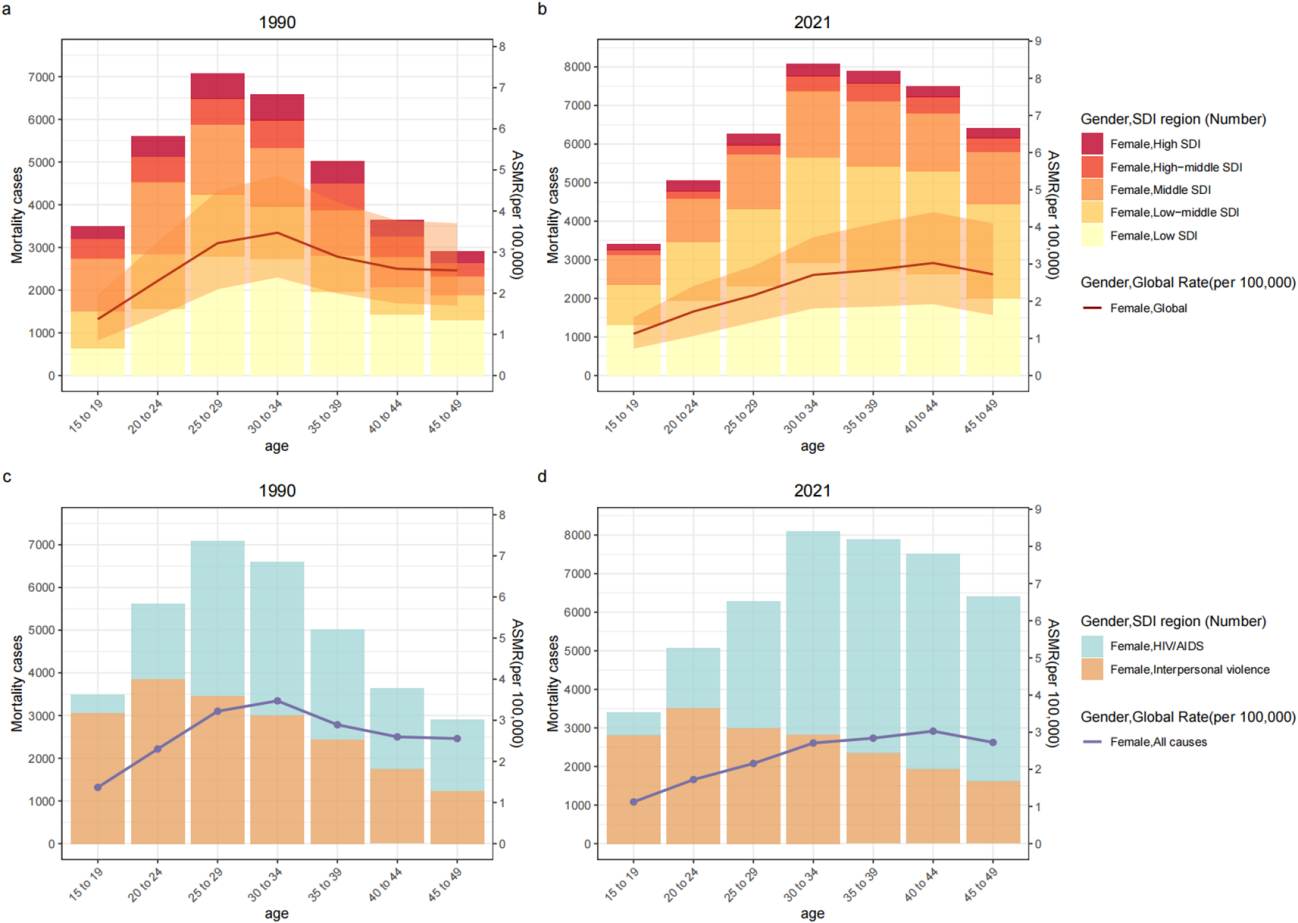
The disease burden attributable to IPV in age-specific mortality rates and numbers among women of childbearing age by SDI Regions **(a, b)** or Cause **(c, d)**, 1990 vs. 2021. DALYs, disability-adjusted life-years; SDI, sociodemographic index; ASDR, age-specific DALYs rate; IPV, Intimate Partner Violence.

Among WBCA, the peak age in the number of DALY and mortality across different age groups was at 25–29 years in 1990, shifting to 30–34 years by 2021. The peak age for the age-specific DALYs rate shifted from 30–34 years in 1990 to 35–39 years in 2021. The mortality pattern slightly differed from that of DALYs; the peak age for age-specific mortality rates shifted from 30–34 years in 1990 to 40–44 years in 2021 (**Table 1**, **Figures 3a, b**). Globally, from 1990 to 2021, the greatest increase in age-specific mortality and DALYs rates was observed in the 40–44 age group. The age-specific DALYs and mortality rates continued to increase in the 40–44 and 45–49 age groups, while they decreased in the remaining age groups (**Table 1**). Interestingly, the joinpoint regression analysis revealed a turning point in 2019 for the 15-19, 20-24, 25-29, and 30–34 age groups, indicating a significant increase in age-specific DALY rates after 2019 (APC in 15–19 age group = 2.01, 95% CI: 0.20 to 3.85, *P* = 0.031; APC in 20–24 age group = 4.03, 95% CI: 2.08 to 6.01, *P* < 0.001; APC in 25–29 age group = 3.08, 95% CI: 0.78 to 5.43, *P* = 0.021; APC in 30–34 age group = 1.89, 95% CI: 0.07 to 3.75, *P* = 0.042) (**Supplementary Figure S1**).

The burden of depressive disorders attributable to IPV was highest in the 35–39 age group, while the age-specific DALYs rate peaked in the 20–24 age group. Similarly, the burden of interpersonal violence attributable to IPV was greatest in the 20–24 age group, which also had the highest age-specific DALYs and mortality rates. The burden of HIV/AIDS attributable to IPV in terms of DALYs was most significant in the 30–34 age group. The mortality burden of HIV/AIDS attributable to IPV was highest in the 40–44 age group, which also had the highest age-specific DALYs and mortality rates (**Supplementary Tables S1**-**S3**; **Figures 3c, d**; **Supplementary Figures S7c**, **S7d**). The 15–19 age group exhibited the most significant increase the age-specific DALYs rate due to IPV-related depressive disorders from 1990 to 2021. Similarly, all age groups in all but the 25–29 age group showed significant and rapid growth from 2019 to 2021. Among them, 15–19 age group has made the largest increases (**Supplementary Table S1**, **Supplementary Figures S2a–g**). The age-specific DALYs and mortality rates due to IPV-related interpersonal violence have demonstrated a downward trend in all age groups (**Supplementary Table S3**, **Supplementary Figures S2o-u**, **S3h–n**). For age-specific DALYs and mortality rates due to IPV-related HIV/AIDS, the 15–19 age group witnessed the most rapid increase, while the 25–29 age group had the fastest decline (**Supplementary Table S2**, **Supplementary Figures S2h–n**, **S3a–g**).

### 3.3 Burden trends association with the SDI

The low and low-middle SDI regions had the highest cases of IPV-attributable mortality and DALYs per year and per age bracket, indicating the greatest disease burden attributable to IPV among WBCA, while the highest ASDR and ASMR were reported in the low SDI region in both 1990 and 2021 (**Figure 1**, **Table 1**). The low SDI region had the fastest decrease in ASMR and ASDR, with AAPCs of -2.08 (95% CI: -2.57 to -1.60) and -1.54 (95% CI: -1.96 to -1.11), respectively, while a significant upward trend of ASMR was found only in the low-middle SDI region, with AAPC of 0.62 (95% CI: 0.40 to 0.84) (**Table 1**, **Supplementary Figure S4**).

In 2021, the ASDR and ASMR for HIV/AIDS and interpersonal violence attributable to IPV were highest in low SDI regions, while the ASDR for depressive disorders attributable to IPV was greatest in high SDI regions. The burden of interpersonal violence attributable to IPV was most prominent in low-middle SDI regions. Middle SDI regions remained notably the highest in interpersonal violence burden attributable to IPV. Furthermore, low SDI regions bore the greatest burden of HIV/AIDS attributable to IPV (**Supplementary Tables S1**-**S3**). The high SDI region exhibited the most significant increase in ASDR due to IPV-related depressive disorders from 1990 to 2021. At the same time, the high SDI, high-middle SDI, low-middle SDI, and middle SDI regions all showed significant and rapid growth from 2019 to 2021, with the corresponding APCs all greater than 10 (**Supplementary Table S1**, **Supplementary Figures S5b–f**). For ASMR and ASDR due to IPV-related HIV/AIDS, the middle SDI region witnessed the most rapid increase, while the high SDI region had the fastest decline (**Supplementary Table S2**, **Supplementary Figures S5h–l**, **S6b–f**). The ASMR and ASDR due to IPV-related interpersonal violence in the five SDI regions were in a downward trend, with the largest decrease in high-middle SDI region (**Supplementary Table S3**, **Supplementary Figures S5n–r**, **S6h–l**).

### 3.4 Burden trends by GBD region and country

**Figure 4:**
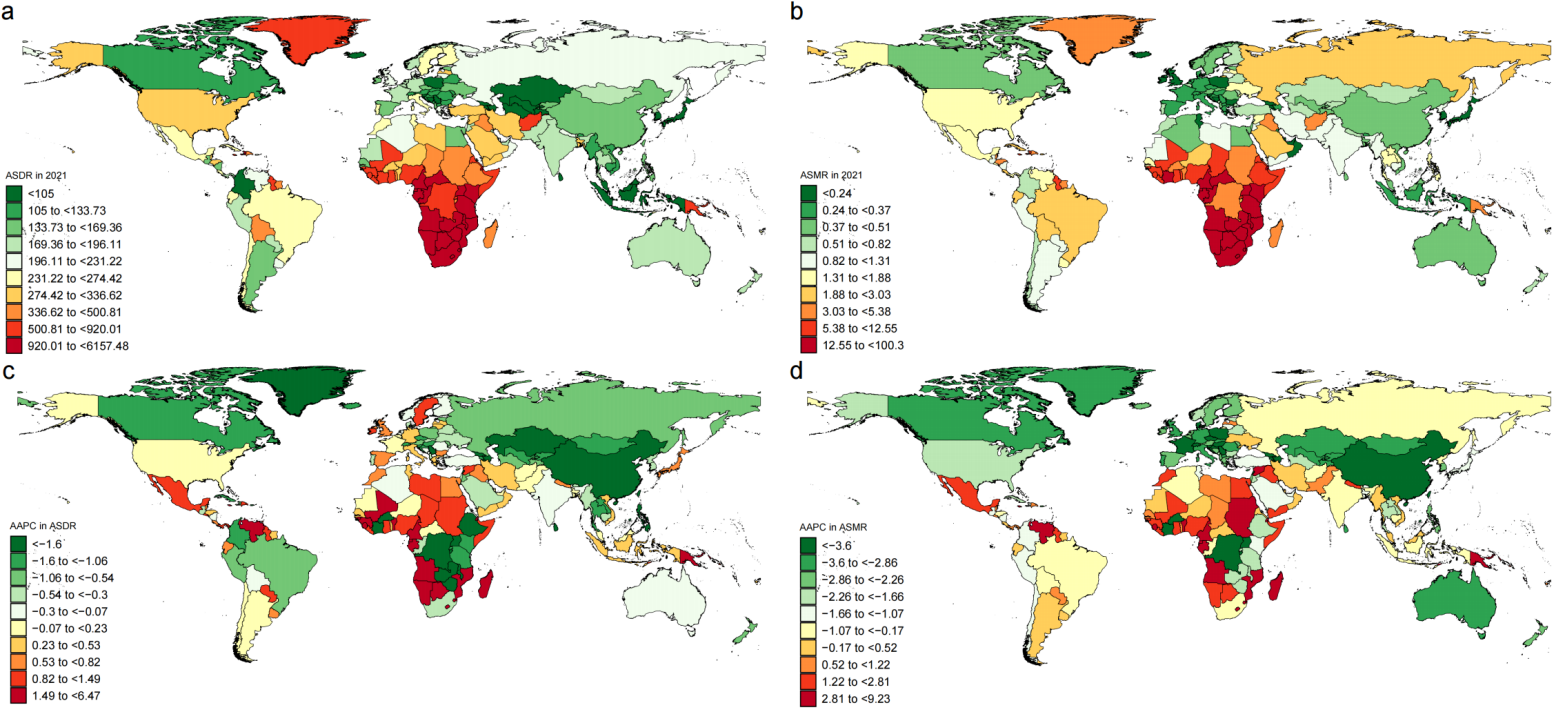
ASDR and ASMR in 2021 **(a, b)**, and their AAPC from 1990 to 2021 **(c, d)** for the disease burden attributable to IPV among women of childbearing age, by country. AAPC, average annual percent change; DALYs, disability-adjusted life-years; ASMR, age-standardized mortality rate; ASDR, age-standardized DALYs rate; IPV, Intimate Partner Violence.

At the regional level, Oceania exhibited the most significant upward trend in ASMR and ASDR from 1990 to 2021, with the corresponding AAPCs being 2.80 (95% Cl: 2.18 to 3.43, *P* < 0.001) and 1.60 (95% Cl: 1.30 to 1.90, *P* < 0.001), respectively. In 1990, Eastern Sub-Saharan Africa reported the highest ASMR and ASDR. In contrast, by 2021, Southern Sub-Saharan Africa had become the region with the highest ASMR and ASDR. At the same time, Eastern Sub-Saharan Africa had the highest number of cases of IPV-attributable mortality and DALYs in 2021, becoming the region with the greatest disease burden attributable to IPV among WBCA (**Table 1**). In 2021, the United States of America ranked fourth following India, China, and Nigeria in terms of the number of IPV-attributable DALYs, while Nigeria, India, and South Africa were the top three in terms of the number of IPV-attributable mortality. Nigeria had the highest ASDR and ASMR attributable to IPV (**Supplementary Table S4**; **Figure 4**).

HIV/AIDS and depressive disorders attributable to IPV had the highest burden in South Asia, while the burden of HIV/AIDS attributable to IPV was greatest in Eastern Sub-Saharan Africa. Southern Sub-Saharan Africa recorded the highest ASDR and ASMR for HIV/AIDS and violence attributable to IPV, whereas Central Sub-Saharan Africa had the highest ASDR for depressive disorders attributable to IPV (**Supplementary Tables S1**-**S3**, **Supplementary Figures S8**-**S10**). High-income North America exhibited the most significant increase in ASDR due to IPV-related depressive disorders from 1990 to 2021. At the same time, Central Europe, Tropical Latin America, and High-income North America were the top three regions with the most significant rapid growth from 2019 to 2021. For ASMR and ASDR due to IPV-related HIV/AIDS, South Asia witnessed the most rapid increase, while High-income North America had the fastest decline. Oceania exhibited the most significant increase in ASDR due to IPV-related interpersonal violence from 1990 to 2021 (**Supplementary Tables S1**-**S3**, **Supplementary Figures S8**-**S10**).

### 3.5 Health inequality

**Figure 5:**
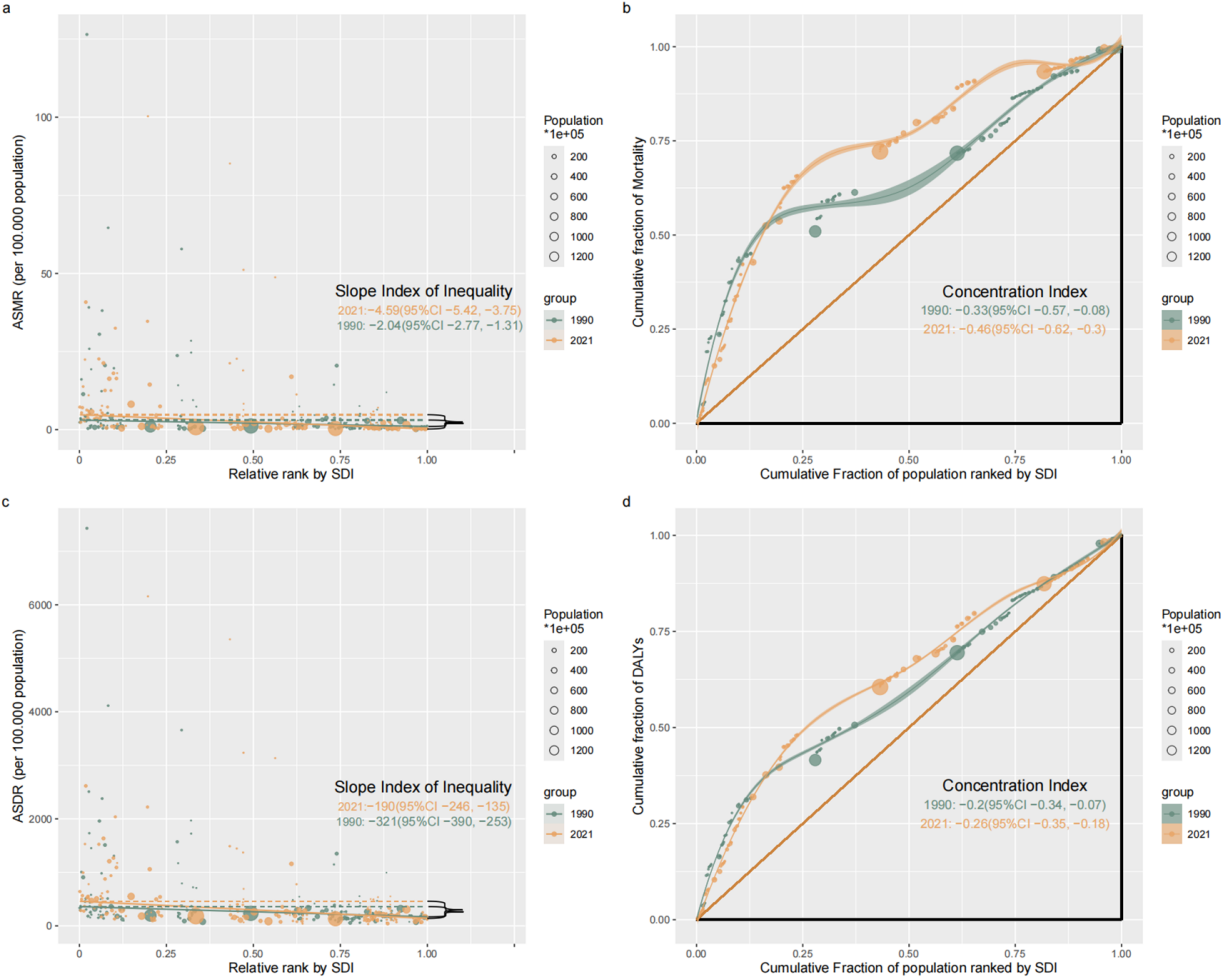
Absolute healthy inequality **(a, c)** and relative healthy inequality **(b, d)** for ASMR **(a, b)** and ASMR **(c, d)** of the disease burden attributable to IPV among women of childbearing age, 1990 vs. 2021. DALYs, disability-adjusted life-years; ASDR, age-standardized DALYs rate; ASMR, age-standardized mortality rate; IPV, Intimate Partner Violence; SDI, Socio-demographic index.

Remarkable absolute and relative SDI-related inequalities in the disease burden attributable to IPV were detected. The SII of ASDR was −321 (95% CI: −390 to −253) and −190 (95% CI: −246 to −135) in 1990 and 2021; the SII of ASMR was −2.04 (95% CI: −2.77 to −1.31) and −4.59 (95% CI: −5.42 to −3.75) in 1990 and 2021, respectively. This indicated that the absolute inequality in the ASMR burden attributable to IPV among WBCA between the highest and lowest SDI countries has significantly increased over the past 32 years, while the absolute inequality in ASDR burden has decreased (**Figures 5a, c**). The concentration index of ASDR and ASMR remained increasing between 1990 (ASDR = -0.2, 95% CI: −0.34 to −0.07; ASMR = -0.33, 95% CI: −0.57 to −0.08) and 2021 (ASDR = −0.26, 95% CI: −0.35 to −0.18; ASMR = -0.46, 95% CI: −0.62 to −0.30) (**Figure 5c**). Meanwhile, the relative inequality for ASDR and ASMR burden has become more excessively concentrated in poorer countries and tends towards deterioration (**Figures 5b, d**).

However, the change in SII varied across the different diseases attributable to IPV. Between 1990 and 2021, the SII for HIV/AIDS and interpersonal violence attributable to IPV increased (**Supplementary Figures S11a**, **S11b**, **S12a**, **S12b**), while that for depressive disorders decreased (**Supplementary Figure S11c**). By contrast, relative inequality in interpersonal violence and depressive disorders attributable to IPV remained stable (**Supplementary Figures S11e**, **S11f**, **S12d**). The concentration index for HIV/AIDS attributable to IPV has improved slightly over the past 32 years. However, the absolute value of the concentration index for HIV/AIDS attributable to IPV remained over 0.6, indicating that the high burden persists in poorer countries (**Supplementary Figures S11d**, **S12c**).

## 4 Discussion

This secondary analysis of data from the GBD 2021 provides an updated and comprehensive overview of the global disease burden attributable to IPV among WBCA, health inequalities, and temporal trends from 1990 to 2021. Our study demonstrated that, despite a decrease in the global ASMR and ASDR burden attributable to IPV between 1990 and 2021, after the COVID-19 pandemic, the disease burden attributable to IPV began to increase, and the upward trend mainly focused on depressive disorders caused by IPV. The overall burden continues to increase, and health inequalities remain severe and excessively concentrated in low-income countries. This inequality is primarily attributable to HIV/AIDS caused by IPV.

### 4.1 Disease burden

First, this study explored changes in disease burden attributable to IPV through a joinpoint regression model. The disease burden attributable to IPV exhibited an overall trend of initially rising sharply, then decreasing, and finally increasing again over time. There were two critical turning points on the trendline including around the years 2000 and 2019. The most important reason for the first shift in trends was the implementation of IPV-related policies and legislation. In 1993, the United Nations General Assembly adopted the “Declaration on the Elimination of Violence Against Women”. In 1999, the United Nations General Assembly passed a resolution designating November 25th of each year as the International Day for the Elimination of Violence against Women, aiming to raise awareness of violence against women and calling for action to eliminate such violence.

However, the second shift in trends revealed the substantial impact of the COVID-19 pandemic on the disease burden attributable to IPV. Although isolation was an effective measure to curb the spread of COVID-19, it also had significant social, economic, and psychological impacts. We found that the second shift in trends was concentrated among depressive disorders caused by IPV in our study. During the COVID-19 pandemic, the rise in IPV was closely linked to the occurrence of depression among women. Social isolation caused by lockdowns (22), economic pressure (23, 24), disruption of mental health services (25), and increased household responsibilities (26) have all exacerbated psychological stress for IPV victims, leading to a higher incidence of depression. Additionally, studies have shown that longer isolation periods increase the likelihood of severe psychological issues (27). The burden of depressive disorders attributable to IPV has become evident not only in economically vulnerable regions but also in developed countries (26). Furthermore, we found that most regions and populations exhibited an increasing trend, with high-income areas in North America, regions with a high SDI, and the 15–19 age group showing the highest and fastest rise. This suggests that more WBCA are suffering from depressive disorders related to IPV following the COVID-19 pandemic.

A modeling analysis of 46 African countries indicated that addressing IPV could be key to eliminating HIV vertical transmission, as one-eighth of pediatric HIV infections in sub-Saharan Africa were attributable to IPV, particularly affecting young women who face the highest burden of violence (28). Our study also found substantial growth in the HIV/AIDS burden attributable to IPV before 2004, followed by a significant decrease. This reduction coincided with efforts in 2004, when the WHO, the Joint United Nations Programme on HIV/AIDS, and the Global Fund collaborated to expand the availability of antiretroviral therapy in developing countries. The expansion of HIV treatment has since led to viral load suppression in nearly three-quarters of people living with HIV worldwide and has significantly reduced the burden of HIV-related deaths (29). However, there is little evidence in the current literature about the impact of the COVID-19 pandemic on the HIV/AIDS burden caused by IPV. Among the regions studied, the largest increase in HIV/AIDS burden attributable to IPV was observed in South Asia and among the 15-19-year age group. While high-burden areas in Africa, as well as low-SDI and low-middle-SDI regions, receive the most research funding and attention, far more people in these areas continue to suffer the highest burden of HIV/AIDS related to IPV.

Encouragingly, the ASDR and ASMR burden of interpersonal violence attributable to IPV has been in decline since the 1990s, largely due to socioeconomic development and improvements in healthcare services. However, this progress proved insufficient in the context of the COVID-19 pandemic. During the pandemic, epidemic prevention and control led to prolonged contact between perpetrators and victims, leading to an increase in violence, while reporting to the police and access to social services decreased (26).

### 4.2 Health inequality

This study shows that the disease burden attributable to IPV is closely related to the SDI of a country, which is consistent with previous reports (30). Low-SDI countries have made some progress in reducing the disease burden attributable to IPV, partially due to interventions led by multilateral organizations such as the WHO. However, despite these achievements, the inequality in the disease burden attributable to IPV remains severe globally.

By calculating the SII and Concentration Index, we quantitatively assessed the inequality trends in the disease burden attributable to IPV. The absolute inequality measured by SII reflects the absolute magnitude of differences in the disease burden attributable to IPV, indicating that the burden gap between high-SDI and low-SDI countries is widening. The Concentration Index shows that the IPV burden is disproportionately concentrated in poor countries. Since 1991, the absolute value of the Concentration Index has consistently been above 0.2, indicating that the inequality in the IPV burden has reached a moderate-to-high level and has worsened over this period (31). Of particular concern is that the Concentration Index for the HIV/AIDS burden attributable to IPV is even higher in low-SDI countries, consistently above 0.6, indicating significant inequalities in HIV health in these countries.

Notably, low-SDI countries continue to bear the heaviest IPV burden and health inequalities, which are closely linked to their specific socioeconomic and cultural contexts. First, economic pressure is a major factor aggravating the disease burden attributable to IPV in low-SDI countries. Due to poverty, high unemployment, and income instability, economic tension often exacerbates conflicts between partners (32). Secondly, the low level of education in low-SDI countries leads to a lack of effective communication and conflict resolution skills, making individuals more susceptible to traditional gender norms, resulting in more prevalent violent behavior (33). Moreover, traditional gender roles and the emphasis on male authority in low-SDI countries make women more susceptible to control and violence. In some cultural contexts, IPV is seen as “justified” or as a “domestic matter,” limiting external interventions (34). Finally, the weak legal systems and social support structures in low-SDI countries further exacerbate the disease burden attributable to IPV (35). In conclusion, low-SDI countries continue to bear the heaviest disease burden attributable to IPV, a phenomenon closely tied to poverty, low educational attainment, gender inequality in culture, and weak legal and social support systems.

### 4.3 IPV research in the post-COVID-19 pandemic era

This research has significant policy and public health implications in the post-pandemic era. The COVID-19 pandemic has revealed persistent challenges related to IPV health inequalities, and our data indicate that this is not a temporary phenomenon but will have long-term impacts on women’s health globally. At the global public health policy level, governments must incorporate IPV prevention into core post-pandemic recovery agendas, particularly establishing emergency response mechanisms for crisis periods. Our findings of significant increase in IPV-related depression burden among young women aged 15-19, as well as worsening health inequalities in low-SDI countries, provide compelling evidence for international health organizations and policymakers, supporting the prioritization of limited resources for the most vulnerable populations. In conclusion, this study not only quantifies trends in the global IPV disease burden but also establishes an empirical foundation for developing more resilient and equitable global public health systems in the post-pandemic era.

### 4.4 Limitation

First, the estimation of the IPV burden among women of reproductive age largely depends on the availability and quality of GBD 2021 data. The GBD database relies on diverse data sources worldwide, including hospital records, death registries, censuses, health surveys, and academic research. However, the availability and quality of data vary significantly across countries and regions, particularly in resource-limited countries or regions, where incomplete or low-quality data are particularly prominent issues. This can affect the accuracy of the model’s estimation results (17, 19). Furthermore, it is important to note that in the GBD 2021 study, the burden estimation of IPV is limited to female populations, which is due to the lack of sufficient risk-outcome evidence for male IPV within the GBD 2021 study framework. Although men may also experience IPV, the existing GBD 2021 data cannot reflect the disease burden of IPV experienced by males, which may lead to an underestimation of the overall global impact of IPV. Secondly, our study specifically aims to describe the burden of three common IPV-attributable diseases among women of reproductive age: HIV/AIDS, depressive disorders, and interpersonal violence. Other IPV-related diseases, such as obesity (36), anxiety (37), PTSD (38), schizophrenia (39), and self-harm (40), are not included in the GBD 2021 database. Future research should investigate these additional disease burdens attributable to IPV in greater depth. Additionally, IPV includes various forms of abusive behaviors, such as physical violence and sexual violence, psychological violence, and economic violence (2). However, the existing GBD data only provide information on physical and sexual violence and do not adequately capture the associations between other forms of IPV and related health outcomes. Finally, although the GBD database provides a wealth of global and regional health data, it offers insufficient detail regarding regional granularity and population stratification in some cases. For example, GBD data often focus on the overall disease burden of a country or region, overlooking differences between sub-populations within a country, such as ethnic minorities or specific genders, thereby limiting the identification of inequalities at the micro level and leads to insufficiently targeted policy interventions.

Based on these limitations, we propose that future research directions should focus on addressing these gaps in data and methodology. While this study comprehensively assessed the impact of IPV on depression, future research should explore more deeply the associations between IPV and other mental health outcomes, particularly PTSD, anxiety disorders, and suicidal behaviors, which may be affected by various forms of IPV. Establishing longitudinal cohort studies with standardized mental health assessment tools would help elucidate causal relationships and mediating mechanisms between violence exposure and these outcomes. Additionally, future research should strive to collect more detailed data on sub-populations, including vulnerable groups with different sexual orientations, gender identities, immigration status, and disabilities, as well as considering interactions between race and socioeconomic status. These granular data would help identify high-risk populations and inform the development of targeted prevention and intervention strategies for specific groups.

## 5 Conclusion

In conclusion, IPV has emerged as a significant global public health challenge, particularly in its contribution to the disease burden among WBCA. Although the overall burden attributable to IPV has decreased since 1990, the COVID-19 pandemic has significantly reversed this trend, particularly in the burden of IPV-related depressive disorders. Moreover, health inequalities remain severe, particularly in LMICs where the burden of IPV-related HIV/AIDS is especially pronounced. Therefore, coordinated global health policy efforts are urgently required, particularly for WBCA in low-income countries, to reduce the adverse health impacts of IPV and address the escalating health inequalities. Such efforts will also contribute to achieving a key target of the United Nations’ 2030 Sustainable Development Goals: specifically the elimination of all forms of violence against women and girls in both public and private spheres.

## Data Availability

The original contributions presented in the study are included in the article/**Supplementary Material**. Further inquiries can be directed to the corresponding author.
