# Supplementary material for: Persistent burden and health inequalities of disease in women of childbearing age attributable to Intimate Partner Violence, 1990–2021
Source: Front Psychiatry. 2025 Aug 20;16:1515828. doi: 10.3389/fpsyt.2025.1515828 (PMC12405915; doi:10.3389/fpsyt.2025.1515828)
Supplement: Supplementary file 1 [file Supplementaryfile1.docx]

***Supplementary Material***

**Persistent burden and health inequalities of disease in women of childbearing age attributable to Intimate partner violence, 1990–2021**

**Table S1. Age-standardised mortality rates and DALY rates in 1990 and 2021, and AAPC from 1990 to 2021 for the depressive disorders burden attributable to IPV among women of childbearing age , by SDI quintile, region level.and country.**

| **Location** | **DALYs** | | | | |
| --- | --- | --- | --- | --- | --- |
|  | **DALYs cases in 1990 (95% UI)** | **ASDR in 1990 (95% UI)** | **DALYs cases in 2021 (95% UI)** | **ASDR in 2021 (95% UI)** | **AAPC% (95%CI),**  **1990–2021** |
| **Global** | 1161458(3824 to 2731957) | 90.48(0.30 to 211.99) | 1942200(7654 to 4503653) | 98.20(0.39 to 227.97) | 0.33(0.17 to 0.48) |
| **Age** | | | | | |
| **15-19 years** | 59801(110 to 154112) | 23.40(0.04 to 60.31) | 95322(213 to 227947) | 31.39(0.07 to 75.07) | 1.16(1.00 to 1.32) |
| **20-24 years** | 169715(488 to 413427) | 69.52(0.20 to 169.34) | 241149(987 to 598734) | 82.09(0.34 to 203.82) | 0.65(0.55 to 0.75) |
| **25-29 years** | 220859(798 to 525732) | 100.34(0.36 to 238.86) | 299147(1251 to 710949) | 102.80(0.43 to 244.32) | 0.11(-0.41 to 0.64) |
| **30-34 years** | 208506(866 to 494077) | 109.68(0.46 to 259.89) | 332977(1140 to 795307) | 111.39(0.38 to 266.05) | 0.12(-0.03 to 0.28) |
| **35-39 years** | 198404(615 to 448775) | 114.38(0.35 to 258.73) | 343955(1542 to 765174) | 123.81(0.55 to 275.44) | 0.30(0.03 to 0.56) |
| **35-39 years** | 168326(564 to 397176) | 120.04(0.40 to 283.24) | 324044(1514 to 714568) | 130.62(0.61 to 288.03) | 0.33(0.20 to 0.45) |
| **45-49 years** | 135848(383 to 298657) | 119.37(0.34 to 262.44) | 305606(1006 to 690974) | 129.69(0.43 to 293.23) | 0.26(-0.01 to 0.52) |
| **SDI regions** | | | | | |
| **High SDI** | 213576(723 to 497126) | 92.19(0.31 to 214.87) | 324670(1139 to 768730) | 131.23(0.46 to 312.83) | 1.30(1.15 to 1.45) |
| **High-middle SDI** | 225773(619 to 539410) | 82.53(0.22 to 196.90) | 247426(883 to 591048) | 75.41(0.27 to 181.38) | -0.31(-0.60 to -0.01) |
| **Middle SDI** | 319249(1035 to 766527) | 76.83(0.25 to 182.95) | 461075(1526 to 1098001) | 71.74(0.24 to 171.48) | -0.14(-0.29 to 0.01) |
| **Low-middle SDI** | 270565(946 to 655889) | 107.84(0.37 to 259.33) | 579237(2452 to 1382658) | 117.50(0.50 to 279.51) | 0.33(0.11 to 0.54) |
| **Low SDI** | 131430(565 to 314205) | 129.18(0.57 to 305.63) | 328361(1643 to 783468) | 130.89(0.65 to 310.29) | 0.03(-0.28 to 0.33) |
| **Regions** | | | | | |
| **Andean Latin America** | 7418(39 to 18161) | 86.22(0.46 to 209.47) | 18231(103 to 45154) | 103.70(0.59 to 256.92) | 0.74(0.16 to 1.33) |
| **Australasia** | 6300(32 to 15929) | 115.67(0.59 to 293.03) | 10244(47 to 26776) | 141.25(0.64 to 372.14) | 0.72(0.54 to 0.90) |
| **Caribbean** | 8455(29 to 21836) | 95.19(0.32 to 244.78) | 13258(58 to 33388) | 109.12(0.48 to 274.97) | 0.55(0.44 to 0.66) |
| **Central Asia** | 5712(22 to 14928) | 35.90(0.14 to 93.17) | 11643(45 to 31193) | 46.34(0.18 to 124.29) | 0.93(0.85 to 1.00) |
| **Central Europe** | 15470(60 to 37672) | 49.17(0.19 to 120.17) | 16207(63 to 39620) | 57.76(0.22 to 142.84) | 0.58(0.48 to 0.68) |
| **Central Latin America** | 32851(149 to 79913) | 86.13(0.40 to 206.83) | 76606(289 to 193263) | 111.27(0.42 to 280.91) | 1.00(0.50 to 1.51) |
| **Central Sub-Saharan Africa** | 26854(145 to 64790) | 235.26(1.29 to 562.58) | 69168(356 to 164094) | 227.17(1.17 to 535.23) | -0.04(-0.28 to 0.20) |
| **East Asia** | 235323(819 to 592191) | 74.97(0.26 to 187.35) | 153995(339 to 394703) | 40.67(0.09 to 105.82) | -1.94(-2.14 to -1.74) |
| **Eastern Europe** | 41260(95 to 103706) | 71.71(0.16 to 180.59) | 34909(80 to 91072) | 64.30(0.16 to 169.07) | -0.35(-0.51 to -0.20) |
| **Eastern Sub-Saharan Africa** | 56089(260 to 133888) | 147.41(0.71 to 349.02) | 142012(648 to 339969) | 146.59(0.68 to 347.72) | 0.02(-0.06 to 0.10) |
| **High-income Asia Pacific** | 20873(64 to 53626) | 45.76(0.14 to 117.52) | 22954(36 to 60695) | 58.94(0.10 to 158.10) | 0.88(0.77 to 0.99) |
| **High-income North America** | 92882(349 to 218634) | 122.17(0.45 to 288.47) | 153671(636 to 402454) | 183.02(0.76 to 479.67) | 1.43(1.00 to 1.86) |
| **North Africa and Middle East** | 102394(341 to 251579) | 141.97(0.47 to 345.84) | 249154(1097 to 603444) | 155.00(0.68 to 375.75) | 0.44(0.30 to 0.57) |
| **Oceania** | 1293(6 to 3230) | 86.86(0.43 to 213.90) | 3223(19 to 7826) | 93.43(0.55 to 226.50) | 0.21(0.06 to 0.37) |
| **South Asia** | 245042(792 to 605050) | 103.75(0.33 to 253.91) | 554153(2465 to 1334366) | 114.47(0.51 to 274.85) | 0.33(-0.03 to 0.69) |
| **Southeast Asia** | 40550(94 to 101937) | 35.77(0.09 to 89.53) | 77502(198 to 195325) | 41.48(0.10 to 104.80) | 0.48(0.44 to 0.52) |
| **Southern Latin America** | 10878(48 to 27907) | 88.56(0.39 to 227.18) | 17374(72 to 45252) | 97.90(0.41 to 256.04) | 0.35(-0.07 to 0.76) |
| **Southern Sub-Saharan Africa** | 6811(25 to 17984) | 56.84(0.21 to 149.73) | 15460(64 to 40415) | 71.32(0.29 to 186.15) | 0.80(0.65 to 0.94) |
| **Tropical Latin America** | 59928(261 to 145776) | 157.57(0.69 to 379.89) | 65872(232 to 181243) | 104.80(0.36 to 288.89) | -1.22(-1.38 to -1.06) |
| **Western Europe** | 110363(303 to 259406) | 112.85(0.30 to 265.61) | 150970(583 to 354448) | 154.76(0.59 to 366.42) | 1.21(1.08 to 1.35) |
| **Western Sub-Saharan Africa** | 34711(118 to 85672) | 88.82(0.30 to 216.21) | 85594(364 to 208425) | 79.43(0.34 to 191.93) | -0.28(-0.57 to 0.00) |
| **Countries** | | | | | |
| **Afghanistan** | 3722(22 to 10072) | 196.82(1.14 to 522.77) | 15356(90 to 39191) | 242.37(1.44 to 605.49) | 0.75(0.66 to 0.83) |
| **Albania** | 491(3 to 1332) | 62.43(0.37 to 167.49) | 582(3 to 1616) | 91.51(0.50 to 255.55) | 1.40(1.23 to 1.58) |
| **Algeria** | 6548(35 to 19616) | 127.00(0.66 to 374.91) | 16315(84 to 47374) | 139.29(0.72 to 407.33) | 0.44(0.20 to 0.68) |
| **American Samoa** | 7(0 to 19) | 60.28(0.35 to 160.00) | 8(0 to 21) | 68.77(0.36 to 186.92) | 0.39(0.27 to 0.51) |
| **Andorra** | 14(0 to 44) | 91.49(0.42 to 281.60) | 25(0 to 75) | 114.68(0.55 to 355.96) | 0.95(0.52 to 1.38) |
| **Angola** | 3843(19 to 11277) | 181.25(0.87 to 527.40) | 14069(63 to 37984) | 197.95(0.89 to 531.50) | 0.31(0.12 to 0.51) |
| **Antigua and Barbuda** | 10(0 to 31) | 64.32(0.31 to 195.72) | 21(0 to 66) | 83.53(0.37 to 261.80) | 0.91(0.86 to 0.96) |
| **Argentina** | 4079(16 to 12801) | 51.46(0.21 to 161.42) | 7263(33 to 23652) | 60.07(0.27 to 196.55) | 0.53(0.10 to 0.96) |
| **Armenia** | 137(1 to 520) | 16.62(0.07 to 63.36) | 188(1 to 576) | 22.99(0.11 to 71.63) | 1.28(1.03 to 1.53) |
| **Australia** | 4949(27 to 12681) | 108.82(0.60 to 279.76) | 8557(41 to 23218) | 141.29(0.68 to 387.50) | 1.00(0.79 to 1.20) |
| **Austria** | 1362(7 to 4187) | 66.77(0.31 to 205.67) | 1278(6 to 3657) | 61.57(0.29 to 177.49) | -0.17(-0.49 to 0.16) |
| **Azerbaijan** | 888(5 to 2496) | 47.04(0.25 to 133.41) | 1250(6 to 3879) | 44.70(0.21 to 139.98) | -0.08(-0.32 to 0.16) |
| **Bahamas** | 46(0 to 143) | 66.36(0.35 to 205.46) | 95(0 to 296) | 86.67(0.31 to 270.37) | 1.06(0.81 to 1.30) |
| **Bahrain** | 180(1 to 532) | 161.01(0.76 to 469.04) | 573(3 to 1706) | 171.07(0.78 to 510.48) | 0.27(0.20 to 0.35) |
| **Bangladesh** | 48138(285 to 121016) | 216.17(1.29 to 535.32) | 103981(556 to 262192) | 229.24(1.23 to 576.28) | 0.24(-0.01 to 0.49) |
| **Barbados** | 44(0 to 134) | 64.77(0.25 to 197.20) | 66(0 to 204) | 87.76(0.43 to 274.78) | 1.00(0.92 to 1.07) |
| **Belarus** | 1083(5 to 3590) | 41.69(0.18 to 138.17) | 1526(7 to 4226) | 60.09(0.29 to 167.62) | 1.18(1.10 to 1.26) |
| **Belgium** | 2202(11 to 5938) | 87.55(0.42 to 237.21) | 3280(17 to 8882) | 126.34(0.64 to 344.16) | 1.54(0.82 to 2.27) |
| **Belize** | 13(0 to 43) | 34.21(0.13 to 116.14) | 51(0 to 167) | 43.84(0.21 to 143.03) | 1.00(0.67 to 1.34) |
| **Benin** | 833(4 to 2551) | 84.98(0.44 to 256.67) | 2600(13 to 6929) | 90.50(0.46 to 237.92) | 0.25(0.19 to 0.31) |
| **Bermuda** | 17(0 to 49) | 95.92(0.42 to 275.64) | 16(0 to 46) | 107.69(0.60 to 321.60) | 0.44(0.40 to 0.48) |
| **Bhutan** | 104(0 to 317) | 89.25(0.42 to 268.68) | 164(1 to 470) | 80.65(0.40 to 231.26) | -0.25(-0.40 to -0.10) |
| **Bolivia (Plurinational State of)** | 2112(13 to 5256) | 151.63(0.92 to 373.58) | 5450(30 to 13943) | 177.15(0.96 to 452.69) | 0.62(0.05 to 1.19) |
| **Bosnia and Herzegovina** | 815(4 to 2407) | 70.02(0.33 to 206.95) | 587(3 to 1801) | 74.54(0.34 to 230.75) | 0.31(0.20 to 0.42) |
| **Botswana** | 419(3 to 1065) | 148.37(0.93 to 370.67) | 1334(9 to 3464) | 195.24(1.31 to 507.57) | 0.83(0.72 to 0.93) |
| **Brazil** | 59511(259 to 144836) | 160.15(0.70 to 386.32) | 64181(224 to 177093) | 105.29(0.36 to 291.24) | -1.25(-1.41 to -1.09) |
| **Brunei Darussalam** | 23(0 to 72) | 33.97(0.16 to 104.96) | 54(0 to 164) | 41.37(0.22 to 127.04) | 0.69(0.61 to 0.78) |
| **Bulgaria** | 1504(7 to 4222) | 70.35(0.33 to 198.64) | 1490(8 to 3935) | 96.70(0.53 to 257.69) | 1.06(0.97 to 1.14) |
| **Burkina Faso** | 954(5 to 3064) | 50.67(0.26 to 161.35) | 2458(10 to 7883) | 50.26(0.21 to 160.10) | 0.06(-0.22 to 0.33) |
| **Burundi** | 1886(11 to 5049) | 168.19(1.00 to 443.41) | 4494(29 to 11471) | 163.19(1.05 to 410.53) | -0.04(-0.08 to -0.01) |
| **Cabo Verde** | 40(0 to 126) | 59.37(0.27 to 184.80) | 125(0 to 388) | 84.31(0.32 to 261.82) | 1.21(0.95 to 1.46) |
| **Cambodia** | 741(3 to 2219) | 31.33(0.13 to 92.59) | 1982(11 to 5246) | 44.40(0.25 to 117.30) | 1.07(0.95 to 1.18) |
| **Cameroon** | 3026(18 to 7710) | 141.99(0.83 to 355.72) | 9402(52 to 24888) | 132.80(0.73 to 346.95) | -0.19(-0.27 to -0.12) |
| **Canada** | 7796(45 to 19705) | 102.99(0.59 to 261.25) | 6056(28 to 18625) | 72.16(0.34 to 223.79) | -1.05(-1.29 to -0.81) |
| **Central African Republic** | 1093(6 to 3015) | 173.87(0.97 to 478.93) | 2455(13 to 7203) | 185.47(1.00 to 540.83) | 0.34(0.05 to 0.63) |
| **Chad** | 1293(7 to 3564) | 107.01(0.56 to 293.70) | 4537(25 to 12376) | 134.49(0.74 to 362.77) | 0.88(0.71 to 1.05) |
| **Chile** | 6291(36 to 16054) | 174.66(1.00 to 444.62) | 9175(49 to 25823) | 190.50(1.03 to 540.14) | 0.34(-0.11 to 0.78) |
| **China** | 230895(807 to 583734) | 76.25(0.26 to 191.35) | 148268(322 to 380523) | 40.55(0.09 to 105.80) | -1.99(-2.20 to -1.79) |
| **Colombia** | 5103(30 to 13194) | 63.15(0.37 to 161.35) | 6046(35 to 15934) | 45.36(0.26 to 119.80) | -0.93(-1.43 to -0.42) |
| **Comoros** | 41(0 to 143) | 44.83(0.22 to 154.74) | 87(0 to 299) | 46.21(0.16 to 158.83) | 0.14(0.00 to 0.29) |
| **Congo** | 1117(6 to 3074) | 221.44(1.17 to 600.67) | 3272(16 to 9114) | 236.66(1.14 to 657.00) | 0.28(-0.13 to 0.69) |
| **Cook Islands** | 4(0 to 10) | 85.08(0.54 to 235.23) | 4(0 to 12) | 99.01(0.55 to 272.31) | 0.47(0.39 to 0.54) |
| **Costa Rica** | 679(3 to 1851) | 92.61(0.40 to 250.70) | 1632(9 to 4551) | 121.67(0.64 to 340.49) | 1.00(0.81 to 1.19) |
| **Coted'Ivoire** | 2692(14 to 7462) | 106.85(0.52 to 293.54) | 7228(38 to 18701) | 113.51(0.59 to 292.50) | 0.18(0.09 to 0.26) |
| **Croatia** | 807(4 to 2396) | 64.68(0.29 to 193.20) | 572(3 to 1742) | 58.98(0.27 to 181.75) | -0.21(-0.25 to -0.16) |
| **Cuba** | 3902(18 to 11809) | 130.92(0.60 to 394.03) | 2713(12 to 8528) | 101.62(0.48 to 320.42) | -0.83(-0.91 to -0.75) |
| **Cyprus** | 113(0 to 353) | 56.36(0.24 to 176.13) | 245(1 to 733) | 64.16(0.28 to 195.29) | 0.47(0.42 to 0.53) |
| **Czechia** | 2589(13 to 6676) | 97.22(0.51 to 252.52) | 2116(12 to 5824) | 84.21(0.49 to 235.91) | -0.43(-0.61 to -0.26) |
| **Democratic People's Republic of Korea** | 2658(11 to 7794) | 48.22(0.21 to 140.56) | 3115(17 to 9480) | 45.05(0.24 to 138.51) | -0.22(-0.26 to -0.17) |
| **Democratic Republic of the Congo** | 20154(122 to 48837) | 256.03(1.58 to 614.61) | 47364(268 to 113069) | 238.84(1.36 to 566.68) | -0.13(-0.38 to 0.12) |
| **Denmark** | 1884(9 to 4964) | 138.58(0.69 to 366.45) | 2053(10 to 5226) | 156.59(0.76 to 403.86) | 0.41(0.19 to 0.62) |
| **Djibouti** | 101(1 to 293) | 117.29(0.66 to 332.33) | 437(2 to 1254) | 135.42(0.77 to 388.50) | 0.53(0.35 to 0.70) |
| **Dominica** | 10(0 to 32) | 65.20(0.23 to 203.29) | 14(0 to 45) | 86.20(0.39 to 275.84) | 0.96(0.88 to 1.04) |
| **Dominican Republic** | 1504(8 to 4142) | 85.57(0.45 to 234.42) | 3332(17 to 9056) | 115.66(0.58 to 314.13) | 1.30(0.84 to 1.76) |
| **Ecuador** | 1800(9 to 5007) | 77.39(0.37 to 212.22) | 5919(38 to 15845) | 125.05(0.79 to 334.84) | 1.84(1.52 to 2.16) |
| **Egypt** | 9283(48 to 26968) | 75.44(0.39 to 217.50) | 24468(126 to 70900) | 96.10(0.50 to 277.71) | 0.86(0.58 to 1.14) |
| **El Salvador** | 936(4 to 2669) | 75.21(0.35 to 212.40) | 1842(9 to 5128) | 104.35(0.53 to 290.49) | 1.35(1.07 to 1.62) |
| **Equatorial Guinea** | 220(1 to 568) | 236.69(1.25 to 602.61) | 877(6 to 2339) | 251.76(1.60 to 668.88) | 0.37(0.12 to 0.62) |
| **Eritrea** | 946(5 to 2674) | 134.06(0.75 to 374.43) | 2217(11 to 6311) | 142.59(0.72 to 402.29) | 0.20(0.17 to 0.24) |
| **Estonia** | 434(2 to 1272) | 108.86(0.47 to 320.59) | 309(1 to 837) | 101.70(0.44 to 276.99) | -0.27(-0.34 to -0.19) |
| **Eswatini** | 253(1 to 647) | 145.85(0.88 to 365.77) | 624(3 to 1665) | 205.35(1.07 to 546.29) | 1.12(1.02 to 1.21) |
| **Ethiopia** | 17276(88 to 40547) | 171.32(0.92 to 397.80) | 25018(111 to 65175) | 100.47(0.44 to 258.27) | -1.68(-1.84 to -1.52) |
| **Fiji** | 141(1 to 380) | 73.46(0.39 to 196.33) | 204(1 to 539) | 89.34(0.56 to 235.96) | 0.59(0.55 to 0.63) |
| **Finland** | 2035(11 to 5352) | 151.19(0.79 to 400.70) | 2293(14 to 5838) | 196.68(1.19 to 506.21) | 0.97(0.34 to 1.61) |
| **France** | 15950(78 to 46013) | 108.27(0.53 to 314.23) | 23060(106 to 59021) | 155.71(0.72 to 400.58) | 1.43(0.93 to 1.93) |
| **Gabon** | 427(2 to 1099) | 214.79(1.07 to 543.65) | 1132(6 to 2905) | 244.37(1.29 to 622.65) | 0.45(0.35 to 0.56) |
| **Gambia** | 296(1 to 921) | 152.63(0.69 to 461.68) | 844(4 to 2407) | 156.51(0.70 to 437.75) | 0.10(-0.21 to 0.42) |
| **Georgia** | 239(1 to 752) | 17.16(0.08 to 54.24) | 143(1 to 487) | 16.29(0.06 to 55.61) | -0.16(-0.23 to -0.10) |
| **Germany** | 19932(113 to 48511) | 98.59(0.56 to 241.82) | 25701(141 to 67216) | 144.24(0.78 to 382.78) | 1.28(1.09 to 1.47) |
| **Ghana** | 2423(12 to 7431) | 77.10(0.38 to 232.16) | 7157(40 to 21456) | 82.96(0.47 to 246.76) | 0.33(0.21 to 0.45) |
| **Greece** | 3398(16 to 10130) | 132.73(0.61 to 396.68) | 3887(20 to 10778) | 170.38(0.83 to 480.26) | 0.87(0.11 to 1.64) |
| **Greenland** | 39(0 to 112) | 248.11(1.13 to 717.21) | 37(0 to 108) | 281.27(1.63 to 826.97) | 0.53(0.41 to 0.65) |
| **Grenada** | 12(0 to 37) | 66.58(0.28 to 208.22) | 22(0 to 68) | 85.09(0.45 to 262.72) | 0.84(0.76 to 0.91) |
| **Guam** | 26(0 to 69) | 75.65(0.38 to 198.31) | 34(0 to 93) | 94.10(0.45 to 256.19) | 0.77(0.68 to 0.87) |
| **Guatemala** | 1373(7 to 3816) | 88.34(0.44 to 242.89) | 3896(19 to 10823) | 94.25(0.46 to 260.74) | 0.32(-0.09 to 0.73) |
| **Guinea** | 1304(6 to 3986) | 102.38(0.47 to 309.61) | 3507(14 to 10059) | 116.95(0.47 to 330.40) | 0.48(0.34 to 0.63) |
| **Guinea-Bissau** | 156(1 to 483) | 75.74(0.34 to 232.58) | 407(2 to 1191) | 84.96(0.39 to 247.80) | 0.44(0.23 to 0.66) |
| **Guyana** | 205(1 to 611) | 108.57(0.51 to 320.30) | 328(2 to 1006) | 162.36(0.76 to 496.93) | 1.35(1.29 to 1.41) |
| **Haiti** | 1268(6 to 3651) | 87.06(0.42 to 247.81) | 4078(24 to 10954) | 115.23(0.67 to 308.85) | 0.99(0.94 to 1.04) |
| **Honduras** | 450(2 to 1292) | 49.40(0.27 to 140.19) | 2962(15 to 7930) | 110.17(0.55 to 293.94) | 2.80(2.67 to 2.93) |
| **Hungary** | 1530(6 to 4686) | 57.08(0.22 to 176.79) | 1485(8 to 4222) | 60.94(0.34 to 174.20) | 0.22(0.17 to 0.28) |
| **Iceland** | 13(0 to 44) | 19.34(0.07 to 68.15) | 15(0 to 56) | 18.36(0.06 to 68.17) | -0.12(-0.45 to 0.21) |
| **India** | 174595(492 to 456551) | 92.86(0.26 to 240.68) | 390689(1711 to 958576) | 104.92(0.46 to 256.86) | 0.53(0.34 to 0.72) |
| **Indonesia** | 12838(23 to 39308) | 28.57(0.06 to 86.94) | 29229(18 to 87028) | 37.96(0.02 to 113.35) | 0.95(0.89 to 1.02) |
| **Iran (Islamic Republic of)** | 18470(48 to 53564) | 160.10(0.45 to 460.02) | 46170(153 to 132608) | 186.68(0.61 to 539.83) | 0.49(0.28 to 0.70) |
| **Iraq** | 3550(19 to 10159) | 101.14(0.56 to 286.22) | 7556(34 to 22178) | 75.46(0.33 to 219.82) | -0.79(-2.07 to 0.51) |
| **Ireland** | 587(3 to 1710) | 67.57(0.32 to 196.87) | 1406(7 to 3929) | 117.89(0.56 to 330.03) | 1.93(1.50 to 2.35) |
| **Israel** | 1922(11 to 4961) | 159.87(0.89 to 411.42) | 4024(20 to 11239) | 180.29(0.90 to 504.33) | 0.49(0.36 to 0.63) |
| **Italy** | 20341(55 to 54088) | 140.40(0.37 to 373.91) | 24925(108 to 60907) | 196.42(0.81 to 485.82) | 1.25(1.11 to 1.38) |
| **Jamaica** | 256(1 to 841) | 48.65(0.18 to 156.98) | 826(4 to 2299) | 105.48(0.52 to 293.78) | 2.54(2.43 to 2.64) |
| **Japan** | 14555(33 to 39947) | 45.71(0.11 to 125.54) | 16225(22 to 45927) | 64.57(0.10 to 185.70) | 1.17(0.92 to 1.43) |
| **Jordan** | 675(3 to 1928) | 96.27(0.42 to 270.85) | 2364(11 to 6732) | 79.73(0.37 to 226.16) | -0.57(-0.66 to -0.48) |
| **Kazakhstan** | 1027(4 to 3538) | 25.72(0.09 to 88.18) | 1852(8 to 5416) | 36.51(0.16 to 107.51) | 1.20(1.08 to 1.32) |
| **Kenya** | 5251(14 to 13840) | 121.18(0.34 to 311.76) | 18040(61 to 43118) | 145.98(0.53 to 347.45) | 0.74(0.46 to 1.03) |
| **Kiribati** | 19(0 to 47) | 102.86(0.71 to 251.25) | 33(0 to 79) | 102.49(0.69 to 246.24) | -0.04(-0.11 to 0.04) |
| **Kuwait** | 509(3 to 1491) | 126.40(0.69 to 367.87) | 2041(12 to 5881) | 124.57(0.71 to 365.34) | 0.02(-0.33 to 0.38) |
| **Kyrgyzstan** | 600(3 to 1773) | 63.66(0.38 to 185.03) | 1447(8 to 3936) | 83.59(0.47 to 226.80) | 1.01(0.85 to 1.16) |
| **Lao People's Democratic Republic** | 329(1 to 1048) | 36.26(0.14 to 114.07) | 658(2 to 2114) | 33.42(0.13 to 107.02) | -0.32(-0.76 to 0.12) |
| **Latvia** | 930(5 to 2353) | 136.42(0.77 to 346.65) | 716(4 to 1806) | 166.72(0.97 to 424.85) | 0.51(0.22 to 0.81) |
| **Lebanon** | 906(4 to 2647) | 125.93(0.51 to 365.24) | 3173(16 to 9251) | 202.33(0.99 to 596.12) | 1.54(1.48 to 1.60) |
| **Lesotho** | 784(5 to 2031) | 222.03(1.34 to 569.64) | 1315(7 to 3496) | 277.37(1.60 to 730.63) | 0.69(0.59 to 0.80) |
| **Liberia** | 777(4 to 2003) | 152.71(0.77 to 390.80) | 2191(11 to 5997) | 168.07(0.83 to 456.77) | 0.40(0.36 to 0.44) |
| **Libya** | 1026(5 to 3055) | 131.32(0.71 to 380.19) | 3050(18 to 8761) | 148.35(0.89 to 427.54) | 0.50(0.24 to 0.76) |
| **Lithuania** | 1023(5 to 2920) | 108.11(0.48 to 308.57) | 911(4 to 2345) | 146.40(0.61 to 378.86) | 0.99(0.95 to 1.03) |
| **Luxembourg** | 116(1 to 328) | 113.63(0.61 to 323.08) | 178(1 to 459) | 108.84(0.58 to 283.76) | -0.07(-0.46 to 0.32) |
| **Madagascar** | 3073(16 to 8438) | 129.22(0.69 to 348.01) | 9396(44 to 26972) | 142.40(0.66 to 401.80) | 0.39(0.26 to 0.51) |
| **Malawi** | 1982(9 to 5260) | 97.23(0.47 to 253.25) | 5309(27 to 14349) | 118.70(0.61 to 319.05) | 0.64(0.59 to 0.69) |
| **Malaysia** | 1799(7 to 5680) | 42.65(0.16 to 133.83) | 4379(19 to 13304) | 51.76(0.23 to 157.44) | 0.47(-0.13 to 1.07) |
| **Maldives** | 21(0 to 72) | 49.81(0.21 to 165.67) | 47(0 to 134) | 39.31(0.19 to 112.77) | -0.72(-0.78 to -0.66) |
| **Mali** | 1358(6 to 3825) | 75.98(0.36 to 211.98) | 4819(24 to 12671) | 99.49(0.50 to 256.55) | 0.84(0.69 to 0.99) |
| **Malta** | 63(0 to 196) | 63.84(0.29 to 201.67) | 72(0 to 206) | 72.48(0.35 to 208.41) | 0.61(0.35 to 0.87) |
| **Marshall Islands** | 7(0 to 18) | 70.65(0.37 to 187.05) | 11(0 to 30) | 77.71(0.46 to 205.76) | 0.29(0.20 to 0.39) |
| **Mauritania** | 309(1 to 923) | 73.48(0.36 to 215.86) | 754(4 to 2291) | 77.93(0.43 to 234.87) | 0.24(0.17 to 0.32) |
| **Mauritius** | 220(1 to 698) | 74.20(0.30 to 235.39) | 256(1 to 795) | 78.21(0.36 to 243.82) | 0.22(0.16 to 0.27) |
| **Mexico** | 20028(98 to 48694) | 101.42(0.50 to 244.00) | 51766(179 to 136887) | 146.01(0.50 to 386.73) | 1.46(1.28 to 1.63) |
| **Micronesia (Federated States of)** | 16(0 to 45) | 74.25(0.40 to 199.36) | 20(0 to 56) | 77.85(0.43 to 217.53) | 0.12(0.05 to 0.19) |
| **Monaco** | 8(0 to 24) | 104.77(0.50 to 326.27) | 10(0 to 32) | 133.74(0.68 to 424.46) | 0.85(0.80 to 0.89) |
| **Mongolia** | 492(2 to 1393) | 106.62(0.48 to 298.89) | 1000(6 to 2504) | 112.64(0.68 to 283.36) | 0.13(0.06 to 0.20) |
| **Montenegro** | 89(0 to 259) | 57.01(0.28 to 166.35) | 121(1 to 364) | 79.57(0.40 to 240.89) | 1.17(1.09 to 1.25) |
| **Morocco** | 9159(44 to 26400) | 157.84(0.76 to 450.16) | 17847(75 to 51993) | 181.85(0.77 to 530.65) | 0.57(0.20 to 0.94) |
| **Mozambique** | 3651(19 to 9345) | 125.77(0.66 to 320.31) | 8199(42 to 23533) | 119.29(0.63 to 340.53) | -0.17(-0.24 to -0.10) |
| **Myanmar** | 2827(15 to 7644) | 29.60(0.16 to 78.47) | 4014(17 to 11965) | 26.62(0.11 to 79.28) | -0.37(-0.86 to 0.13) |
| **Namibia** | 251(1 to 680) | 84.34(0.43 to 225.85) | 477(2 to 1455) | 74.66(0.30 to 226.09) | -0.45(-0.89 to -0.02) |
| **Nauru** | 2(0 to 5) | 84.22(0.47 to 230.27) | 3(0 to 7) | 97.60(0.47 to 267.08) | 0.44(0.35 to 0.53) |
| **Nepal** | 4897(29 to 13009) | 116.01(0.67 to 307.09) | 12728(70 to 34234) | 147.17(0.82 to 394.34) | 0.91(0.84 to 0.97) |
| **Netherlands** | 5026(30 to 12106) | 123.11(0.73 to 297.38) | 5016(28 to 13631) | 132.49(0.73 to 360.20) | 0.32(-0.08 to 0.72) |
| **New Zealand** | 1352(7 to 3452) | 148.86(0.73 to 380.20) | 1687(9 to 4380) | 140.88(0.72 to 369.21) | -0.16(-0.37 to 0.06) |
| **Nicaragua** | 583(3 to 1639) | 73.08(0.36 to 203.48) | 1624(10 to 4471) | 90.30(0.53 to 248.14) | 0.78(0.38 to 1.19) |
| **Niger** | 1539(7 to 4569) | 100.61(0.43 to 295.36) | 4380(19 to 13205) | 98.55(0.42 to 291.62) | -0.08(-0.14 to -0.02) |
| **Nigeria** | 14905(48 to 38859) | 82.27(0.26 to 211.70) | 27811(123 to 70392) | 54.53(0.25 to 136.89) | -1.28(-1.57 to -0.99) |
| **Niue** | 0(0 to 1) | 84.15(0.55 to 231.47) | 0(0 to 1) | 97.09(0.50 to 258.82) | 0.43(0.34 to 0.53) |
| **North Macedonia** | 261(1 to 795) | 51.15(0.24 to 155.89) | 409(2 to 1278) | 71.84(0.35 to 225.70) | 1.21(1.09 to 1.32) |
| **Northern Mariana Islands** | 8(0 to 21) | 57.28(0.35 to 154.02) | 9(0 to 25) | 77.60(0.37 to 216.67) | 0.98(0.90 to 1.06) |
| **Norway** | 1327(6 to 3289) | 123.41(0.51 to 306.39) | 1586(5 to 4224) | 128.00(0.39 to 342.57) | 0.15(-0.04 to 0.34) |
| **Oman** | 377(2 to 1087) | 120.06(0.54 to 341.52) | 1553(7 to 4715) | 145.90(0.67 to 441.31) | 0.77(0.68 to 0.85) |
| **Pakistan** | 17308(59 to 47994) | 83.82(0.29 to 230.76) | 46591(208 to 125199) | 81.43(0.36 to 218.14) | 0.11(-0.25 to 0.47) |
| **Palau** | 3(0 to 10) | 84.90(0.50 to 236.64) | 4(0 to 11) | 99.60(0.51 to 283.56) | 0.48(0.41 to 0.56) |
| **Palestine** | 1180(7 to 3035) | 291.55(1.78 to 736.58) | 3102(18 to 8264) | 248.57(1.51 to 657.65) | -0.43(-0.70 to -0.16) |
| **Panama** | 351(2 to 1076) | 61.64(0.30 to 188.42) | 812(4 to 2447) | 75.91(0.41 to 228.62) | 0.88(0.41 to 1.36) |
| **Papua New Guinea** | 856(5 to 2231) | 93.01(0.51 to 237.20) | 2482(16 to 6174) | 95.18(0.62 to 236.64) | 0.03(-0.10 to 0.16) |
| **Paraguay** | 417(2 to 1265) | 48.05(0.22 to 145.17) | 1691(9 to 4795) | 89.90(0.45 to 255.82) | 2.08(2.03 to 2.14) |
| **Peru** | 3506(20 to 8797) | 71.63(0.42 to 177.66) | 6862(38 to 17457) | 70.53(0.39 to 179.54) | 0.09(-0.65 to 0.82) |
| **Philippines** | 4049(9 to 12190) | 28.10(0.06 to 84.19) | 8800(24 to 23635) | 30.68(0.09 to 82.18) | 0.38(0.21 to 0.56) |
| **Poland** | 2233(6 to 6488) | 23.12(0.07 to 66.96) | 2691(9 to 7397) | 27.78(0.09 to 77.31) | 0.67(0.52 to 0.81) |
| **Portugal** | 3859(18 to 11331) | 152.46(0.70 to 448.00) | 4340(19 to 11949) | 176.18(0.81 to 489.50) | 0.57(-0.27 to 1.42) |
| **Puerto Rico** | 444(2 to 1228) | 46.50(0.25 to 128.67) | 502(3 to 1437) | 64.92(0.36 to 187.50) | 1.23(0.97 to 1.49) |
| **Qatar** | 115(1 to 319) | 143.82(0.72 to 397.40) | 890(4 to 2576) | 144.33(0.73 to 420.99) | 0.08(-0.21 to 0.37) |
| **Republic of Korea** | 5491(31 to 16896) | 43.46(0.25 to 132.93) | 5891(32 to 16929) | 48.68(0.24 to 141.60) | 0.46(0.35 to 0.57) |
| **Republic of Moldova** | 649(3 to 1771) | 57.71(0.28 to 157.48) | 733(3 to 2077) | 72.59(0.32 to 206.54) | 0.81(0.73 to 0.89) |
| **Romania** | 2612(15 to 7267) | 46.16(0.26 to 128.83) | 3423(18 to 9218) | 78.93(0.43 to 214.70) | 1.93(1.76 to 2.10) |
| **Russian Federation** | 27501(87 to 72648) | 71.33(0.23 to 189.86) | 23662(38 to 66290) | 62.62(0.12 to 177.55) | -0.40(-0.60 to -0.21) |
| **Rwanda** | 2507(14 to 6611) | 181.94(1.04 to 469.45) | 5385(30 to 14088) | 167.10(0.93 to 432.84) | -0.29(-0.40 to -0.18) |
| **Saint Kitts and Nevis** | 8(0 to 26) | 88.40(0.46 to 280.87) | 18(0 to 54) | 108.10(0.47 to 333.27) | 0.74(0.66 to 0.83) |
| **Saint Lucia** | 21(0 to 67) | 66.88(0.30 to 212.73) | 44(0 to 140) | 91.83(0.52 to 293.08) | 1.04(0.99 to 1.08) |
| **Saint Vincent and the Grenadines** | 15(0 to 47) | 65.98(0.28 to 199.37) | 25(0 to 76) | 86.69(0.39 to 267.87) | 0.91(0.86 to 0.97) |
| **Samoa** | 17(0 to 48) | 51.46(0.28 to 139.71) | 24(0 to 71) | 51.55(0.27 to 151.22) | -0.04(-0.11 to 0.04) |
| **San Marino** | 7(0 to 20) | 104.00(0.54 to 323.04) | 10(0 to 31) | 137.53(0.79 to 423.52) | 1.01(0.66 to 1.35) |
| **Sao Tome and Principe** | 18(0 to 55) | 80.76(0.45 to 237.59) | 47(0 to 140) | 89.25(0.39 to 264.37) | 0.39(0.24 to 0.54) |
| **Saudi Arabia** | 3544(17 to 10572) | 120.65(0.61 to 351.88) | 15124(91 to 44156) | 139.98(0.84 to 411.38) | 0.72(0.34 to 1.11) |
| **Senegal** | 881(4 to 2874) | 58.67(0.25 to 190.64) | 2295(11 to 6313) | 64.14(0.31 to 174.67) | 0.34(0.18 to 0.50) |
| **Serbia** | 1125(5 to 3370) | 46.65(0.19 to 140.10) | 1145(5 to 3647) | 51.84(0.22 to 166.06) | 0.40(0.29 to 0.51) |
| **Seychelles** | 5(0 to 16) | 30.28(0.13 to 95.99) | 10(0 to 31) | 39.96(0.18 to 126.42) | 0.88(0.81 to 0.95) |
| **Sierra Leone** | 1258(7 to 3172) | 137.68(0.81 to 344.35) | 3204(18 to 8295) | 156.53(0.89 to 404.09) | 0.49(0.41 to 0.57) |
| **Singapore** | 804(4 to 2345) | 82.99(0.45 to 242.38) | 784(4 to 2431) | 51.36(0.24 to 163.48) | -1.53(-1.85 to -1.20) |
| **Slovakia** | 814(4 to 2354) | 60.13(0.30 to 174.01) | 1067(5 to 2901) | 77.31(0.39 to 213.43) | 0.77(0.57 to 0.97) |
| **Slovenia** | 354(2 to 1036) | 68.92(0.30 to 202.39) | 283(2 to 779) | 61.68(0.34 to 171.61) | -0.25(-0.40 to -0.11) |
| **Solomon Islands** | 59(0 to 154) | 87.34(0.56 to 221.68) | 153(1 to 404) | 92.20(0.54 to 241.58) | 0.15(0.11 to 0.19) |
| **Somalia** | 2123(12 to 5659) | 136.63(0.81 to 359.97) | 7422(35 to 20712) | 172.05(0.85 to 474.05) | 0.75(0.68 to 0.81) |
| **South Africa** | 3497(9 to 11004) | 39.93(0.09 to 125.28) | 8482(28 to 25257) | 53.58(0.18 to 159.64) | 1.06(0.70 to 1.42) |
| **South Sudan** | 1457(8 to 4029) | 129.81(0.71 to 353.65) | 2976(19 to 8359) | 142.11(0.89 to 393.90) | 0.40(0.22 to 0.59) |
| **Spain** | 7037(28 to 20304) | 73.39(0.30 to 211.65) | 12531(57 to 34266) | 118.86(0.52 to 326.32) | 1.89(1.53 to 2.24) |
| **Sri Lanka** | 2292(13 to 6307) | 51.25(0.29 to 140.53) | 2175(11 to 6084) | 37.62(0.19 to 105.61) | -1.02(-1.23 to -0.82) |
| **Sudan** | 7266(36 to 19882) | 168.76(0.84 to 454.38) | 20458(110 to 52298) | 192.14(1.04 to 484.04) | 0.42(0.14 to 0.71) |
| **Suriname** | 99(1 to 295) | 107.58(0.57 to 319.14) | 226(1 to 705) | 154.10(0.73 to 481.97) | 1.17(1.09 to 1.26) |
| **Sweden** | 2590(12 to 7581) | 121.24(0.55 to 355.76) | 4690(21 to 11817) | 204.28(0.94 to 518.65) | 1.82(1.40 to 2.24) |
| **Switzerland** | 1251(6 to 3745) | 67.26(0.35 to 202.77) | 1524(6 to 4912) | 70.92(0.29 to 232.09) | 0.17(0.03 to 0.31) |
| **Syrian Arab Republic** | 2883(13 to 8793) | 119.37(0.58 to 355.86) | 5169(26 to 15427) | 141.09(0.69 to 421.61) | 0.66(0.54 to 0.77) |
| **Taiwan (Province of China)** | 1770(8 to 5323) | 32.70(0.14 to 98.36) | 2612(14 to 7767) | 41.56(0.21 to 124.72) | 0.78(0.70 to 0.85) |
| **Tajikistan** | 488(2 to 1414) | 45.14(0.23 to 130.31) | 1732(9 to 4673) | 68.96(0.37 to 185.42) | 1.41(1.33 to 1.49) |
| **Thailand** | 7850(41 to 20110) | 50.82(0.27 to 129.43) | 9889(52 to 25945) | 56.06(0.30 to 148.95) | 0.28(0.24 to 0.33) |
| **Timor-Leste** | 169(1 to 418) | 91.33(0.53 to 224.99) | 184(1 to 516) | 57.32(0.35 to 156.38) | -1.53(-1.59 to -1.47) |
| **Togo** | 646(3 to 1982) | 86.65(0.35 to 261.93) | 1827(9 to 5030) | 89.51(0.46 to 245.18) | 0.13(-0.01 to 0.27) |
| **Tokelau** | 0(0 to 1) | 85.13(0.47 to 231.16) | 0(0 to 1) | 98.17(0.59 to 270.74) | 0.43(0.36 to 0.50) |
| **Tonga** | 13(0 to 37) | 60.14(0.35 to 167.27) | 16(0 to 46) | 65.91(0.30 to 181.82) | 0.27(0.20 to 0.35) |
| **Trinidad and Tobago** | 272(1 to 826) | 90.59(0.37 to 272.15) | 415(2 to 1288) | 116.72(0.52 to 365.71) | 0.77(0.68 to 0.87) |
| **Tunisia** | 2844(15 to 8230) | 150.84(0.79 to 430.64) | 6461(27 to 19356) | 199.93(0.82 to 603.78) | 0.92(0.85 to 0.99) |
| **Turkey** | 25656(147 to 61975) | 187.70(1.05 to 449.70) | 42169(266 to 110590) | 190.45(1.19 to 501.46) | 0.14(-0.14 to 0.42) |
| **Turkmenistan** | 269(1 to 957) | 33.54(0.12 to 117.37) | 475(2 to 1659) | 37.83(0.17 to 132.10) | 0.46(0.26 to 0.65) |
| **Tuvalu** | 2(0 to 6) | 83.89(0.40 to 233.77) | 3(0 to 8) | 96.82(0.50 to 271.29) | 0.46(0.37 to 0.55) |
| **Uganda** | 7140(46 to 18942) | 210.14(1.37 to 554.12) | 25383(151 to 61473) | 280.01(1.67 to 667.33) | 0.97(0.87 to 1.08) |
| **Ukraine** | 9639(42 to 27259) | 72.16(0.31 to 204.73) | 7052(31 to 22071) | 60.24(0.26 to 192.45) | -0.59(-0.76 to -0.42) |
| **United Arab Emirates** | 416(2 to 1176) | 124.81(0.61 to 354.51) | 2595(16 to 7585) | 131.60(0.84 to 396.71) | 0.28(0.23 to 0.33) |
| **United Kingdom** | 19236(12 to 51023) | 132.20(0.09 to 351.78) | 28686(51 to 71256) | 178.79(0.30 to 448.75) | 1.30(0.85 to 1.75) |
| **United Republic of Tanzania** | 6436(35 to 17044) | 123.60(0.67 to 320.07) | 22091(118 to 57410) | 161.06(0.87 to 414.07) | 0.91(0.86 to 0.96) |
| **United States of America** | 85045(304 to 200809) | 124.24(0.44 to 294.34) | 147576(610 to 386015) | 195.02(0.81 to 510.41) | 1.58(1.09 to 2.06) |
| **United States Virgin Islands** | 23(0 to 65) | 77.90(0.39 to 225.80) | 18(0 to 54) | 100.95(0.49 to 303.01) | 0.91(0.78 to 1.03) |
| **Uruguay** | 508(2 to 1564) | 67.73(0.29 to 208.53) | 934(5 to 2909) | 110.03(0.54 to 343.36) | 1.58(1.47 to 1.69) |
| **Uzbekistan** | 1571(7 to 5528) | 35.66(0.16 to 123.26) | 3556(14 to 12497) | 38.68(0.16 to 136.08) | 0.31(0.10 to 0.53) |
| **Vanuatu** | 29(0 to 78) | 87.60(0.52 to 227.62) | 71(0 to 184) | 92.51(0.55 to 239.24) | 0.17(0.07 to 0.28) |
| **Venezuela (Bolivarian Republic of)** | 3348(17 to 10130) | 74.78(0.38 to 223.69) | 6026(29 to 18574) | 84.15(0.40 to 261.38) | 0.41(0.23 to 0.58) |
| **Viet Nam** | 7352(37 to 19716) | 45.01(0.23 to 119.19) | 15769(100 to 41886) | 59.68(0.37 to 159.96) | 0.84(0.56 to 1.12) |
| **Yemen** | 4028(18 to 11730) | 159.87(0.71 to 463.33) | 12488(57 to 37741) | 159.44(0.72 to 477.61) | -0.13(-0.19 to -0.06) |
| **Zambia** | 2178(14 to 5501) | 135.01(0.88 to 334.41) | 5433(31 to 13725) | 124.04(0.72 to 308.53) | -0.30(-0.35 to -0.26) |
| **Zimbabwe** | 1606(9 to 4182) | 76.79(0.47 to 196.16) | 3226(18 to 8719) | 84.11(0.45 to 226.40) | 0.27(0.00 to 0.54) |

**Notes:** Rates are reported per 100,000 person-years. Data in parentheses are 95% uncertainty intervals for cases and age-standardized rates of mortality and DALYs, and 95% confidence intervals for AAPCs. **Abbreviations:** DALYs, disability-adjusted life-years; ASMR, age-standardized mortality rate; ASDR, age-standardized DALYs rate; AAPC, average annual percent change; SDI, socio-demographic index; UI, uncertainty interval; CI, confidence interval.

**Table S2. Age-standardised mortality rates and DALY rates in 1990 and 2021, and AAPC from 1990 to 2021 for the HIV/AIDS burden attributable to IPV among women of childbearing age , by SDI quintile, region level.and country.**

| **Location** | **Mortality** | | | | | **DALYs** | | | | |
| --- | --- | --- | --- | --- | --- | --- | --- | --- | --- | --- |
|  | **Mortality cases in 1990 (95% UI)** | **ASMR in 1990 (95% UI)** | **Mortality cases in 2021 (95% UI)** | **ASMR in 2021 (95% UI)** | **AAPC% (95%CI),**  **1990–2021** | **DALYs cases in 1990 (95% UI)** | **ASDR in 1990 (95% UI)** | **DALYs cases in 2021 (95% UI)** | **ASDR in 2021 (95% UI)** | **AAPC% (95%CI),**  **1990–2021** |
| **Global** | 15559(7460 to 26842) | 1.21(0.58 to 2.08) | 26604(13928 to 42481) | 1.33(0.70 to 2.12) | 0.29(-0.02 to 0.61) | 945228(465479 to 1604151) | 72.23(35.59 to 122.37) | 1585790(847922 to 2475828) | 79.65(42.58 to 124.37) | 0.30(0.00 to 0.60) |
| **Age** | | | | | | | | | | |
| **15-19 years** | 430(196 to 839) | 0.17(0.08 to 0.33) | 591(285 to 1019) | 0.19(0.09 to 0.34) | 0.43(0.10 to 0.77) | 34915(16548 to 65503) | 13.66(6.48 to 25.63) | 45891(22468 to 78014) | 15.11(7.40 to 25.69) | 0.30(0.03 to 0.58) |
| **20-24 years** | 1770(876 to 3164) | 0.73(0.36 to 1.30) | 1553(766 to 2574) | 0.53(0.26 to 0.88) | -1.07(-1.33 to -0.80) | 129875(66099 to 226782) | 53.20(27.07 to 92.89) | 113905(57037 to 186818) | 38.78(19.42 to 63.60) | -1.06(-1.31 to -0.81) |
| **25-29 years** | 3631(1667 to 6183) | 1.65(0.76 to 2.81) | 3287(1738 to 5104) | 1.13(0.60 to 1.75) | -1.26(-1.66 to -0.86) | 243883(115134 to 409319) | 110.81(52.31 to 185.97) | 227001(124340 to 346763) | 78.01(42.73 to 119.17) | -1.16(-1.53 to -0.79) |
| **30-34 years** | 3597(1757 to 6010) | 1.89(0.92 to 3.16) | 5271(2796 to 8170) | 1.76(0.94 to 2.73) | -0.31(-0.77 to 0.16) | 220480(110102 to 362267) | 115.98(57.92 to 190.56) | 335653(184489 to 511099) | 112.28(61.72 to 170.98) | -0.18(-0.63 to 0.27) |
| **35-39 years** | 2572(1261 to 4634) | 1.48(0.73 to 2.67) | 5534(2903 to 8859) | 1.99(1.04 to 3.19) | 0.97(0.56 to 1.38) | 144199(72623 to 253834) | 83.13(41.87 to 146.34) | 327580(174214 to 511406) | 117.92(62.71 to 184.09) | 1.15(0.75 to 1.54) |
| **35-39 years** | 1889(904 to 3234) | 1.35(0.64 to 2.31) | 5576(3005 to 8645) | 2.25(1.21 to 3.48) | 1.64(1.14 to 2.13) | 96024(47601 to 161951) | 68.48(33.95 to 115.49) | 300261(160994 to 458208) | 121.03(64.89 to 184.69) | 1.82(1.35 to 2.30) |
| **45-49 years** | 1668(799 to 2778) | 1.47(0.70 to 2.44) | 4792(2436 to 8110) | 2.03(1.03 to 3.44) | 1.06(0.72 to 1.41) | 75851(37372 to 124495) | 66.65(32.84 to 109.40) | 235499(124381 to 383520) | 99.94(52.78 to 162.75) | 1.31(0.99 to 1.64) |
| **SDI regions** | | | | | | | | | | |
| **High SDI** | 385(215 to 563) | 0.16(0.09 to 0.24) | 149(85 to 221) | 0.05(0.03 to 0.08) | -3.76(-4.51 to -3.01) | 22626(12568 to 33373) | 9.50(5.27 to 14.02) | 10437(5708 to 15890) | 3.83(2.09 to 5.84) | -3.00(-3.86 to -2.12) |
| **High-middle SDI** | 139(76 to 206) | 0.05(0.03 to 0.07) | 565(303 to 862) | 0.16(0.08 to 0.24) | 3.72(3.24 to 4.21) | 8195(4484 to 12204) | 2.96(1.62 to 4.40) | 32378(17149 to 49658) | 9.27(4.89 to 14.33) | 3.73(3.06 to 4.41) |
| **Middle SDI** | 701(401 to 1053) | 0.17(0.10 to 0.25) | 3995(2154 to 6154) | 0.61(0.33 to 0.94) | 4.22(3.59 to 4.84) | 42119(23929 to 63195) | 9.92(5.67 to 14.84) | 242701(130731 to 371583) | 37.15(19.96 to 57.05) | 4.33(3.74 to 4.93) |
| **Low-middle SDI** | 3771(1683 to 7085) | 1.47(0.65 to 2.76) | 10527(5412 to 17311) | 2.18(1.12 to 3.59) | 1.28(0.85 to 1.71) | 236962(108796 to 431549) | 89.89(41.14 to 163.94) | 612754(320808 to 996575) | 125.90(65.96 to 204.58) | 1.09(0.69 to 1.50) |
| **Low SDI** | 10549(4833 to 18548) | 10.53(4.84 to 18.45) | 11350(5655 to 18763) | 4.82(2.39 to 7.99) | -2.57(-3.23 to -1.91) | 634500(295419 to 1099817) | 614.95(287.12 to 1062.63) | 686384(354091 to 1097101) | 285.22(147.04 to 456.41) | -2.53(-3.18 to -1.88) |
| **Regions** | | | | | | | | | | |
| **Andean Latin America** | 11(6 to 16) | 0.14(0.08 to 0.20) | 54(30 to 82) | 0.31(0.17 to 0.47) | 2.72(2.18 to 3.26) | 628(348 to 934) | 7.67(4.29 to 11.29) | 3178(1769 to 4755) | 18.05(10.05 to 27.01) | 2.87(2.26 to 3.48) |
| **Australasia** | 1(0 to 1) | 0.01(0.01 to 0.02) | 1(0 to 1) | 0.01(0.00 to 0.01) | -1.27(-3.55 to 1.07) | 48(26 to 75) | 0.88(0.47 to 1.36) | 59(29 to 101) | 0.76(0.36 to 1.29) | -0.50(-2.72 to 1.78) |
| **Caribbean** | 189(75 to 376) | 2.19(0.87 to 4.34) | 170(75 to 310) | 1.39(0.61 to 2.53) | -1.36(-2.24 to -0.46) | 11082(4384 to 21811) | 125.67(50.07 to 246.94) | 10214(4527 to 18321) | 83.74(37.04 to 150.40) | -1.26(-2.26 to -0.25) |
| **Central Asia** | 4(2 to 7) | 0.03(0.01 to 0.04) | 15(8 to 23) | 0.06(0.03 to 0.09) | 2.47(1.66 to 3.29) | 246(128 to 396) | 1.53(0.80 to 2.45) | 876(468 to 1363) | 3.40(1.81 to 5.29) | 2.60(1.83 to 3.37) |
| **Central Europe** | 4(2 to 7) | 0.01(0.01 to 0.02) | 5(3 to 8) | 0.02(0.01 to 0.03) | 1.15(-0.15 to 2.47) | 249(134 to 392) | 0.79(0.42 to 1.25) | 321(173 to 501) | 1.20(0.64 to 1.89) | 1.56(0.39 to 2.75) |
| **Central Latin America** | 87(50 to 129) | 0.24(0.14 to 0.36) | 191(108 to 285) | 0.28(0.16 to 0.41) | 0.42(-0.03 to 0.88) | 4895(2817 to 7236) | 13.32(7.71 to 19.61) | 10838(6079 to 16251) | 15.69(8.80 to 23.54) | 0.56(0.02 to 1.10) |
| **Central Sub-Saharan Africa** | 1697(771 to 3159) | 15.73(7.21 to 29.16) | 2250(1116 to 3845) | 8.19(4.10 to 13.87) | -2.16(-2.70 to -1.62) | 101718(47101 to 187328) | 912.84(426.10 to 1672.35) | 131083(65780 to 220910) | 466.76(236.53 to 778.60) | -2.21(-2.75 to -1.68) |
| **East Asia** | 31(0 to 74) | 0.01(0.00 to 0.02) | 281(132 to 484) | 0.07(0.03 to 0.12) | 6.38(5.32 to 7.44) | 1834(13 to 4217) | 0.61(0.00 to 1.39) | 14988(7002 to 26078) | 3.84(1.78 to 6.73) | 6.12(5.13 to 7.12) |
| **Eastern Europe** | 60(32 to 93) | 0.10(0.05 to 0.16) | 409(210 to 638) | 0.70(0.36 to 1.11) | 6.51(5.57 to 7.45) | 3388(1764 to 5246) | 5.73(2.97 to 8.91) | 23467(11806 to 36990) | 41.13(20.41 to 65.94) | 6.62(5.71 to 7.54) |
| **Eastern Sub-Saharan Africa** | 9427(4281 to 16839) | 25.10(11.38 to 44.57) | 11252(5473 to 18997) | 12.47(6.04 to 21.17) | -2.21(-2.84 to -1.58) | 571140(264045 to 1005099) | 1464.34(677.02 to 2563.21) | 684677(341302 to 1120463) | 739.98(368.27 to 1213.19) | -2.20(-2.81 to -1.58) |
| **High-income Asia Pacific** | 1(0 to 1) | 0.00(0.00 to 0.00) | 1(1 to 1) | 0.00(0.00 to 0.00) | 2.08(1.51 to 2.66) | 37(16 to 64) | 0.08(0.03 to 0.14) | 82(41 to 136) | 0.19(0.09 to 0.32) | 2.81(2.40 to 3.23) |
| **High-income North America** | 312(174 to 466) | 0.39(0.22 to 0.58) | 81(44 to 122) | 0.09(0.05 to 0.13) | -4.74(-5.30 to -4.18) | 18361(10178 to 27489) | 22.90(12.68 to 34.30) | 6070(3189 to 9696) | 6.68(3.50 to 10.69) | -4.01(-4.53 to -3.49) |
| **North Africa and Middle East** | 23(10 to 53) | 0.03(0.02 to 0.08) | 444(183 to 995) | 0.27(0.11 to 0.61) | 6.88(6.44 to 7.32) | 1347(591 to 3040) | 1.94(0.86 to 4.30) | 24571(10241 to 54949) | 15.14(6.30 to 33.92) | 6.80(6.38 to 7.23) |
| **Oceania** | 1(1 to 2) | 0.07(0.04 to 0.11) | 43(17 to 87) | 1.33(0.52 to 2.69) | 9.67(6.94 to 12.46) | 57(31 to 88) | 4.13(2.25 to 6.27) | 2656(1174 to 5056) | 80.86(35.78 to 153.93) | 10.02(7.78 to 12.31) |
| **South Asia** | 3(1 to 9) | 0.00(0.00 to 0.00) | 992(478 to 1753) | 0.21(0.10 to 0.37) | 17.50(15.59 to 19.44) | 288(90 to 676) | 0.12(0.04 to 0.28) | 56273(27227 to 98735) | 11.78(5.72 to 20.61) | 15.82(14.68 to 16.97) |
| **Southeast Asia** | 288(151 to 451) | 0.25(0.13 to 0.39) | 481(250 to 767) | 0.25(0.13 to 0.40) | 0.43(-0.35 to 1.21) | 16822(8788 to 26381) | 14.33(7.53 to 22.35) | 28671(14831 to 45544) | 15.15(7.83 to 24.08) | 0.52(-0.21 to 1.26) |
| **Southern Latin America** | 10(5 to 18) | 0.09(0.04 to 0.15) | 34(17 to 54) | 0.18(0.09 to 0.29) | 2.92(2.56 to 3.28) | 609(281 to 1065) | 5.03(2.33 to 8.80) | 1989(1003 to 3201) | 10.83(5.45 to 17.49) | 2.68(1.75 to 3.63) |
| **Southern Sub-Saharan Africa** | 1343(527 to 2720) | 10.87(4.26 to 22.09) | 3721(1757 to 6264) | 17.32(8.16 to 29.23) | 1.34(0.68 to 2.00) | 86016(35670 to 168601) | 672.70(279.68 to 1322.06) | 231373(112826 to 382904) | 1069.86(521.67 to 1772.63) | 1.33(0.65 to 2.01) |
| **Tropical Latin America** | 145(77 to 220) | 0.38(0.21 to 0.58) | 253(138 to 391) | 0.38(0.21 to 0.60) | -0.11(-1.11 to 0.90) | 8761(4610 to 13408) | 22.64(12.00 to 34.41) | 14369(7754 to 22632) | 21.99(11.80 to 34.82) | -0.10(-1.07 to 0.87) |
| **Western Europe** | 109(59 to 163) | 0.11(0.06 to 0.17) | 38(22 to 55) | 0.04(0.02 to 0.05) | -3.63(-4.72 to -2.52) | 6645(3581 to 9929) | 6.76(3.64 to 10.10) | 2875(1626 to 4359) | 2.74(1.55 to 4.16) | -2.87(-3.65 to -2.08) |
| **Western Sub-Saharan Africa** | 1809(736 to 3509) | 4.69(1.91 to 9.04) | 5887(2971 to 9754) | 6.07(3.07 to 9.99) | 0.88(-0.01 to 1.78) | 111057(46090 to 211766) | 277.06(115.17 to 525.47) | 337158(173096 to 549400) | 338.88(174.75 to 548.67) | 0.70(-0.15 to 1.57) |
| **Countries** | | | | | | | | | | |
| **Afghanistan** | 0(0 to 1) | 0.02(0.00 to 0.06) | 3(1 to 8) | 0.05(0.01 to 0.13) | 2.77(2.27 to 3.28) | 22(3 to 55) | 1.30(0.18 to 3.23) | 184(44 to 439) | 3.11(0.75 to 7.37) | 2.84(2.36 to 3.33) |
| **Albania** | 0(0 to 0) | 0.00(0.00 to 0.00) | 0(0 to 0) | 0.00(0.00 to 0.00) | 0.23(-0.20 to 0.67) | 1(1 to 2) | 0.16(0.07 to 0.27) | 1(0 to 2) | 0.16(0.07 to 0.29) | 0.18(-0.21 to 0.56) |
| **Algeria** | 1(0 to 1) | 0.01(0.01 to 0.03) | 6(2 to 15) | 0.05(0.02 to 0.12) | 4.33(3.78 to 4.88) | 37(13 to 78) | 0.77(0.29 to 1.59) | 397(142 to 883) | 3.33(1.19 to 7.43) | 4.79(4.27 to 5.31) |
| **American Samoa** | 0(0 to 0) | 0.13(0.06 to 0.21) | 0(0 to 0) | 0.44(0.24 to 0.69) | 3.83(0.25 to 7.55) | 1(0 to 1) | 6.88(3.38 to 11.11) | 3(2 to 4) | 24.88(13.39 to 39.09) | 4.51(2.06 to 7.02) |
| **Andorra** | 0(0 to 0) | 0.01(0.00 to 0.02) | 0(0 to 0) | 0.00(0.00 to 0.01) | -3.04(-3.78 to -2.30) | 0(0 to 0) | 0.62(0.21 to 1.42) | 0(0 to 0) | 0.32(0.11 to 0.73) | -2.13(-2.79 to -1.47) |
| **Angola** | 12(3 to 30) | 0.58(0.17 to 1.46) | 1108(480 to 2176) | 17.10(7.49 to 33.10) | 11.46(9.63 to 13.33) | 751(211 to 1844) | 35.47(10.20 to 86.67) | 63519(27764 to 123848) | 960.01(424.94 to 1845.66) | 11.13(9.43 to 12.86) |
| **Antigua and Barbuda** | 0(0 to 0) | 0.65(0.25 to 1.22) | 0(0 to 0) | 0.34(0.16 to 0.57) | -1.51(-2.65 to -0.35) | 6(2 to 11) | 35.49(13.53 to 66.81) | 5(2 to 9) | 18.99(8.76 to 33.42) | -1.42(-2.47 to -0.36) |
| **Argentina** | 7(3 to 14) | 0.09(0.04 to 0.18) | 23(10 to 41) | 0.18(0.08 to 0.32) | 2.69(2.28 to 3.11) | 443(168 to 854) | 5.61(2.13 to 10.82) | 1336(598 to 2371) | 10.73(4.78 to 19.13) | 2.37(1.33 to 3.43) |
| **Armenia** | 0(0 to 0) | 0.00(0.00 to 0.01) | 0(0 to 0) | 0.01(0.00 to 0.01) | 2.65(1.91 to 3.39) | 1(0 to 4) | 0.17(0.00 to 0.49) | 4(2 to 8) | 0.51(0.22 to 0.96) | 3.36(2.62 to 4.10) |
| **Australia** | 1(0 to 1) | 0.01(0.01 to 0.02) | 1(0 to 1) | 0.01(0.00 to 0.01) | -0.86(-3.07 to 1.39) | 34(17 to 56) | 0.75(0.38 to 1.22) | 50(23 to 86) | 0.75(0.35 to 1.30) | 0.09(-2.24 to 2.47) |
| **Austria** | 1(0 to 1) | 0.03(0.01 to 0.06) | 0(0 to 0) | 0.01(0.01 to 0.02) | -2.93(-4.51 to -1.33) | 35(13 to 71) | 1.73(0.62 to 3.49) | 22(10 to 41) | 1.01(0.47 to 1.87) | -1.56(-3.21 to 0.13) |
| **Azerbaijan** | 0(0 to 0) | 0.02(0.01 to 0.03) | 0(0 to 1) | 0.01(0.01 to 0.02) | -1.24(-1.71 to -0.77) | 18(8 to 29) | 0.94(0.44 to 1.56) | 22(11 to 36) | 0.71(0.36 to 1.19) | -1.03(-1.52 to -0.55) |
| **Bahamas** | 1(1 to 2) | 2.04(0.82 to 3.69) | 2(1 to 3) | 1.74(0.78 to 3.03) | -0.68(-1.98 to 0.63) | 76(30 to 140) | 112.97(44.57 to 205.85) | 109(48 to 191) | 98.03(42.86 to 172.16) | -0.63(-1.87 to 0.62) |
| **Bahrain** | 0(0 to 0) | 0.03(0.01 to 0.04) | 0(0 to 1) | 0.09(0.04 to 0.15) | 4.54(0.45 to 8.80) | 2(1 to 3) | 1.43(0.61 to 2.46) | 16(8 to 28) | 4.80(2.29 to 8.14) | 5.67(3.19 to 8.21) |
| **Bangladesh** | 0(0 to 0) | 0.00(0.00 to 0.00) | 19(8 to 38) | 0.04(0.02 to 0.08) | 21.04(20.53 to 21.55) | 2(1 to 3) | 0.01(0.00 to 0.02) | 1102(466 to 2176) | 2.44(1.04 to 4.81) | 20.79(20.33 to 21.26) |
| **Barbados** | 0(0 to 1) | 0.61(0.24 to 1.11) | 0(0 to 1) | 0.36(0.16 to 0.66) | -1.41(-2.19 to -0.62) | 22(9 to 41) | 32.82(12.93 to 60.18) | 16(7 to 30) | 21.39(9.28 to 40.21) | -1.15(-1.94 to -0.36) |
| **Belarus** | 1(0 to 2) | 0.04(0.00 to 0.09) | 4(2 to 7) | 0.16(0.08 to 0.26) | 4.99(3.41 to 6.59) | 55(0 to 125) | 2.06(0.00 to 4.69) | 237(123 to 385) | 9.15(4.68 to 15.03) | 5.08(3.66 to 6.53) |
| **Belgium** | 2(1 to 4) | 0.09(0.04 to 0.15) | 1(1 to 2) | 0.04(0.02 to 0.06) | -2.90(-4.25 to -1.53) | 128(55 to 227) | 4.98(2.13 to 8.84) | 83(42 to 138) | 3.08(1.56 to 5.11) | -1.45(-2.49 to -0.39) |
| **Belize** | 0(0 to 0) | 0.56(0.20 to 1.14) | 1(0 to 1) | 0.60(0.26 to 1.09) | 1.01(0.29 to 1.73) | 11(4 to 22) | 30.56(10.77 to 61.96) | 39(17 to 73) | 34.41(14.79 to 63.21) | 1.12(0.41 to 1.82) |
| **Benin** | 1(0 to 3) | 0.10(0.01 to 0.33) | 49(18 to 101) | 1.87(0.69 to 3.79) | 9.60(7.38 to 11.87) | 77(15 to 232) | 7.26(1.40 to 21.73) | 2938(1151 to 5862) | 108.75(42.98 to 214.26) | 8.74(6.59 to 10.93) |
| **Bermuda** | 0(0 to 0) | 0.92(0.39 to 1.62) | 0(0 to 0) | 0.35(0.18 to 0.57) | -2.42(-3.32 to -1.51) | 9(4 to 16) | 48.93(20.46 to 86.20) | 3(2 to 5) | 19.44(9.66 to 31.62) | -2.33(-3.21 to -1.44) |
| **Bhutan** | 0(0 to 0) | 0.02(0.00 to 0.06) | 0(0 to 0) | 0.08(0.02 to 0.21) | 4.05(2.72 to 5.40) | 1(0 to 4) | 1.19(0.20 to 3.45) | 10(3 to 24) | 4.70(1.39 to 11.72) | 4.21(2.82 to 5.62) |
| **Bolivia (Plurinational State of)** | 0(0 to 1) | 0.02(0.00 to 0.07) | 10(3 to 23) | 0.32(0.09 to 0.77) | 10.00(9.20 to 10.80) | 14(1 to 60) | 1.00(0.10 to 4.23) | 555(178 to 1304) | 18.26(5.85 to 42.90) | 9.73(8.95 to 10.52) |
| **Bosnia and Herzegovina** | 0(0 to 0) | 0.01(0.00 to 0.02) | 0(0 to 0) | 0.01(0.01 to 0.02) | 0.96(0.62 to 1.30) | 6(3 to 12) | 0.54(0.22 to 1.00) | 5(2 to 9) | 0.69(0.30 to 1.23) | 0.95(0.60 to 1.30) |
| **Botswana** | 82(27 to 182) | 28.15(9.44 to 62.18) | 328(126 to 678) | 48.89(18.76 to 101.42) | 1.73(0.54 to 2.94) | 5350(1932 to 11368) | 1764.69(644.19 to 3734.58) | 19368(8018 to 38519) | 2858.81(1180.50 to 5712.31) | 1.51(0.47 to 2.57) |
| **Brazil** | 145(77 to 219) | 0.39(0.21 to 0.59) | 249(136 to 385) | 0.39(0.21 to 0.60) | -0.13(-1.13 to 0.89) | 8733(4590 to 13367) | 23.10(12.23 to 35.12) | 14171(7632 to 22344) | 22.31(11.94 to 35.39) | -0.12(-1.08 to 0.85) |
| **Brunei Darussalam** | 0(0 to 0) | 0.01(0.00 to 0.01) | 0(0 to 0) | 0.02(0.01 to 0.04) | 3.55(2.70 to 4.42) | 0(0 to 1) | 0.50(0.21 to 0.92) | 2(1 to 3) | 1.47(0.63 to 2.62) | 3.54(2.80 to 4.28) |
| **Bulgaria** | 1(0 to 1) | 0.03(0.00 to 0.06) | 1(0 to 1) | 0.04(0.02 to 0.06) | 0.97(-0.67 to 2.64) | 37(0 to 70) | 1.79(0.00 to 3.42) | 37(19 to 60) | 2.44(1.21 to 4.01) | 1.15(-0.37 to 2.69) |
| **Burkina Faso** | 240(70 to 577) | 13.12(3.84 to 31.27) | 30(10 to 64) | 0.66(0.22 to 1.44) | -9.18(-9.97 to -8.38) | 14049(4113 to 33563) | 747.20(221.36 to 1768.78) | 1774(614 to 3772) | 38.61(13.54 to 81.34) | -9.19(-9.72 to -8.65) |
| **Burundi** | 287(86 to 713) | 25.67(7.81 to 63.90) | 63(22 to 138) | 2.55(0.88 to 5.57) | -7.33(-8.60 to -6.04) | 17467(5332 to 42320) | 1504.73(469.23 to 3648.78) | 3825(1411 to 8041) | 149.01(55.69 to 310.99) | -7.36(-8.63 to -6.07) |
| **Cabo Verde** | 1(0 to 2) | 0.92(0.21 to 2.62) | 1(0 to 3) | 0.55(0.11 to 1.92) | -1.77(-2.52 to -1.02) | 39(9 to 108) | 54.72(13.18 to 152.14) | 49(10 to 167) | 33.12(7.12 to 113.37) | -1.72(-2.46 to -0.98) |
| **Cambodia** | 0(0 to 0) | 0.00(0.00 to 0.00) | 12(3 to 29) | 0.29(0.07 to 0.67) | 32.52(27.72 to 37.50) | 0(0 to 0) | 0.01(0.00 to 0.02) | 739(233 to 1622) | 17.03(5.34 to 37.34) | 26.74(22.47 to 31.16) |
| **Cameroon** | 92(35 to 192) | 4.32(1.63 to 9.01) | 986(446 to 1774) | 15.56(7.09 to 27.89) | 4.15(3.02 to 5.29) | 5828(2286 to 11868) | 263.55(103.96 to 535.93) | 56411(26276 to 99721) | 865.67(406.63 to 1521.14) | 3.84(2.80 to 4.90) |
| **Canada** | 5(3 to 7) | 0.06(0.03 to 0.09) | 1(1 to 2) | 0.01(0.01 to 0.02) | -4.65(-5.84 to -3.45) | 290(151 to 453) | 3.67(1.90 to 5.75) | 134(59 to 261) | 1.45(0.63 to 2.82) | -2.95(-3.80 to -2.09) |
| **Central African Republic** | 111(33 to 262) | 17.62(5.01 to 41.97) | 240(100 to 468) | 20.48(8.73 to 39.52) | 0.24(-0.94 to 1.45) | 7314(2371 to 16563) | 1126.50(357.40 to 2567.20) | 14005(5998 to 27006) | 1172.15(510.74 to 2227.80) | 0.03(-1.12 to 1.19) |
| **Chad** | 28(9 to 63) | 2.35(0.78 to 5.25) | 104(37 to 212) | 3.38(1.21 to 6.88) | 1.04(-0.20 to 2.31) | 1775(605 to 3909) | 144.16(49.62 to 316.03) | 6294(2421 to 12416) | 199.54(76.98 to 389.04) | 0.91(-0.25 to 2.09) |
| **Chile** | 2(1 to 4) | 0.06(0.03 to 0.10) | 9(4 to 13) | 0.17(0.09 to 0.26) | 3.41(2.88 to 3.94) | 132(66 to 215) | 3.78(1.90 to 6.12) | 530(259 to 874) | 10.52(5.12 to 17.40) | 3.66(3.18 to 4.15) |
| **China** | 31(0 to 73) | 0.01(0.00 to 0.03) | 273(128 to 469) | 0.07(0.03 to 0.12) | 6.23(5.16 to 7.32) | 1824(7 to 4199) | 0.62(0.00 to 1.43) | 14517(6773 to 25228) | 3.85(1.78 to 6.75) | 6.03(5.02 to 7.05) |
| **Colombia** | 13(7 to 19) | 0.17(0.09 to 0.25) | 44(25 to 65) | 0.33(0.18 to 0.49) | 2.11(1.03 to 3.21) | 704(388 to 1068) | 9.09(5.06 to 13.68) | 2500(1384 to 3752) | 18.72(10.37 to 28.11) | 2.33(1.30 to 3.38) |
| **Comoros** | 0(0 to 0) | 0.00(0.00 to 0.01) | 0(0 to 0) | 0.01(0.00 to 0.02) | 3.72(1.99 to 5.47) | 0(0 to 1) | 0.17(0.01 to 0.65) | 1(0 to 2) | 0.54(0.16 to 1.32) | 3.76(2.05 to 5.50) |
| **Congo** | 113(35 to 262) | 23.13(7.33 to 53.36) | 261(107 to 518) | 19.55(8.08 to 38.47) | -0.58(-1.67 to 0.53) | 6860(2141 to 15727) | 1355.22(432.56 to 3077.83) | 14789(5989 to 29397) | 1096.28(448.80 to 2163.14) | -0.71(-1.73 to 0.33) |
| **Cook Islands** | 0(0 to 0) | 0.01(0.00 to 0.03) | 0(0 to 0) | 0.36(0.10 to 0.79) | 11.23(10.58 to 11.89) | 0(0 to 0) | 0.74(0.12 to 1.81) | 1(0 to 2) | 20.33(6.34 to 44.34) | 11.19(10.57 to 11.82) |
| **Costa Rica** | 1(1 to 2) | 0.21(0.10 to 0.36) | 3(2 to 5) | 0.22(0.11 to 0.36) | 0.07(-0.56 to 0.71) | 79(38 to 134) | 11.36(5.48 to 19.00) | 168(86 to 276) | 12.29(6.27 to 20.28) | 0.22(-0.39 to 0.83) |
| **Coted'Ivoire** | 903(294 to 1985) | 36.92(12.08 to 80.53) | 642(260 to 1187) | 11.03(4.44 to 20.59) | -4.03(-4.88 to -3.17) | 55282(18298 to 120029) | 2162.61(719.37 to 4660.29) | 37913(15995 to 68179) | 633.87(266.66 to 1148.36) | -4.06(-4.81 to -3.32) |
| **Croatia** | 0(0 to 0) | 0.01(0.00 to 0.02) | 0(0 to 0) | 0.01(0.00 to 0.01) | -0.81(-2.12 to 0.51) | 6(2 to 12) | 0.49(0.16 to 0.97) | 4(2 to 7) | 0.42(0.19 to 0.76) | -0.48(-1.71 to 0.77) |
| **Cuba** | 2(1 to 3) | 0.06(0.03 to 0.11) | 2(1 to 3) | 0.07(0.03 to 0.12) | 0.78(-0.82 to 2.41) | 95(39 to 178) | 3.30(1.36 to 6.15) | 118(53 to 210) | 4.38(1.96 to 7.84) | 1.43(0.06 to 2.82) |
| **Cyprus** | 0(0 to 0) | 0.01(0.01 to 0.02) | 0(0 to 0) | 0.01(0.01 to 0.02) | 0.54(-0.10 to 1.18) | 1(1 to 3) | 0.68(0.28 to 1.28) | 3(2 to 6) | 0.82(0.41 to 1.38) | 0.63(0.02 to 1.25) |
| **Czechia** | 0(0 to 0) | 0.01(0.00 to 0.01) | 0(0 to 0) | 0.01(0.00 to 0.01) | -0.66(-2.66 to 1.38) | 11(6 to 19) | 0.44(0.22 to 0.72) | 11(5 to 18) | 0.44(0.21 to 0.75) | 0.11(-1.54 to 1.79) |
| **Democratic People's Republic of Korea** | 0(0 to 0) | 0.00(0.00 to 0.01) | 7(1 to 31) | 0.10(0.01 to 0.44) | 15.18(13.56 to 16.83) | 5(0 to 16) | 0.09(0.00 to 0.30) | 429(64 to 1732) | 6.19(0.91 to 25.04) | 14.94(13.58 to 16.32) |
| **Democratic Republic of the Congo** | 1447(637 to 2793) | 19.66(8.76 to 37.62) | 412(166 to 860) | 2.30(0.93 to 4.81) | -6.95(-7.76 to -6.13) | 85934(38430 to 163353) | 1130.29(512.02 to 2132.51) | 25301(10941 to 50267) | 137.96(60.36 to 272.38) | -6.83(-7.64 to -6.00) |
| **Denmark** | 1(0 to 1) | 0.04(0.02 to 0.07) | 0(0 to 0) | 0.02(0.01 to 0.03) | -2.13(-3.16 to -1.10) | 33(17 to 53) | 2.43(1.22 to 3.91) | 18(10 to 27) | 1.32(0.71 to 2.01) | -1.89(-2.79 to -0.98) |
| **Djibouti** | 0(0 to 0) | 0.04(0.00 to 0.15) | 51(20 to 99) | 16.05(6.26 to 30.92) | 22.22(20.93 to 23.53) | 2(0 to 10) | 2.73(0.22 to 10.42) | 2803(1069 to 5447) | 871.08(333.22 to 1691.49) | 20.81(19.61 to 22.03) |
| **Dominica** | 0(0 to 0) | 0.45(0.18 to 0.82) | 0(0 to 0) | 0.27(0.11 to 0.48) | -1.20(-2.32 to -0.07) | 4(1 to 7) | 24.34(9.48 to 44.47) | 3(1 to 5) | 15.51(6.32 to 28.08) | -1.09(-2.18 to 0.01) |
| **Dominican Republic** | 7(2 to 20) | 0.40(0.09 to 1.17) | 12(3 to 30) | 0.41(0.10 to 1.05) | -0.04(-0.87 to 0.80) | 451(117 to 1276) | 24.80(6.31 to 70.28) | 703(195 to 1713) | 24.77(6.89 to 60.25) | -0.16(-0.96 to 0.65) |
| **Ecuador** | 2(1 to 3) | 0.09(0.05 to 0.15) | 20(11 to 30) | 0.42(0.23 to 0.64) | 4.78(3.74 to 5.82) | 112(54 to 187) | 5.13(2.49 to 8.47) | 1179(634 to 1781) | 25.14(13.54 to 37.93) | 5.10(4.09 to 6.12) |
| **Egypt** | 2(1 to 3) | 0.01(0.01 to 0.02) | 9(4 to 13) | 0.03(0.02 to 0.05) | 2.94(2.19 to 3.69) | 91(41 to 159) | 0.77(0.35 to 1.32) | 458(236 to 734) | 1.85(0.96 to 2.95) | 2.87(1.96 to 3.78) |
| **El Salvador** | 2(1 to 3) | 0.16(0.07 to 0.28) | 5(3 to 8) | 0.28(0.15 to 0.44) | 1.72(0.57 to 2.89) | 106(48 to 186) | 8.99(4.11 to 15.67) | 277(142 to 445) | 15.87(8.16 to 25.44) | 1.84(0.69 to 3.01) |
| **Equatorial Guinea** | 2(1 to 4) | 1.93(0.64 to 4.52) | 152(56 to 325) | 47.61(17.64 to 100.43) | 10.86(9.12 to 12.62) | 111(37 to 255) | 120.29(40.57 to 275.09) | 9064(3366 to 19340) | 2763.10(1039.32 to 5840.41) | 10.68(9.02 to 12.37) |
| **Eritrea** | 33(9 to 85) | 4.70(1.34 to 12.08) | 44(18 to 85) | 3.01(1.24 to 5.81) | -1.26(-2.44 to -0.05) | 2045(570 to 5221) | 284.12(80.64 to 724.11) | 2489(1007 to 4832) | 169.01(69.29 to 324.45) | -1.51(-2.63 to -0.39) |
| **Estonia** | 0(0 to 0) | 0.04(0.02 to 0.08) | 1(0 to 1) | 0.22(0.11 to 0.35) | 5.41(4.21 to 6.63) | 9(3 to 18) | 2.35(0.83 to 4.45) | 42(21 to 69) | 13.28(6.69 to 21.90) | 5.72(4.05 to 7.41) |
| **Eswatini** | 2(0 to 5) | 0.83(0.19 to 2.40) | 232(79 to 497) | 80.66(27.77 to 173.39) | 15.77(14.50 to 17.06) | 111(26 to 321) | 56.45(13.40 to 162.72) | 14187(5274 to 29274) | 4831.48(1816.78 to 9947.39) | 15.22(14.11 to 16.34) |
| **Ethiopia** | 753(304 to 1534) | 7.49(3.05 to 15.24) | 728(305 to 1437) | 3.42(1.44 to 6.71) | -2.34(-3.34 to -1.32) | 45951(18699 to 91837) | 442.66(181.82 to 883.74) | 41863(18064 to 80637) | 190.95(82.95 to 364.70) | -2.51(-3.51 to -1.51) |
| **Fiji** | 1(0 to 1) | 0.38(0.18 to 0.60) | 1(1 to 2) | 0.54(0.29 to 0.84) | 1.23(0.24 to 2.22) | 37(17 to 61) | 20.29(9.61 to 32.86) | 68(36 to 107) | 29.84(15.90 to 46.62) | 1.29(0.33 to 2.26) |
| **Finland** | 0(0 to 0) | 0.02(0.01 to 0.03) | 0(0 to 0) | 0.01(0.01 to 0.02) | -1.68(-2.77 to -0.59) | 15(7 to 24) | 1.06(0.51 to 1.76) | 10(5 to 17) | 0.86(0.44 to 1.39) | -0.88(-1.82 to 0.07) |
| **France** | 28(12 to 50) | 0.18(0.08 to 0.34) | 7(4 to 11) | 0.04(0.02 to 0.07) | -4.45(-6.09 to -2.79) | 1650(698 to 3021) | 11.05(4.67 to 20.27) | 565(294 to 893) | 3.66(1.89 to 5.83) | -3.47(-5.21 to -1.70) |
| **Gabon** | 12(5 to 26) | 6.24(2.40 to 13.55) | 76(32 to 144) | 17.95(7.58 to 33.61) | 3.55(1.89 to 5.23) | 749(295 to 1593) | 375.73(150.38 to 794.97) | 4405(1938 to 8186) | 1018.86(452.22 to 1881.93) | 3.34(1.78 to 4.92) |
| **Gambia** | 1(0 to 2) | 0.43(0.12 to 1.05) | 37(15 to 73) | 7.51(3.04 to 14.51) | 9.54(8.23 to 10.87) | 54(15 to 131) | 26.39(7.50 to 63.70) | 2148(867 to 4206) | 425.22(174.57 to 818.76) | 9.21(7.90 to 10.54) |
| **Georgia** | 0(0 to 0) | 0.00(0.00 to 0.01) | 0(0 to 0) | 0.01(0.00 to 0.02) | 2.50(1.79 to 3.21) | 3(1 to 7) | 0.24(0.10 to 0.46) | 5(3 to 9) | 0.56(0.27 to 0.96) | 2.74(2.08 to 3.41) |
| **Germany** | 20(10 to 30) | 0.09(0.05 to 0.15) | 6(3 to 9) | 0.03(0.02 to 0.05) | -3.56(-5.15 to -1.93) | 1159(608 to 1798) | 5.58(2.92 to 8.67) | 448(229 to 712) | 2.31(1.18 to 3.69) | -2.80(-4.05 to -1.54) |
| **Ghana** | 102(32 to 224) | 3.28(1.07 to 7.10) | 506(216 to 983) | 6.18(2.68 to 11.87) | 2.18(1.47 to 2.89) | 6172(1984 to 13561) | 192.43(63.01 to 415.51) | 28839(12460 to 56719) | 346.06(152.13 to 673.01) | 2.02(1.35 to 2.68) |
| **Greece** | 0(0 to 0) | 0.01(0.00 to 0.02) | 0(0 to 0) | 0.01(0.00 to 0.01) | -1.56(-2.33 to -0.78) | 13(6 to 24) | 0.52(0.23 to 0.96) | 11(6 to 19) | 0.47(0.23 to 0.80) | -0.52(-1.14 to 0.11) |
| **Greenland** | 0(0 to 0) | 0.25(0.10 to 0.43) | 0(0 to 0) | 0.15(0.07 to 0.26) | -1.81(-3.86 to 0.29) | 2(1 to 3) | 13.60(5.68 to 24.20) | 1(1 to 2) | 9.50(4.43 to 16.37) | -1.41(-3.22 to 0.44) |
| **Grenada** | 0(0 to 0) | 0.49(0.19 to 0.92) | 0(0 to 0) | 0.22(0.10 to 0.41) | -1.91(-2.97 to -0.84) | 5(2 to 9) | 26.67(10.31 to 50.07) | 3(1 to 6) | 12.39(5.43 to 22.56) | -1.86(-2.89 to -0.83) |
| **Guam** | 0(0 to 0) | 0.13(0.07 to 0.21) | 0(0 to 0) | 0.67(0.36 to 1.05) | 5.67(2.75 to 8.68) | 2(1 to 4) | 7.10(3.46 to 11.48) | 13(7 to 21) | 36.00(19.13 to 56.81) | 5.66(2.78 to 8.62) |
| **Guatemala** | 7(3 to 11) | 0.43(0.21 to 0.71) | 8(4 to 12) | 0.20(0.10 to 0.31) | -2.54(-3.38 to -1.68) | 367(176 to 620) | 23.36(11.27 to 39.07) | 445(229 to 714) | 10.97(5.68 to 17.48) | -2.46(-3.29 to -1.61) |
| **Guinea** | 16(5 to 39) | 1.26(0.37 to 3.00) | 119(42 to 237) | 4.36(1.58 to 8.56) | 3.98(3.09 to 4.89) | 1019(307 to 2407) | 77.86(23.42 to 182.57) | 7012(2580 to 13866) | 249.90(93.32 to 485.22) | 3.74(2.90 to 4.58) |
| **Guinea-Bissau** | 1(0 to 4) | 0.69(0.18 to 1.75) | 32(9 to 78) | 7.14(2.06 to 17.21) | 7.69(6.84 to 8.55) | 91(22 to 230) | 43.02(10.80 to 107.67) | 1945(571 to 4645) | 419.15(123.19 to 998.81) | 7.53(6.64 to 8.43) |
| **Guyana** | 2(1 to 5) | 1.43(0.59 to 2.69) | 3(1 to 6) | 1.68(0.74 to 3.01) | 0.70(-1.53 to 2.97) | 139(56 to 270) | 77.60(31.71 to 147.51) | 186(79 to 342) | 95.21(41.04 to 174.01) | 0.82(-1.39 to 3.08) |
| **Haiti** | 153(52 to 324) | 10.92(3.73 to 23.23) | 129(51 to 248) | 3.79(1.50 to 7.23) | -3.27(-4.40 to -2.12) | 8972(3108 to 18838) | 624.13(216.71 to 1312.47) | 7830(3098 to 14868) | 226.16(90.07 to 428.20) | -3.15(-4.49 to -1.78) |
| **Honduras** | 4(2 to 7) | 0.47(0.22 to 0.80) | 4(2 to 6) | 0.15(0.08 to 0.24) | -3.52(-3.62 to -3.42) | 245(111 to 424) | 26.08(12.01 to 44.54) | 243(130 to 383) | 8.82(4.73 to 13.86) | -3.39(-3.48 to -3.29) |
| **Hungary** | 1(0 to 2) | 0.03(0.01 to 0.07) | 1(0 to 1) | 0.02(0.01 to 0.04) | -0.92(-1.72 to -0.12) | 52(19 to 105) | 1.90(0.69 to 3.89) | 30(14 to 51) | 1.23(0.59 to 2.18) | -0.94(-1.73 to -0.16) |
| **Iceland** | 0(0 to 0) | 0.00(0.00 to 0.01) | 0(0 to 0) | 0.00(0.00 to 0.00) | -1.95(-3.19 to -0.70) | 0(0 to 0) | 0.20(0.07 to 0.43) | 0(0 to 0) | 0.15(0.06 to 0.32) | -0.75(-1.67 to 0.18) |
| **India** | 3(1 to 9) | 0.00(0.00 to 0.01) | 894(436 to 1532) | 0.24(0.12 to 0.42) | 17.30(15.40 to 19.23) | 284(89 to 670) | 0.15(0.05 to 0.35) | 50628(24909 to 85770) | 13.73(6.77 to 23.22) | 15.63(14.48 to 16.78) |
| **Indonesia** | 8(3 to 17) | 0.02(0.01 to 0.04) | 55(24 to 99) | 0.07(0.03 to 0.13) | 4.13(3.99 to 4.27) | 449(142 to 947) | 1.05(0.34 to 2.20) | 3153(1368 to 5759) | 4.02(1.73 to 7.36) | 4.40(4.25 to 4.55) |
| **Iran (Islamic Republic of)** | 0(0 to 0) | 0.00(0.00 to 0.00) | 5(3 to 9) | 0.02(0.01 to 0.03) | 7.27(4.89 to 9.71) | 14(6 to 25) | 0.13(0.05 to 0.23) | 325(159 to 554) | 1.24(0.59 to 2.14) | 7.56(5.45 to 9.71) |
| **Iraq** | 0(0 to 0) | 0.01(0.00 to 0.01) | 2(1 to 3) | 0.02(0.01 to 0.03) | 3.80(3.26 to 4.34) | 12(5 to 20) | 0.35(0.17 to 0.59) | 112(54 to 189) | 1.14(0.56 to 1.92) | 3.79(3.25 to 4.32) |
| **Ireland** | 0(0 to 0) | 0.02(0.01 to 0.03) | 0(0 to 0) | 0.01(0.00 to 0.01) | -2.24(-3.43 to -1.04) | 9(3 to 16) | 0.99(0.41 to 1.82) | 10(5 to 18) | 0.81(0.39 to 1.42) | -0.71(-1.55 to 0.14) |
| **Israel** | 1(0 to 1) | 0.07(0.04 to 0.10) | 1(0 to 1) | 0.03(0.02 to 0.05) | -2.54(-3.87 to -1.20) | 45(24 to 69) | 3.76(2.03 to 5.76) | 58(28 to 96) | 2.56(1.26 to 4.26) | -1.23(-2.20 to -0.25) |
| **Italy** | 31(14 to 53) | 0.22(0.10 to 0.37) | 9(5 to 13) | 0.06(0.03 to 0.09) | -4.21(-5.75 to -2.63) | 1956(852 to 3353) | 13.67(5.95 to 23.42) | 617(349 to 949) | 4.30(2.42 to 6.64) | -3.69(-4.93 to -2.44) |
| **Jamaica** | 3(1 to 6) | 0.62(0.25 to 1.20) | 8(4 to 12) | 1.03(0.53 to 1.60) | 1.91(1.06 to 2.76) | 175(70 to 341) | 34.07(13.70 to 65.64) | 450(233 to 710) | 57.48(29.72 to 90.67) | 1.96(1.15 to 2.77) |
| **Japan** | 0(0 to 1) | 0.00(0.00 to 0.00) | 1(0 to 1) | 0.00(0.00 to 0.00) | 2.59(1.91 to 3.27) | 24(10 to 44) | 0.07(0.03 to 0.13) | 58(26 to 101) | 0.21(0.09 to 0.37) | 3.51(3.01 to 4.02) |
| **Jordan** | 0(0 to 0) | 0.01(0.00 to 0.01) | 1(0 to 1) | 0.02(0.01 to 0.03) | 4.06(2.85 to 5.29) | 2(1 to 3) | 0.31(0.15 to 0.51) | 29(16 to 46) | 1.02(0.54 to 1.60) | 3.92(2.74 to 5.12) |
| **Kazakhstan** | 1(0 to 1) | 0.01(0.00 to 0.03) | 2(1 to 3) | 0.04(0.02 to 0.06) | 3.58(2.63 to 4.53) | 30(9 to 67) | 0.72(0.23 to 1.63) | 121(59 to 208) | 2.31(1.11 to 4.00) | 3.86(3.00 to 4.72) |
| **Kenya** | 951(353 to 1930) | 22.03(8.32 to 44.20) | 1449(661 to 2643) | 12.51(5.69 to 22.97) | -1.67(-2.83 to -0.49) | 58424(22066 to 117780) | 1291.16(498.30 to 2568.64) | 88510(41147 to 157100) | 742.98(344.91 to 1322.02) | -1.65(-2.59 to -0.69) |
| **Kiribati** | 0(0 to 0) | 0.51(0.30 to 0.73) | 0(0 to 0) | 0.51(0.30 to 0.74) | -0.03(-2.38 to 2.38) | 5(3 to 7) | 28.04(16.17 to 40.33) | 9(5 to 13) | 27.75(16.18 to 40.35) | -0.04(-2.38 to 2.36) |
| **Kuwait** | 0(0 to 0) | 0.01(0.01 to 0.02) | 0(0 to 0) | 0.01(0.00 to 0.01) | -1.12(-2.51 to 0.29) | 3(1 to 5) | 0.74(0.32 to 1.30) | 8(3 to 13) | 0.44(0.19 to 0.74) | -1.17(-2.50 to 0.17) |
| **Kyrgyzstan** | 1(0 to 1) | 0.06(0.02 to 0.11) | 3(2 to 5) | 0.18(0.10 to 0.27) | 3.86(3.30 to 4.42) | 31(12 to 57) | 3.20(1.26 to 5.86) | 180(96 to 275) | 10.33(5.51 to 15.80) | 3.99(3.49 to 4.50) |
| **Lao People's Democratic Republic** | 0(0 to 0) | 0.00(0.00 to 0.00) | 2(0 to 5) | 0.10(0.02 to 0.27) | 20.30(17.77 to 22.89) | 0(0 to 1) | 0.02(0.01 to 0.06) | 118(33 to 290) | 6.27(1.77 to 15.40) | 19.68(17.52 to 21.88) |
| **Latvia** | 1(0 to 1) | 0.08(0.03 to 0.13) | 6(3 to 9) | 1.35(0.75 to 2.02) | 9.57(8.79 to 10.34) | 30(13 to 51) | 4.52(1.95 to 7.71) | 337(188 to 505) | 76.61(42.47 to 115.36) | 9.53(8.81 to 10.25) |
| **Lebanon** | 0(0 to 0) | 0.02(0.01 to 0.05) | 2(1 to 4) | 0.13(0.05 to 0.24) | 5.65(5.52 to 5.77) | 9(3 to 18) | 1.27(0.45 to 2.58) | 112(45 to 215) | 6.85(2.72 to 13.13) | 5.58(5.47 to 5.69) |
| **Lesotho** | 35(12 to 75) | 9.87(3.43 to 21.39) | 402(165 to 779) | 91.77(37.91 to 177.51) | 7.63(4.00 to 11.39) | 2257(836 to 4712) | 629.43(233.73 to 1313.44) | 23787(10093 to 44670) | 5301.58(2259.25 to 9905.37) | 7.30(3.89 to 10.82) |
| **Liberia** | 7(2 to 19) | 1.35(0.41 to 3.57) | 63(26 to 121) | 5.16(2.11 to 9.88) | 4.31(3.55 to 5.08) | 442(142 to 1122) | 82.14(26.42 to 207.95) | 3607(1544 to 6796) | 290.09(124.81 to 544.12) | 4.06(3.34 to 4.79) |
| **Libya** | 0(0 to 1) | 0.03(0.00 to 0.13) | 3(0 to 25) | 0.12(0.00 to 1.19) | 5.03(4.69 to 5.36) | 10(0 to 52) | 1.45(0.06 to 7.28) | 138(1 to 1368) | 6.56(0.03 to 64.83) | 5.01(4.68 to 5.33) |
| **Lithuania** | 1(0 to 1) | 0.09(0.04 to 0.15) | 4(2 to 5) | 0.57(0.31 to 0.88) | 6.29(5.63 to 6.95) | 46(20 to 81) | 4.90(2.15 to 8.61) | 195(107 to 297) | 31.70(17.26 to 48.63) | 6.16(4.01 to 8.36) |
| **Luxembourg** | 0(0 to 0) | 0.05(0.02 to 0.08) | 0(0 to 0) | 0.02(0.01 to 0.03) | -2.66(-3.35 to -1.97) | 3(1 to 5) | 2.77(1.29 to 4.84) | 3(2 to 5) | 1.78(0.89 to 2.99) | -1.63(-2.29 to -0.98) |
| **Madagascar** | 0(0 to 0) | 0.00(0.00 to 0.01) | 183(69 to 361) | 3.03(1.17 to 5.90) | 26.29(24.40 to 28.20) | 4(1 to 13) | 0.16(0.03 to 0.51) | 10180(3877 to 19982) | 164.82(63.88 to 318.50) | 25.30(23.28 to 27.35) |
| **Malawi** | 761(291 to 1541) | 38.66(14.81 to 78.11) | 875(358 to 1719) | 22.24(9.12 to 43.62) | -1.83(-2.73 to -0.92) | 47714(19012 to 93764) | 2326.26(930.86 to 4565.14) | 53444(23227 to 100948) | 1318.92(574.92 to 2478.06) | -1.95(-2.52 to -1.38) |
| **Malaysia** | 1(0 to 2) | 0.03(0.01 to 0.06) | 13(6 to 24) | 0.16(0.07 to 0.28) | 6.03(4.01 to 8.08) | 65(21 to 137) | 1.53(0.50 to 3.20) | 792(347 to 1419) | 9.34(4.11 to 16.68) | 6.11(4.25 to 8.00) |
| **Maldives** | 0(0 to 0) | 0.01(0.00 to 0.02) | 0(0 to 0) | 0.04(0.02 to 0.07) | 4.93(4.48 to 5.39) | 0(0 to 0) | 0.53(0.20 to 1.05) | 3(1 to 5) | 2.20(1.07 to 3.80) | 4.76(4.34 to 5.19) |
| **Mali** | 22(7 to 55) | 1.24(0.37 to 3.08) | 186(76 to 381) | 4.35(1.81 to 8.74) | 4.04(2.93 to 5.16) | 1408(436 to 3392) | 76.90(23.94 to 184.63) | 10936(4594 to 22079) | 246.99(105.52 to 488.83) | 3.74(2.69 to 4.80) |
| **Malta** | 0(0 to 0) | 0.02(0.01 to 0.05) | 0(0 to 0) | 0.01(0.01 to 0.02) | -2.01(-3.01 to -1.00) | 1(1 to 3) | 1.41(0.59 to 2.59) | 1(1 to 2) | 1.24(0.59 to 2.19) | -0.41(-1.17 to 0.37) |
| **Marshall Islands** | 0(0 to 0) | 0.03(0.00 to 0.07) | 0(0 to 0) | 0.99(0.35 to 2.06) | 12.03(11.06 to 13.01) | 0(0 to 0) | 1.65(0.29 to 4.07) | 8(3 to 17) | 56.04(20.57 to 115.22) | 11.89(10.96 to 12.84) |
| **Mauritania** | 0(0 to 0) | 0.01(0.00 to 0.02) | 0(0 to 0) | 0.00(0.00 to 0.01) | -2.07(-2.73 to -1.39) | 2(1 to 5) | 0.52(0.14 to 1.15) | 4(1 to 10) | 0.48(0.11 to 1.13) | -0.37(-0.63 to -0.11) |
| **Mauritius** | 0(0 to 0) | 0.02(0.01 to 0.04) | 1(0 to 1) | 0.19(0.08 to 0.34) | 8.59(4.51 to 12.82) | 3(1 to 6) | 1.10(0.42 to 2.16) | 37(16 to 66) | 10.83(4.71 to 19.53) | 8.66(3.06 to 14.58) |
| **Mexico** | 49(26 to 75) | 0.27(0.14 to 0.41) | 89(49 to 137) | 0.25(0.14 to 0.38) | -0.23(-0.73 to 0.27) | 2717(1453 to 4217) | 14.45(7.78 to 22.26) | 5006(2691 to 7793) | 14.00(7.50 to 21.82) | -0.12(-0.58 to 0.34) |
| **Micronesia (Federated States of)** | 0(0 to 0) | 0.03(0.00 to 0.08) | 0(0 to 1) | 1.06(0.38 to 2.18) | 12.07(11.05 to 13.10) | 0(0 to 1) | 1.79(0.32 to 4.45) | 15(5 to 30) | 60.14(22.02 to 122.62) | 11.84(10.87 to 12.83) |
| **Monaco** | 0(0 to 0) | 0.02(0.01 to 0.05) | 0(0 to 0) | 0.01(0.00 to 0.02) | -2.68(-3.29 to -2.06) | 0(0 to 0) | 1.23(0.34 to 3.01) | 0(0 to 0) | 0.65(0.24 to 1.41) | -2.02(-2.62 to -1.43) |
| **Mongolia** | 0(0 to 0) | 0.00(0.00 to 0.00) | 0(0 to 0) | 0.02(0.01 to 0.03) | 19.03(17.21 to 20.88) | 0(0 to 0) | 0.00(0.00 to 0.00) | 10(6 to 16) | 1.13(0.61 to 1.73) | 19.19(17.50 to 20.91) |
| **Montenegro** | 0(0 to 0) | 0.01(0.00 to 0.02) | 0(0 to 0) | 0.01(0.00 to 0.01) | -0.64(-1.35 to 0.07) | 1(0 to 2) | 0.55(0.23 to 1.03) | 1(0 to 1) | 0.48(0.20 to 0.91) | -0.39(-1.11 to 0.34) |
| **Morocco** | 3(1 to 7) | 0.05(0.01 to 0.12) | 7(2 to 18) | 0.07(0.02 to 0.18) | 0.94(0.28 to 1.61) | 163(47 to 398) | 2.88(0.85 to 6.92) | 422(135 to 1008) | 4.27(1.36 to 10.20) | 1.12(0.50 to 1.74) |
| **Mozambique** | 93(40 to 179) | 3.21(1.39 to 6.18) | 2509(1325 to 4061) | 39.15(21.02 to 62.38) | 8.18(7.15 to 9.22) | 6243(2656 to 12039) | 211.16(90.70 to 403.53) | 154119(80835 to 250515) | 2349.12(1259.05 to 3748.44) | 7.86(6.82 to 8.90) |
| **Myanmar** | 1(0 to 3) | 0.01(0.00 to 0.03) | 112(35 to 243) | 0.74(0.23 to 1.61) | 14.94(13.11 to 16.81) | 130(41 to 291) | 1.28(0.41 to 2.84) | 6683(2247 to 13871) | 44.55(14.98 to 92.31) | 12.35(10.38 to 14.36) |
| **Namibia** | 14(5 to 31) | 4.89(1.69 to 10.57) | 113(44 to 235) | 19.00(7.37 to 39.27) | 4.32(2.84 to 5.83) | 950(349 to 1970) | 311.33(115.97 to 644.54) | 6697(2705 to 13506) | 1109.16(452.84 to 2223.83) | 4.03(2.64 to 5.44) |
| **Nauru** | 0(0 to 0) | 0.01(0.00 to 0.03) | 0(0 to 0) | 0.36(0.10 to 0.79) | 11.52(10.64 to 12.40) | 0(0 to 0) | 0.73(0.14 to 1.78) | 1(0 to 1) | 20.88(6.41 to 45.19) | 11.35(10.50 to 12.20) |
| **Netherlands** | 2(1 to 3) | 0.05(0.03 to 0.08) | 1(0 to 1) | 0.02(0.01 to 0.03) | -2.92(-4.45 to -1.36) | 127(69 to 198) | 3.08(1.66 to 4.81) | 69(34 to 119) | 1.76(0.88 to 3.03) | -1.76(-2.81 to -0.70) |
| **New Zealand** | 0(0 to 0) | 0.03(0.01 to 0.04) | 0(0 to 0) | 0.01(0.00 to 0.01) | -3.34(-4.68 to -1.99) | 14(7 to 22) | 1.52(0.76 to 2.40) | 10(5 to 17) | 0.78(0.35 to 1.36) | -2.07(-3.14 to -0.97) |
| **Nicaragua** | 1(0 to 1) | 0.10(0.05 to 0.17) | 3(2 to 5) | 0.17(0.09 to 0.27) | 1.53(0.96 to 2.11) | 42(20 to 69) | 5.55(2.68 to 9.22) | 180(96 to 279) | 10.02(5.35 to 15.55) | 1.80(1.27 to 2.33) |
| **Niger** | 12(3 to 31) | 0.78(0.22 to 2.03) | 25(8 to 57) | 0.64(0.20 to 1.46) | -0.63(-1.76 to 0.51) | 752(212 to 1940) | 47.92(13.64 to 121.97) | 1477(474 to 3338) | 36.92(12.12 to 82.59) | -0.87(-1.93 to 0.21) |
| **Nigeria** | 345(135 to 693) | 1.94(0.76 to 3.86) | 2909(1422 to 4914) | 6.39(3.15 to 10.69) | 3.95(2.90 to 5.01) | 21738(8711 to 42779) | 117.13(47.39 to 229.12) | 164089(81316 to 274294) | 352.48(176.15 to 582.60) | 3.64(2.63 to 4.66) |
| **Niue** | 0(0 to 0) | 0.01(0.00 to 0.03) | 0(0 to 0) | 0.29(0.08 to 0.68) | 10.46(9.16 to 11.77) | 0(0 to 0) | 0.78(0.14 to 1.87) | 0(0 to 0) | 17.03(5.02 to 38.63) | 10.40(9.17 to 11.64) |
| **North Macedonia** | 0(0 to 0) | 0.00(0.00 to 0.01) | 0(0 to 0) | 0.00(0.00 to 0.01) | 0.67(-0.31 to 1.65) | 1(0 to 2) | 0.16(0.06 to 0.30) | 1(0 to 2) | 0.16(0.07 to 0.29) | 0.54(-0.74 to 1.84) |
| **Northern Mariana Islands** | 0(0 to 0) | 0.17(0.08 to 0.28) | 0(0 to 0) | 0.43(0.22 to 0.66) | 3.84(0.66 to 7.11) | 1(1 to 2) | 9.35(4.42 to 15.15) | 3(1 to 4) | 23.41(11.77 to 36.78) | 3.86(0.80 to 7.02) |
| **Norway** | 0(0 to 0) | 0.01(0.01 to 0.02) | 0(0 to 0) | 0.02(0.01 to 0.03) | 0.66(-5.56 to 7.29) | 9(5 to 13) | 0.81(0.43 to 1.24) | 23(11 to 41) | 1.81(0.85 to 3.29) | 2.53(-0.15 to 5.28) |
| **Oman** | 0(0 to 0) | 0.03(0.01 to 0.05) | 1(1 to 2) | 0.13(0.06 to 0.22) | 4.55(4.23 to 4.88) | 5(2 to 9) | 1.72(0.74 to 2.94) | 81(37 to 140) | 7.30(3.30 to 12.76) | 4.76(4.46 to 5.07) |
| **Pakistan** | 0(0 to 0) | 0.00(0.00 to 0.00) | 73(0 to 440) | 0.13(0.00 to 0.79) | 38.91(36.15 to 41.74) | 0(0 to 0) | 0.00(0.00 to 0.00) | 4151(25 to 24880) | 7.28(0.04 to 44.12) | 37.80(35.65 to 39.99) |
| **Palau** | 0(0 to 0) | 0.01(0.00 to 0.03) | 0(0 to 0) | 0.29(0.08 to 0.67) | 10.66(9.74 to 11.59) | 0(0 to 0) | 0.74(0.14 to 1.76) | 1(0 to 2) | 17.12(5.08 to 38.21) | 10.60(9.72 to 11.49) |
| **Palestine** | 0(0 to 0) | 0.00(0.00 to 0.01) | 0(0 to 0) | 0.03(0.02 to 0.04) | 7.94(6.56 to 9.33) | 1(0 to 1) | 0.23(0.13 to 0.33) | 19(10 to 30) | 1.63(0.87 to 2.52) | 7.80(5.59 to 10.07) |
| **Panama** | 2(1 to 5) | 0.45(0.17 to 0.84) | 6(3 to 11) | 0.60(0.28 to 1.05) | 0.92(0.56 to 1.28) | 139(53 to 267) | 24.97(9.59 to 47.26) | 382(176 to 668) | 35.77(16.50 to 62.59) | 1.10(0.72 to 1.49) |
| **Papua New Guinea** | 0(0 to 0) | 0.01(0.00 to 0.02) | 36(13 to 77) | 1.50(0.53 to 3.17) | 19.54(17.78 to 21.34) | 4(1 to 10) | 0.42(0.11 to 1.12) | 2261(925 to 4469) | 92.02(37.76 to 181.92) | 19.01(15.92 to 22.18) |
| **Paraguay** | 0(0 to 1) | 0.06(0.03 to 0.11) | 3(2 to 5) | 0.19(0.10 to 0.30) | 3.96(2.90 to 5.03) | 28(12 to 51) | 3.25(1.44 to 5.95) | 198(103 to 318) | 10.74(5.59 to 17.17) | 4.00(3.01 to 5.01) |
| **Peru** | 9(5 to 13) | 0.19(0.11 to 0.29) | 25(14 to 37) | 0.25(0.14 to 0.38) | 0.97(-0.51 to 2.47) | 502(273 to 762) | 10.77(5.92 to 16.14) | 1443(792 to 2160) | 14.69(8.05 to 21.99) | 1.10(-0.28 to 2.50) |
| **Philippines** | 0(0 to 0) | 0.00(0.00 to 0.00) | 41(21 to 65) | 0.15(0.08 to 0.23) | 20.11(17.29 to 23.00) | 3(1 to 6) | 0.02(0.01 to 0.04) | 2403(1220 to 3903) | 8.56(4.37 to 13.82) | 21.55(15.18 to 28.27) |
| **Poland** | 0(0 to 0) | 0.00(0.00 to 0.00) | 1(1 to 2) | 0.01(0.01 to 0.02) | 7.76(5.50 to 10.07) | 8(3 to 14) | 0.08(0.03 to 0.14) | 67(34 to 109) | 0.65(0.32 to 1.07) | 7.51(5.38 to 9.68) |
| **Portugal** | 4(2 to 8) | 0.17(0.07 to 0.32) | 3(1 to 4) | 0.10(0.05 to 0.15) | -1.90(-3.08 to -0.70) | 247(99 to 463) | 9.82(3.94 to 18.45) | 157(82 to 250) | 5.86(3.04 to 9.36) | -1.68(-2.79 to -0.56) |
| **Puerto Rico** | 9(4 to 15) | 0.94(0.46 to 1.56) | 2(1 to 3) | 0.22(0.11 to 0.36) | -4.41(-6.19 to -2.61) | 496(243 to 828) | 52.24(25.54 to 87.19) | 96(49 to 159) | 12.07(6.11 to 20.12) | -4.46(-6.18 to -2.70) |
| **Qatar** | 0(0 to 0) | 0.05(0.02 to 0.08) | 0(0 to 0) | 0.03(0.01 to 0.05) | -1.20(-3.54 to 1.21) | 2(1 to 4) | 2.64(1.13 to 4.60) | 10(4 to 18) | 1.53(0.68 to 2.80) | -1.23(-2.89 to 0.45) |
| **Republic of Korea** | 0(0 to 0) | 0.00(0.00 to 0.00) | 0(0 to 0) | 0.00(0.00 to 0.00) | 1.09(0.46 to 1.73) | 11(0 to 25) | 0.09(0.00 to 0.20) | 16(7 to 29) | 0.12(0.05 to 0.22) | 0.95(0.34 to 1.56) |
| **Republic of Moldova** | 0(0 to 0) | 0.01(0.01 to 0.02) | 3(2 to 5) | 0.31(0.16 to 0.48) | 10.46(9.50 to 11.44) | 9(4 to 15) | 0.76(0.37 to 1.28) | 187(98 to 293) | 17.77(9.21 to 28.01) | 10.65(9.82 to 11.48) |
| **Romania** | 2(1 to 3) | 0.04(0.02 to 0.06) | 2(1 to 4) | 0.06(0.03 to 0.09) | 1.71(0.36 to 3.08) | 112(54 to 196) | 1.99(0.95 to 3.47) | 148(73 to 247) | 3.92(1.92 to 6.58) | 2.39(1.15 to 3.65) |
| **Russian Federation** | 45(23 to 72) | 0.11(0.06 to 0.18) | 317(159 to 511) | 0.78(0.38 to 1.28) | 6.42(5.51 to 7.33) | 2554(1276 to 4124) | 6.43(3.19 to 10.44) | 18391(8946 to 29830) | 46.19(22.03 to 76.44) | 6.66(5.82 to 7.51) |
| **Rwanda** | 130(42 to 364) | 9.43(3.07 to 26.44) | 189(74 to 374) | 6.27(2.44 to 12.38) | -1.21(-1.87 to -0.55) | 7974(2597 to 21600) | 556.02(183.86 to 1509.35) | 11030(4543 to 21361) | 358.65(148.61 to 692.16) | -1.31(-1.89 to -0.72) |
| **Saint Kitts and Nevis** | 0(0 to 0) | 0.14(0.03 to 0.40) | 0(0 to 0) | 1.04(0.37 to 2.10) | 6.55(5.68 to 7.42) | 1(0 to 2) | 8.20(1.83 to 22.81) | 10(3 to 19) | 56.20(20.15 to 114.70) | 6.32(5.38 to 7.27) |
| **Saint Lucia** | 0(0 to 0) | 0.48(0.19 to 0.87) | 0(0 to 0) | 0.19(0.09 to 0.36) | -2.55(-3.28 to -1.82) | 7(3 to 14) | 25.53(10.16 to 47.14) | 5(2 to 10) | 10.80(4.73 to 20.15) | -2.30(-3.29 to -1.29) |
| **Saint Vincent and the Grenadines** | 0(0 to 1) | 1.43(0.54 to 2.68) | 0(0 to 0) | 0.84(0.36 to 1.53) | -1.34(-2.55 to -0.11) | 17(6 to 33) | 77.26(29.05 to 145.53) | 13(6 to 24) | 46.92(19.45 to 86.32) | -1.26(-2.43 to -0.07) |
| **Samoa** | 0(0 to 0) | 0.02(0.00 to 0.06) | 0(0 to 1) | 0.71(0.25 to 1.51) | 11.65(10.78 to 12.51) | 0(0 to 1) | 1.32(0.24 to 3.17) | 18(6 to 37) | 39.66(14.40 to 83.56) | 11.48(10.64 to 12.32) |
| **San Marino** | 0(0 to 0) | 0.02(0.01 to 0.05) | 0(0 to 0) | 0.01(0.00 to 0.02) | -3.40(-4.16 to -2.64) | 0(0 to 0) | 1.21(0.34 to 3.00) | 0(0 to 0) | 0.55(0.19 to 1.24) | -2.57(-3.19 to -1.93) |
| **Sao Tome and Principe** | 0(0 to 0) | 0.01(0.00 to 0.01) | 0(0 to 0) | 0.00(0.00 to 0.01) | -0.99(-1.64 to -0.33) | 0(0 to 0) | 0.37(0.13 to 0.78) | 0(0 to 0) | 0.39(0.17 to 0.68) | 0.15(-0.33 to 0.63) |
| **Saudi Arabia** | 3(1 to 5) | 0.10(0.05 to 0.18) | 28(14 to 48) | 0.24(0.12 to 0.42) | 2.95(2.25 to 3.65) | 154(68 to 272) | 5.62(2.53 to 9.81) | 1517(717 to 2632) | 13.29(6.26 to 23.17) | 2.92(2.24 to 3.60) |
| **Senegal** | 10(3 to 23) | 0.66(0.19 to 1.57) | 34(12 to 70) | 1.05(0.38 to 2.14) | 1.39(0.48 to 2.31) | 597(175 to 1420) | 39.33(11.71 to 92.14) | 1981(748 to 3901) | 59.55(22.63 to 117.34) | 1.30(0.50 to 2.10) |
| **Serbia** | 0(0 to 0) | 0.00(0.00 to 0.01) | 0(0 to 0) | 0.01(0.00 to 0.01) | 2.38(1.00 to 3.77) | 6(0 to 15) | 0.24(0.00 to 0.63) | 10(4 to 19) | 0.45(0.19 to 0.87) | 2.24(1.06 to 3.43) |
| **Seychelles** | 0(0 to 0) | 0.06(0.02 to 0.13) | 0(0 to 0) | 0.36(0.16 to 0.63) | 6.08(3.97 to 8.23) | 1(0 to 1) | 3.49(1.08 to 7.36) | 5(2 to 9) | 19.67(8.73 to 34.45) | 5.88(3.84 to 7.97) |
| **Sierra Leone** | 9(3 to 22) | 0.93(0.32 to 2.35) | 97(38 to 186) | 5.15(2.04 to 9.88) | 5.63(4.57 to 6.71) | 542(192 to 1302) | 56.70(20.04 to 136.20) | 5706(2380 to 10667) | 295.64(123.45 to 549.05) | 5.42(4.42 to 6.43) |
| **Singapore** | 0(0 to 0) | 0.00(0.00 to 0.01) | 0(0 to 0) | 0.00(0.00 to 0.01) | -0.58(-1.22 to 0.06) | 2(1 to 4) | 0.23(0.09 to 0.42) | 6(3 to 13) | 0.35(0.14 to 0.72) | 1.26(0.71 to 1.81) |
| **Slovakia** | 0(0 to 0) | 0.00(0.00 to 0.01) | 0(0 to 0) | 0.00(0.00 to 0.00) | -0.51(-1.78 to 0.78) | 2(1 to 4) | 0.17(0.07 to 0.30) | 2(1 to 3) | 0.15(0.08 to 0.25) | -0.31(-1.54 to 0.94) |
| **Slovenia** | 0(0 to 0) | 0.00(0.00 to 0.01) | 0(0 to 0) | 0.00(0.00 to 0.00) | -1.08(-2.69 to 0.57) | 1(0 to 2) | 0.19(0.00 to 0.40) | 1(0 to 1) | 0.14(0.07 to 0.24) | -0.50(-1.30 to 0.30) |
| **Solomon Islands** | 0(0 to 0) | 0.03(0.00 to 0.08) | 2(1 to 3) | 1.08(0.39 to 2.09) | 11.85(10.74 to 12.97) | 1(0 to 3) | 1.91(0.35 to 4.41) | 97(36 to 188) | 60.65(22.54 to 116.76) | 11.64(10.60 to 12.70) |
| **Somalia** | 1(0 to 4) | 0.07(0.01 to 0.28) | 135(47 to 288) | 3.46(1.22 to 7.37) | 13.15(12.16 to 14.15) | 81(10 to 276) | 5.06(0.63 to 17.16) | 7380(2622 to 15626) | 185.59(67.11 to 389.72) | 12.13(11.31 to 12.96) |
| **South Africa** | 38(13 to 84) | 0.43(0.14 to 0.94) | 1618(739 to 2868) | 10.12(4.62 to 17.99) | 10.52(9.22 to 11.85) | 2534(827 to 5594) | 27.57(9.10 to 60.52) | 106400(47791 to 189445) | 663.95(297.97 to 1185.38) | 10.59(9.34 to 11.86) |
| **South Sudan** | 18(5 to 54) | 1.62(0.42 to 4.77) | 178(55 to 450) | 9.00(2.81 to 22.65) | 5.54(4.53 to 6.57) | 1110(300 to 3224) | 96.15(26.21 to 273.51) | 10231(3358 to 24802) | 511.49(168.74 to 1235.55) | 5.41(4.44 to 6.38) |
| **Spain** | 16(7 to 29) | 0.16(0.07 to 0.31) | 4(2 to 6) | 0.03(0.02 to 0.05) | -5.18(-6.21 to -4.13) | 994(411 to 1876) | 10.37(4.30 to 19.56) | 246(128 to 395) | 2.01(1.04 to 3.26) | -5.19(-6.07 to -4.30) |
| **Sri Lanka** | 1(0 to 1) | 0.01(0.01 to 0.02) | 2(1 to 3) | 0.03(0.02 to 0.05) | 3.66(1.70 to 5.66) | 29(13 to 49) | 0.66(0.31 to 1.13) | 100(53 to 159) | 1.68(0.88 to 2.69) | 3.43(1.59 to 5.31) |
| **Sudan** | 13(3 to 42) | 0.32(0.08 to 1.01) | 356(127 to 901) | 3.61(1.30 to 9.02) | 8.15(7.60 to 8.70) | 757(193 to 2416) | 18.03(4.65 to 57.23) | 19611(7094 to 49622) | 194.85(71.42 to 486.38) | 7.93(7.36 to 8.51) |
| **Suriname** | 1(0 to 2) | 1.30(0.52 to 2.41) | 2(1 to 3) | 1.15(0.52 to 1.99) | 0.31(-0.67 to 1.31) | 62(24 to 117) | 70.66(27.78 to 132.39) | 100(45 to 175) | 67.98(30.23 to 119.30) | 0.56(-0.41 to 1.53) |
| **Sweden** | 0(0 to 0) | 0.01(0.00 to 0.02) | 0(0 to 1) | 0.01(0.01 to 0.02) | 1.10(-2.81 to 5.15) | 14(6 to 25) | 0.65(0.28 to 1.17) | 29(14 to 46) | 1.20(0.59 to 1.93) | 2.11(-1.06 to 5.39) |
| **Switzerland** | 1(0 to 2) | 0.04(0.02 to 0.08) | 0(0 to 1) | 0.01(0.01 to 0.03) | -3.29(-4.01 to -2.57) | 49(19 to 95) | 2.60(1.00 to 5.06) | 25(11 to 49) | 1.14(0.47 to 2.23) | -2.46(-3.21 to -1.70) |
| **Syrian Arab Republic** | 0(0 to 0) | 0.01(0.00 to 0.02) | 1(0 to 2) | 0.03(0.01 to 0.05) | 2.89(2.21 to 3.57) | 12(5 to 22) | 0.56(0.25 to 0.98) | 52(25 to 88) | 1.43(0.69 to 2.43) | 2.85(2.18 to 3.52) |
| **Taiwan (Province of China)** | 0(0 to 0) | 0.00(0.00 to 0.00) | 1(0 to 1) | 0.01(0.01 to 0.02) | 4.11(2.76 to 5.47) | 6(2 to 12) | 0.11(0.04 to 0.21) | 42(20 to 73) | 0.64(0.30 to 1.13) | 3.91(2.63 to 5.21) |
| **Tajikistan** | 1(1 to 2) | 0.12(0.06 to 0.21) | 4(2 to 6) | 0.15(0.08 to 0.23) | 0.64(-0.37 to 1.67) | 69(32 to 120) | 6.77(3.18 to 11.69) | 214(118 to 327) | 8.61(4.74 to 13.18) | 0.89(-0.31 to 2.10) |
| **Thailand** | 274(142 to 432) | 1.72(0.89 to 2.70) | 179(95 to 284) | 0.97(0.51 to 1.55) | -1.48(-2.76 to -0.18) | 15910(8221 to 25179) | 98.40(51.04 to 155.23) | 10731(5588 to 17116) | 59.49(30.53 to 96.33) | -1.25(-2.48 to -0.01) |
| **Timor-Leste** | 0(0 to 0) | 0.04(0.01 to 0.12) | 5(2 to 10) | 2.00(0.90 to 3.53) | 13.08(12.41 to 13.75) | 6(2 to 14) | 2.90(0.83 to 7.38) | 297(130 to 538) | 107.25(48.07 to 190.08) | 12.19(11.55 to 12.83) |
| **Togo** | 19(5 to 48) | 2.57(0.74 to 6.35) | 69(24 to 142) | 3.48(1.23 to 7.20) | 0.99(0.22 to 1.77) | 1186(347 to 2917) | 153.41(45.84 to 372.24) | 4030(1517 to 7985) | 201.42(76.12 to 398.44) | 0.86(0.12 to 1.62) |
| **Tokelau** | 0(0 to 0) | 0.01(0.00 to 0.03) | 0(0 to 0) | 0.29(0.07 to 0.67) | 10.09(8.13 to 12.09) | 0(0 to 0) | 0.81(0.16 to 1.98) | 0(0 to 0) | 16.94(4.70 to 38.30) | 10.03(8.23 to 11.86) |
| **Tonga** | 0(0 to 0) | 0.07(0.03 to 0.12) | 0(0 to 0) | 0.12(0.07 to 0.18) | 1.76(1.52 to 2.01) | 1(0 to 1) | 3.99(1.89 to 6.55) | 2(1 to 3) | 9.24(4.90 to 14.35) | 2.76(2.53 to 2.99) |
| **Trinidad and Tobago** | 3(1 to 5) | 0.95(0.38 to 1.75) | 3(1 to 5) | 0.80(0.34 to 1.44) | -0.14(-1.03 to 0.76) | 150(58 to 278) | 51.14(20.13 to 94.64) | 176(76 to 318) | 47.39(20.12 to 86.80) | 0.13(-0.72 to 0.98) |
| **Tunisia** | 0(0 to 0) | 0.00(0.00 to 0.00) | 1(0 to 2) | 0.03(0.01 to 0.07) | 12.11(9.13 to 15.18) | 1(0 to 4) | 0.05(0.00 to 0.19) | 54(14 to 136) | 1.61(0.40 to 4.05) | 11.95(9.27 to 14.70) |
| **Turkey** | 1(0 to 1) | 0.00(0.00 to 0.01) | 7(4 to 11) | 0.03(0.02 to 0.05) | 6.43(5.86 to 7.00) | 29(0 to 54) | 0.22(0.00 to 0.41) | 396(218 to 601) | 1.70(0.93 to 2.60) | 6.76(5.85 to 7.67) |
| **Turkmenistan** | 1(0 to 1) | 0.07(0.02 to 0.14) | 1(0 to 2) | 0.09(0.04 to 0.17) | 1.02(-0.18 to 2.24) | 30(10 to 64) | 3.74(1.21 to 7.83) | 63(26 to 120) | 5.03(2.08 to 9.62) | 0.96(-0.20 to 2.14) |
| **Tuvalu** | 0(0 to 0) | 0.01(0.00 to 0.03) | 0(0 to 0) | 0.30(0.08 to 0.71) | 10.29(8.38 to 12.25) | 0(0 to 0) | 0.81(0.16 to 1.98) | 0(0 to 1) | 17.65(4.96 to 40.78) | 10.26(8.53 to 12.01) |
| **Uganda** | 3962(1577 to 7736) | 125.51(50.40 to 241.99) | 1689(653 to 3213) | 20.02(7.78 to 37.94) | -5.79(-6.36 to -5.21) | 233508(93007 to 454427) | 7088.63(2854.66 to 13604.23) | 104494(43725 to 192185) | 1202.73(508.56 to 2195.63) | -5.59(-6.13 to -5.04) |
| **Ukraine** | 13(6 to 22) | 0.09(0.04 to 0.16) | 74(36 to 128) | 0.59(0.28 to 1.02) | 6.36(5.52 to 7.21) | 685(303 to 1178) | 5.05(2.23 to 8.72) | 4078(1933 to 7118) | 32.83(15.21 to 58.35) | 6.41(5.59 to 7.23) |
| **United Arab Emirates** | 0(0 to 0) | 0.01(0.00 to 0.02) | 0(0 to 1) | 0.02(0.01 to 0.05) | 2.36(1.41 to 3.31) | 2(1 to 4) | 0.52(0.16 to 1.18) | 25(8 to 56) | 1.19(0.35 to 2.72) | 2.60(1.69 to 3.51) |
| **United Kingdom** | 2(1 to 4) | 0.02(0.01 to 0.03) | 5(3 to 8) | 0.03(0.02 to 0.05) | 1.96(-0.30 to 4.27) | 152(71 to 254) | 1.04(0.49 to 1.74) | 473(248 to 752) | 2.80(1.46 to 4.46) | 3.34(1.86 to 4.85) |
| **United Republic of Tanzania** | 1436(535 to 2870) | 28.68(10.78 to 56.92) | 1873(763 to 3566) | 14.71(6.02 to 27.92) | -2.19(-3.32 to -1.05) | 86832(33398 to 171224) | 1664.85(647.56 to 3254.16) | 116572(51160 to 213451) | 894.83(394.40 to 1630.32) | -2.02(-3.04 to -0.99) |
| **United States of America** | 307(171 to 459) | 0.43(0.24 to 0.64) | 80(43 to 120) | 0.10(0.05 to 0.15) | -4.73(-5.31 to -4.15) | 18069(10000 to 27091) | 25.04(13.85 to 37.56) | 5935(3118 to 9472) | 7.27(3.81 to 11.64) | -4.03(-4.55 to -3.49) |
| **United States Virgin Islands** | 0(0 to 0) | 0.59(0.26 to 1.02) | 0(0 to 0) | 0.32(0.17 to 0.52) | -2.28(-3.09 to -1.47) | 9(4 to 16) | 31.22(13.71 to 53.94) | 3(2 to 5) | 17.86(9.03 to 28.71) | -2.17(-2.96 to -1.37) |
| **Uruguay** | 1(0 to 1) | 0.08(0.03 to 0.15) | 2(1 to 3) | 0.23(0.11 to 0.40) | 3.68(3.30 to 4.07) | 34(14 to 64) | 4.50(1.87 to 8.59) | 122(58 to 213) | 14.14(6.63 to 24.86) | 3.98(3.63 to 4.33) |
| **Uzbekistan** | 1(0 to 2) | 0.03(0.01 to 0.06) | 5(2 to 9) | 0.05(0.02 to 0.09) | 2.03(1.12 to 2.95) | 64(18 to 139) | 1.43(0.41 to 3.11) | 258(101 to 503) | 2.75(1.08 to 5.39) | 2.10(1.21 to 3.00) |
| **Vanuatu** | 0(0 to 0) | 0.03(0.00 to 0.06) | 1(0 to 1) | 0.91(0.35 to 1.84) | 12.06(11.04 to 13.09) | 0(0 to 1) | 1.53(0.28 to 3.61) | 37(15 to 75) | 51.29(20.54 to 102.04) | 11.85(10.89 to 12.83) |
| **Venezuela (Bolivarian Republic of)** | 9(3 to 17) | 0.20(0.07 to 0.39) | 29(14 to 50) | 0.39(0.18 to 0.67) | 2.24(1.77 to 2.71) | 496(174 to 975) | 11.19(3.99 to 21.69) | 1637(754 to 2817) | 22.23(10.15 to 38.49) | 2.35(1.89 to 2.81) |
| **Viet Nam** | 3(1 to 6) | 0.02(0.00 to 0.04) | 57(23 to 119) | 0.21(0.08 to 0.43) | 9.02(7.88 to 10.19) | 203(69 to 425) | 1.12(0.38 to 2.35) | 3570(1483 to 7144) | 13.13(5.45 to 26.22) | 8.46(7.42 to 9.50) |
| **Yemen** | 0(0 to 1) | 0.01(0.00 to 0.04) | 10(3 to 24) | 0.13(0.03 to 0.31) | 7.61(7.35 to 7.87) | 19(3 to 53) | 0.79(0.13 to 2.20) | 582(156 to 1358) | 7.57(2.06 to 17.51) | 7.60(7.37 to 7.82) |
| **Zambia** | 996(396 to 2016) | 62.50(24.56 to 128.11) | 1278(490 to 2552) | 32.17(12.34 to 64.59) | -2.19(-2.98 to -1.39) | 63377(26353 to 123000) | 3789.86(1563.26 to 7451.84) | 77140(32261 to 146874) | 1890.21(795.47 to 3605.37) | -2.30(-3.04 to -1.55) |
| **Zimbabwe** | 1172(439 to 2449) | 55.77(20.87 to 117.02) | 1028(410 to 2065) | 29.35(11.68 to 59.18) | -2.03(-2.73 to -1.33) | 74813(29324 to 151233) | 3401.19(1338.79 to 6900.94) | 60934(26123 to 116874) | 1706.23(731.19 to 3277.19) | -2.19(-2.80 to -1.58) |

**Notes:** Rates are reported per 100,000 person-years. Data in parentheses are 95% uncertainty intervals for cases and age-standardized rates of mortality and DALYs, and 95% confidence intervals for AAPCs. **Abbreviations:** DALYs, disability-adjusted life-years; ASMR, age-standardized mortality rate; ASDR, age-standardized DALYs rate; AAPC, average annual percent change; SDI, socio-demographic index; UI, uncertainty interval; CI, confidence interval.

**Table S3. Age-standardised mortality rates and DALY rates in 1990 and 2021, and AAPC from 1990 to 2021 for the interpersonal violence burden attributable to IPV among women of childbearing age , by SDI quintile, region level.and country.**

| **Location** | **Mortality** | | | | | **DALYs** | | | | |
| --- | --- | --- | --- | --- | --- | --- | --- | --- | --- | --- |
|  | **Mortality cases in 1990 (95% UI)** | **ASMR in 1990 (95% UI)** | **Mortality cases in 2021 (95% UI)** | **ASMR in 2021 (95% UI)** | **AAPC% (95%CI),**  **1990–2021** | **DALYs cases in 1990 (95% UI)** | **ASDR in 1990 (95% UI)** | **DALYs cases in 2021 (95% UI)** | **ASDR in 2021 (95% UI)** | **AAPC% (95%CI),**  **1990–2021** |
| **Global** | 18814(11723 to 27306) | 1.40(0.87 to 2.02) | 18057(11148 to 26107) | 0.93(0.57 to 1.35) | -1.30(-1.67 to -0.92) | 1778645(1099269 to 2663965) | 130.17(80.93 to 194.28) | 1820958(1119982 to 2721916) | 94.23(57.84 to 140.99) | -1.03(-1.24 to -0.83) |
| **Age** | | | | | | | | | | |
| **15-19 years** | 3067(1854 to 4550) | 1.20(0.73 to 1.78) | 2818(1673 to 4182) | 0.93(0.55 to 1.38) | -0.84(-1.23 to -0.44) | 330699(197702 to 501794) | 129.41(77.37 to 196.37) | 306240(184400 to 455999) | 100.85(60.73 to 150.17) | -0.80(-1.12 to -0.47) |
| **20-24 years** | 3850(2101 to 5910) | 1.58(0.86 to 2.42) | 3508(1883 to 5298) | 1.19(0.64 to 1.80) | -0.89(-1.26 to -0.52) | 397999(217311 to 635897) | 163.02(89.01 to 260.47) | 366950(200995 to 583528) | 124.92(68.42 to 198.65) | -0.87(-1.11 to -0.62) |
| **25-29 years** | 3456(2168 to 5040) | 1.57(0.99 to 2.29) | 2992(1864 to 4335) | 1.03(0.64 to 1.49) | -1.34(-1.66 to -1.02) | 334198(210209 to 496612) | 151.84(95.51 to 225.63) | 315364(194199 to 472152) | 108.38(66.74 to 162.26) | -1.07(-1.26 to -0.89) |
| **30-34 years** | 3005(2013 to 4193) | 1.58(1.06 to 2.21) | 2823(1883 to 3959) | 0.94(0.63 to 1.32) | -1.66(-2.11 to -1.22) | 267444(178616 to 381628) | 140.68(93.95 to 200.74) | 291235(192558 to 421763) | 97.43(64.42 to 141.09) | -1.20(-1.49 to -0.91) |
| **35-39 years** | 2442(1665 to 3354) | 1.41(0.96 to 1.93) | 2354(1585 to 3294) | 0.85(0.57 to 1.19) | -1.62(-1.99 to -1.25) | 210919(145621 to 299579) | 121.60(83.95 to 172.71) | 228634(153742 to 327710) | 82.30(55.34 to 117.97) | -1.26(-1.46 to -1.06) |
| **35-39 years** | 1752(1145 to 2449) | 1.25(0.82 to 1.75) | 1936(1244 to 2689) | 0.78(0.50 to 1.08) | -1.50(-1.84 to -1.16) | 141801(91841 to 205264) | 101.12(65.50 to 146.38) | 173039(110357 to 253415) | 69.75(44.48 to 102.15) | -1.17(-1.37 to -0.97) |
| **45-49 years** | 1240(777 to 1811) | 1.09(0.68 to 1.59) | 1626(1015 to 2350) | 0.69(0.43 to 1.00) | -1.43(-1.69 to -1.16) | 95585(57969 to 143193) | 83.99(50.94 to 125.83) | 139496(83732 to 207348) | 59.20(35.53 to 87.99) | -1.11(-1.31 to -0.91) |
| **SDI regions** | | | | | | | | | | |
| **High SDI** | 2765(1751 to 4031) | 1.21(0.76 to 1.77) | 1749(1086 to 2569) | 0.72(0.44 to 1.06) | -1.70(-2.02 to -1.37) | 219268(138411 to 326321) | 97.18(61.01 to 145.04) | 159663(98878 to 239059) | 67.44(41.34 to 101.68) | -1.19(-1.40 to -0.97) |
| **High-middle SDI** | 3611(2275 to 5376) | 1.30(0.82 to 1.93) | 1654(1056 to 2382) | 0.53(0.34 to 0.77) | -2.80(-3.63 to -1.95) | 374991(233902 to 570220) | 133.65(83.50 to 202.87) | 234968(143189 to 363214) | 78.13(47.14 to 121.37) | -1.69(-2.17 to -1.21) |
| **Middle SDI** | 7443(4650 to 10681) | 1.65(1.04 to 2.36) | 5554(3478 to 7959) | 0.91(0.56 to 1.30) | -1.89(-2.26 to -1.53) | 704609(435195 to 1055312) | 153.39(95.64 to 228.85) | 561912(347571 to 839909) | 92.17(56.78 to 137.99) | -1.63(-1.82 to -1.45) |
| **Low-middle SDI** | 3092(1810 to 4744) | 1.10(0.65 to 1.68) | 4616(2755 to 6956) | 0.90(0.54 to 1.36) | -0.64(-1.14 to -0.14) | 303722(180896 to 470278) | 107.15(64.44 to 164.75) | 455110(275834 to 695938) | 88.55(53.82 to 135.12) | -0.63(-0.96 to -0.31) |
| **Low SDI** | 1879(1088 to 2913) | 1.64(0.96 to 2.54) | 4451(2533 to 6815) | 1.57(0.91 to 2.39) | -0.06(-0.99 to 0.89) | 174189(102439 to 264311) | 149.30(88.77 to 225.73) | 406738(241296 to 620749) | 141.57(84.89 to 214.66) | -0.11(-0.52 to 0.30) |
| **Regions** | | | | | | | | | | |
| **Andean Latin America** | 183(109 to 273) | 1.89(1.14 to 2.83) | 225(130 to 352) | 1.28(0.74 to 2.01) | -1.38(-2.28 to -0.46) | 14434(8784 to 21107) | 146.94(89.90 to 214.98) | 19013(11589 to 28240) | 108.47(66.05 to 161.28) | -0.96(-1.48 to -0.45) |
| **Australasia** | 56(35 to 83) | 1.04(0.64 to 1.54) | 31(19 to 46) | 0.42(0.26 to 0.63) | -2.98(-4.80 to -1.14) | 4445(2775 to 6596) | 83.24(51.73 to 123.89) | 3192(1956 to 4822) | 45.12(27.27 to 68.71) | -2.04(-3.28 to -0.79) |
| **Caribbean** | 418(235 to 658) | 4.32(2.46 to 6.79) | 642(335 to 1061) | 5.35(2.79 to 8.85) | 0.73(0.38 to 1.09) | 29302(16635 to 45713) | 298.37(171.05 to 465.00) | 43290(23174 to 69857) | 362.25(193.56 to 584.90) | 0.66(0.35 to 0.98) |
| **Central Asia** | 215(130 to 336) | 1.33(0.81 to 2.06) | 107(62 to 168) | 0.43(0.25 to 0.68) | -3.66(-4.47 to -2.86) | 15888(9513 to 25097) | 95.42(57.61 to 149.46) | 9416(5487 to 14942) | 38.33(22.19 to 61.12) | -3.05(-3.73 to -2.36) |
| **Central Europe** | 250(163 to 361) | 0.80(0.52 to 1.16) | 64(40 to 94) | 0.24(0.15 to 0.36) | -4.06(-4.34 to -3.78) | 21507(13949 to 31407) | 69.78(45.00 to 102.32) | 8363(5236 to 12541) | 32.77(20.32 to 49.64) | -2.55(-2.76 to -2.33) |
| **Central Latin America** | 406(246 to 636) | 0.94(0.57 to 1.46) | 891(517 to 1413) | 1.31(0.76 to 2.08) | 1.22(0.13 to 2.33) | 28757(17315 to 45643) | 64.87(39.39 to 102.04) | 59166(34655 to 94180) | 87.24(51.11 to 138.90) | 1.10(0.10 to 2.11) |
| **Central Sub-Saharan Africa** | 130(64 to 231) | 1.03(0.51 to 1.81) | 379(185 to 682) | 1.12(0.55 to 2.00) | -0.16(-1.09 to 0.77) | 16480(9052 to 27012) | 126.69(70.40 to 206.84) | 44504(24407 to 72837) | 129.57(71.68 to 211.18) | -0.09(-0.62 to 0.45) |
| **East Asia** | 3894(2208 to 6186) | 1.17(0.67 to 1.85) | 1062(597 to 1681) | 0.32(0.18 to 0.51) | -4.19(-4.45 to -3.92) | 534456(317239 to 852957) | 157.56(94.19 to 250.45) | 290171(161855 to 479145) | 90.56(49.98 to 149.78) | -1.76(-1.90 to -1.62) |
| **Eastern Europe** | 1268(773 to 1960) | 2.24(1.36 to 3.48) | 646(391 to 985) | 1.22(0.73 to 1.90) | -1.98(-3.29 to -0.65) | 87655(52822 to 136494) | 155.90(93.13 to 243.91) | 43299(26342 to 65925) | 85.66(51.37 to 132.45) | -1.85(-3.07 to -0.62) |
| **Eastern Sub-Saharan Africa** | 926(511 to 1510) | 2.09(1.16 to 3.40) | 2060(1143 to 3319) | 1.85(1.04 to 2.95) | -0.36(-0.91 to 0.19) | 83888(47511 to 131735) | 183.95(105.56 to 287.24) | 186019(106809 to 293571) | 164.04(95.69 to 256.96) | -0.32(-0.73 to 0.09) |
| **High-income Asia Pacific** | 120(70 to 190) | 0.26(0.15 to 0.42) | 54(31 to 87) | 0.14(0.08 to 0.23) | -1.92(-2.53 to -1.31) | 13572(7799 to 21911) | 30.08(17.23 to 48.72) | 7433(4211 to 12070) | 20.48(11.41 to 33.85) | -1.18(-1.45 to -0.91) |
| **High-income North America** | 1768(1100 to 2671) | 2.36(1.46 to 3.59) | 1158(709 to 1755) | 1.38(0.84 to 2.10) | -1.76(-2.19 to -1.34) | 118809(73417 to 181833) | 160.55(98.26 to 247.43) | 80799(48944 to 124446) | 97.82(58.87 to 151.57) | -1.62(-1.99 to -1.25) |
| **North Africa and Middle East** | 816(513 to 1137) | 1.06(0.67 to 1.48) | 1744(1081 to 2485) | 1.09(0.68 to 1.56) | -0.02(-0.62 to 0.59) | 92081(58863 to 130491) | 117.18(75.47 to 166.06) | 187025(119204 to 264501) | 117.49(74.72 to 166.26) | -0.09(-0.44 to 0.25) |
| **Oceania** | 29(14 to 56) | 1.89(0.88 to 3.56) | 117(52 to 225) | 3.34(1.47 to 6.37) | 1.78(1.04 to 2.52) | 3070(1653 to 5225) | 191.96(104.62 to 324.86) | 10335(5260 to 17760) | 292.34(149.20 to 501.22) | 1.33(0.92 to 1.75) |
| **South Asia** | 2599(1461 to 4186) | 0.99(0.56 to 1.59) | 3031(1706 to 4814) | 0.61(0.34 to 0.97) | -1.42(-1.87 to -0.97) | 265905(152314 to 430123) | 100.59(58.14 to 161.43) | 341258(194733 to 555005) | 68.30(39.08 to 110.83) | -1.22(-1.44 to -1.01) |
| **Southeast Asia** | 1351(791 to 2047) | 1.10(0.65 to 1.66) | 1012(605 to 1532) | 0.55(0.33 to 0.84) | -2.19(-2.51 to -1.87) | 117970(70342 to 179856) | 94.74(57.04 to 143.24) | 104648(62795 to 160644) | 57.56(34.43 to 88.52) | -1.62(-1.82 to -1.43) |
| **Southern Latin America** | 132(88 to 191) | 1.06(0.71 to 1.55) | 143(84 to 223) | 0.82(0.48 to 1.28) | -0.89(-1.53 to -0.24) | 10153(6738 to 14987) | 81.59(54.29 to 120.05) | 11363(6748 to 17763) | 66.01(39.00 to 103.67) | -0.69(-1.15 to -0.23) |
| **Southern Sub-Saharan Africa** | 2063(1278 to 2982) | 15.44(9.62 to 22.47) | 1299(742 to 1965) | 5.96(3.41 to 9.03) | -3.02(-3.91 to -2.11) | 136118(85040 to 194351) | 997.43(626.77 to 1432.07) | 86592(50506 to 129410) | 396.03(230.75 to 592.24) | -2.93(-3.75 to -2.11) |
| **Tropical Latin America** | 863(493 to 1406) | 2.09(1.21 to 3.39) | 1126(653 to 1810) | 1.90(1.10 to 3.07) | -0.28(-0.71 to 0.16) | 61241(34999 to 100825) | 146.27(84.24 to 239.31) | 77525(44676 to 125840) | 132.58(75.93 to 216.15) | -0.37(-0.96 to 0.22) |
| **Western Europe** | 591(389 to 818) | 0.61(0.40 to 0.85) | 231(149 to 321) | 0.24(0.16 to 0.34) | -2.89(-3.63 to -2.16) | 58256(37620 to 83538) | 61.43(39.56 to 88.24) | 34117(21072 to 50872) | 38.25(23.33 to 57.48) | -1.52(-1.82 to -1.22) |
| **Western Sub-Saharan Africa** | 735(412 to 1135) | 1.67(0.95 to 2.59) | 2034(1038 to 3464) | 1.66(0.86 to 2.81) | 0.00(-0.62 to 0.63) | 64655(37919 to 96753) | 143.16(85.01 to 214.02) | 173430(96069 to 278412) | 138.32(77.56 to 220.40) | -0.21(-0.71 to 0.29) |
| **Countries** | | | | | | | | | | |
| **Afghanistan** | 82(36 to 152) | 4.06(1.81 to 7.57) | 258(114 to 497) | 3.76(1.67 to 7.27) | -0.70(-2.40 to 1.03) | 6458(3385 to 10930) | 312.43(165.98 to 529.35) | 21325(11285 to 36499) | 301.17(161.56 to 515.56) | -0.26(-0.47 to -0.06) |
| **Albania** | 9(5 to 16) | 1.06(0.54 to 1.85) | 3(1 to 6) | 0.46(0.21 to 0.90) | -2.80(-3.75 to -1.84) | 738(403 to 1260) | 86.33(47.73 to 146.11) | 265(141 to 470) | 43.12(22.89 to 76.35) | -2.35(-3.05 to -1.64) |
| **Algeria** | 36(16 to 68) | 0.64(0.29 to 1.19) | 41(18 to 77) | 0.36(0.16 to 0.68) | -1.32(-1.91 to -0.72) | 5353(3084 to 8247) | 91.91(53.70 to 141.14) | 7789(4496 to 12231) | 69.08(39.53 to 108.62) | -0.84(-1.04 to -0.65) |
| **American Samoa** | 0(0 to 0) | 1.16(0.49 to 2.28) | 0(0 to 0) | 0.94(0.41 to 1.87) | -0.64(-1.39 to 0.13) | 18(10 to 31) | 143.76(77.15 to 245.13) | 15(8 to 24) | 126.99(68.50 to 212.01) | -0.38(-0.73 to -0.02) |
| **Andorra** | 0(0 to 0) | 0.01(0.00 to 0.01) | 0(0 to 0) | 0.00(0.00 to 0.01) | -2.62(-2.94 to -2.31) | 3(2 to 5) | 20.47(11.02 to 34.17) | 4(2 to 6) | 20.67(11.17 to 34.36) | 0.03(0.01 to 0.05) |
| **Angola** | 26(10 to 51) | 1.07(0.44 to 2.13) | 76(29 to 162) | 0.95(0.37 to 2.01) | -0.38(-0.93 to 0.18) | 2942(1548 to 4974) | 121.15(64.40 to 203.68) | 8964(4594 to 15818) | 110.53(57.19 to 193.59) | -0.29(-0.58 to 0.01) |
| **Antigua and Barbuda** | 0(0 to 0) | 1.59(0.90 to 2.49) | 0(0 to 0) | 1.01(0.56 to 1.60) | -1.39(-3.21 to 0.45) | 21(12 to 32) | 122.13(70.06 to 189.39) | 20(11 to 31) | 86.16(48.98 to 134.11) | -1.13(-2.52 to 0.28) |
| **Argentina** | 83(55 to 119) | 1.03(0.69 to 1.49) | 109(62 to 175) | 0.92(0.52 to 1.48) | -0.35(-1.47 to 0.78) | 6349(4260 to 9127) | 79.04(53.15 to 113.45) | 8613(4962 to 13781) | 73.27(42.04 to 117.65) | -0.25(-1.08 to 0.59) |
| **Armenia** | 6(3 to 9) | 0.66(0.39 to 1.04) | 1(1 to 2) | 0.14(0.08 to 0.22) | -5.15(-6.10 to -4.18) | 466(272 to 741) | 52.34(30.63 to 82.87) | 145(84 to 233) | 19.26(10.99 to 31.28) | -3.26(-4.02 to -2.49) |
| **Australia** | 47(29 to 71) | 1.05(0.64 to 1.59) | 25(15 to 37) | 0.40(0.24 to 0.61) | -3.30(-5.56 to -0.99) | 3681(2276 to 5528) | 82.94(51.01 to 125.03) | 2563(1555 to 3929) | 43.39(25.92 to 67.21) | -2.24(-3.74 to -0.72) |
| **Austria** | 19(11 to 26) | 0.93(0.57 to 1.30) | 8(5 to 11) | 0.38(0.23 to 0.54) | -2.68(-3.91 to -1.43) | 1577(992 to 2217) | 79.76(50.08 to 112.11) | 976(608 to 1391) | 50.93(31.34 to 72.78) | -1.36(-1.97 to -0.74) |
| **Azerbaijan** | 19(10 to 33) | 1.02(0.56 to 1.72) | 9(4 to 16) | 0.31(0.14 to 0.58) | -3.65(-4.08 to -3.22) | 1550(874 to 2573) | 80.61(45.87 to 132.10) | 897(480 to 1529) | 32.64(17.28 to 56.12) | -2.93(-3.14 to -2.73) |
| **Bahamas** | 3(2 to 5) | 4.36(2.46 to 6.80) | 6(3 to 9) | 5.30(2.82 to 8.67) | 0.84(-1.14 to 2.86) | 216(123 to 333) | 288.75(165.17 to 444.59) | 370(201 to 596) | 352.23(190.89 to 568.09) | 0.82(-1.03 to 2.70) |
| **Bahrain** | 1(0 to 1) | 0.64(0.30 to 1.20) | 2(1 to 4) | 0.71(0.31 to 1.33) | 0.28(-0.54 to 1.11) | 108(62 to 169) | 90.52(52.72 to 143.01) | 291(163 to 461) | 89.39(49.91 to 141.60) | -0.09(-0.58 to 0.39) |
| **Bangladesh** | 200(97 to 368) | 0.75(0.36 to 1.37) | 186(77 to 375) | 0.40(0.17 to 0.80) | -1.91(-2.30 to -1.53) | 26381(14423 to 44796) | 99.48(55.07 to 167.45) | 32557(17081 to 57521) | 69.70(36.67 to 122.96) | -1.10(-1.28 to -0.91) |
| **Barbados** | 3(1 to 4) | 3.77(2.13 to 5.86) | 2(1 to 4) | 3.60(1.86 to 5.93) | -0.03(-1.87 to 1.85) | 185(105 to 283) | 263.24(149.29 to 402.38) | 166(89 to 267) | 248.50(132.55 to 399.92) | -0.08(-1.71 to 1.59) |
| **Belarus** | 32(19 to 51) | 1.25(0.72 to 2.00) | 13(7 to 20) | 0.51(0.28 to 0.83) | -2.70(-4.66 to -0.71) | 2240(1290 to 3576) | 87.08(49.91 to 139.53) | 890(506 to 1422) | 38.48(21.57 to 62.23) | -2.48(-4.03 to -0.90) |
| **Belgium** | 26(16 to 39) | 1.06(0.64 to 1.58) | 13(8 to 20) | 0.52(0.31 to 0.76) | -2.45(-2.90 to -2.01) | 2037(1243 to 2990) | 83.63(50.66 to 122.97) | 1221(746 to 1802) | 49.43(29.89 to 73.11) | -1.79(-2.08 to -1.50) |
| **Belize** | 1(1 to 2) | 2.43(1.35 to 3.80) | 4(2 to 6) | 2.96(1.65 to 4.67) | 0.97(0.06 to 1.88) | 82(46 to 127) | 178.15(101.84 to 276.22) | 253(144 to 391) | 206.68(117.51 to 319.17) | 0.76(0.00 to 1.53) |
| **Benin** | 15(6 to 29) | 1.30(0.54 to 2.53) | 55(21 to 121) | 1.61(0.63 to 3.55) | 0.72(0.42 to 1.02) | 1363(694 to 2377) | 117.98(60.77 to 204.14) | 4661(2195 to 9099) | 135.13(64.61 to 259.88) | 0.46(0.27 to 0.66) |
| **Bermuda** | 0(0 to 0) | 1.36(0.75 to 2.14) | 0(0 to 0) | 0.82(0.43 to 1.33) | -1.58(-2.96 to -0.18) | 18(11 to 29) | 108.28(61.93 to 168.76) | 9(5 to 14) | 73.22(40.43 to 115.65) | -1.38(-2.35 to -0.41) |
| **Bhutan** | 1(0 to 1) | 0.44(0.17 to 0.93) | 0(0 to 1) | 0.22(0.09 to 0.47) | -2.32(-2.72 to -1.92) | 100(52 to 184) | 66.06(34.53 to 118.65) | 102(53 to 180) | 48.26(25.24 to 85.49) | -1.02(-1.16 to -0.88) |
| **Bolivia (Plurinational State of)** | 43(19 to 81) | 2.75(1.18 to 5.18) | 54(22 to 106) | 1.71(0.71 to 3.38) | -1.57(-1.91 to -1.23) | 3419(1696 to 5960) | 214.77(107.01 to 373.50) | 4603(2257 to 8198) | 145.96(71.79 to 259.45) | -1.34(-1.57 to -1.11) |
| **Bosnia and Herzegovina** | 3(1 to 6) | 0.24(0.10 to 0.50) | 1(0 to 2) | 0.11(0.04 to 0.21) | -2.69(-3.18 to -2.20) | 421(232 to 722) | 36.04(19.86 to 61.84) | 171(96 to 285) | 23.61(13.16 to 39.66) | -1.38(-1.58 to -1.19) |
| **Botswana** | 1(0 to 2) | 0.31(0.14 to 0.58) | 15(6 to 31) | 2.25(0.90 to 4.48) | 6.58(5.06 to 8.12) | 191(112 to 296) | 56.32(33.10 to 87.65) | 1241(620 to 2224) | 181.38(90.46 to 325.69) | 3.79(2.62 to 4.98) |
| **Brazil** | 854(488 to 1392) | 2.12(1.22 to 3.43) | 1105(641 to 1777) | 1.93(1.11 to 3.12) | -0.28(-0.72 to 0.16) | 60557(34576 to 99704) | 148.15(85.23 to 242.41) | 75995(43746 to 123586) | 134.44(76.88 to 219.66) | -0.28(-0.68 to 0.12) |
| **Brunei Darussalam** | 0(0 to 0) | 0.32(0.14 to 0.60) | 0(0 to 0) | 0.14(0.06 to 0.28) | -2.47(-2.67 to -2.27) | 22(11 to 38) | 31.47(16.11 to 55.25) | 24(13 to 42) | 19.51(10.45 to 34.33) | -1.51(-1.61 to -1.40) |
| **Bulgaria** | 5(3 to 9) | 0.26(0.15 to 0.42) | 2(1 to 3) | 0.11(0.06 to 0.18) | -3.03(-3.62 to -2.44) | 464(272 to 742) | 22.76(13.25 to 36.65) | 175(103 to 281) | 12.64(7.29 to 20.64) | -2.03(-2.46 to -1.59) |
| **Burkina Faso** | 62(26 to 122) | 2.97(1.26 to 5.83) | 147(61 to 298) | 2.61(1.09 to 5.31) | -0.34(-0.66 to -0.03) | 4456(2123 to 8324) | 207.79(100.07 to 386.00) | 10876(4986 to 20558) | 187.99(87.62 to 354.95) | -0.25(-0.53 to 0.02) |
| **Burundi** | 3(1 to 6) | 0.21(0.08 to 0.43) | 7(2 to 18) | 0.21(0.07 to 0.52) | -0.73(-2.32 to 0.89) | 784(411 to 1348) | 58.80(31.12 to 100.79) | 1873(959 to 3380) | 56.38(29.28 to 100.84) | -0.43(-1.04 to 0.18) |
| **Cabo Verde** | 3(1 to 5) | 3.38(1.51 to 6.26) | 4(2 to 8) | 2.64(1.22 to 5.01) | -0.88(-1.26 to -0.50) | 199(99 to 350) | 247.29(124.89 to 433.17) | 296(151 to 526) | 195.02(99.56 to 345.93) | -0.84(-1.15 to -0.52) |
| **Cambodia** | 35(16 to 68) | 1.33(0.60 to 2.58) | 34(13 to 77) | 0.74(0.28 to 1.68) | -1.85(-2.13 to -1.56) | 2886(1456 to 5209) | 109.46(55.94 to 195.92) | 3040(1436 to 5985) | 66.57(31.48 to 130.81) | -1.58(-1.78 to -1.38) |
| **Cameroon** | 24(10 to 48) | 0.98(0.41 to 1.93) | 68(25 to 158) | 0.82(0.31 to 1.89) | -0.85(-1.20 to -0.50) | 2669(1356 to 4568) | 105.61(54.48 to 180.06) | 7803(3906 to 14494) | 93.73(47.53 to 172.81) | -0.46(-1.16 to 0.24) |
| **Canada** | 72(45 to 109) | 0.97(0.60 to 1.49) | 33(19 to 54) | 0.40(0.23 to 0.65) | -2.94(-4.24 to -1.62) | 5313(3291 to 8209) | 73.11(44.63 to 114.53) | 3039(1764 to 4914) | 38.11(21.82 to 62.42) | -2.17(-3.59 to -0.73) |
| **Central African Republic** | 11(5 to 21) | 1.70(0.72 to 3.33) | 28(11 to 56) | 1.95(0.81 to 3.92) | 0.67(-5.15 to 6.85) | 1009(518 to 1757) | 152.17(78.71 to 264.64) | 2468(1231 to 4408) | 171.69(86.34 to 304.31) | 0.59(-4.17 to 5.58) |
| **Chad** | 17(7 to 35) | 1.29(0.53 to 2.58) | 81(36 to 160) | 2.02(0.91 to 3.98) | 1.46(-0.37 to 3.32) | 1645(824 to 2902) | 119.71(60.84 to 209.82) | 6791(3418 to 12167) | 164.69(84.49 to 292.19) | 1.04(-0.26 to 2.36) |
| **Chile** | 40(23 to 64) | 1.12(0.65 to 1.78) | 25(14 to 40) | 0.52(0.29 to 0.84) | -2.21(-2.72 to -1.70) | 3142(1851 to 5031) | 86.09(50.97 to 137.29) | 2085(1196 to 3366) | 44.81(25.51 to 72.81) | -1.88(-2.29 to -1.47) |
| **China** | 3766(2125 to 6006) | 1.17(0.67 to 1.86) | 981(540 to 1578) | 0.31(0.17 to 0.50) | -4.28(-4.56 to -4.00) | 516508(306026 to 827517) | 157.50(94.01 to 251.34) | 275743(152541 to 456896) | 89.41(48.91 to 148.35) | -1.80(-1.95 to -1.66) |
| **Colombia** | 94(53 to 151) | 1.05(0.59 to 1.67) | 55(28 to 93) | 0.42(0.21 to 0.70) | -2.88(-3.96 to -1.78) | 6209(3469 to 10016) | 67.54(38.09 to 108.28) | 3727(1939 to 6183) | 28.46(14.78 to 47.20) | -2.92(-4.08 to -1.74) |
| **Comoros** | 2(1 to 5) | 2.10(0.70 to 4.36) | 6(3 to 12) | 3.07(1.28 to 5.93) | 1.16(-1.69 to 4.08) | 176(70 to 345) | 156.45(64.19 to 302.98) | 432(195 to 807) | 214.55(97.32 to 398.83) | 0.94(-1.62 to 3.57) |
| **Congo** | 8(3 to 18) | 1.55(0.55 to 3.22) | 25(10 to 52) | 1.71(0.66 to 3.59) | 0.31(-0.74 to 1.37) | 849(418 to 1528) | 147.42(72.77 to 264.79) | 2266(1109 to 4098) | 155.71(76.20 to 281.51) | 0.17(-0.53 to 0.87) |
| **Cook Islands** | 0(0 to 0) | 1.05(0.46 to 2.07) | 0(0 to 0) | 0.35(0.15 to 0.68) | -3.56(-3.84 to -3.28) | 6(3 to 11) | 133.67(72.46 to 226.40) | 4(2 to 6) | 87.66(46.96 to 148.24) | -1.36(-1.45 to -1.26) |
| **Costa Rica** | 11(6 to 16) | 1.34(0.80 to 2.02) | 22(12 to 35) | 1.66(0.92 to 2.69) | 0.61(-0.19 to 1.41) | 825(492 to 1231) | 102.64(61.60 to 152.65) | 1560(898 to 2485) | 121.12(69.50 to 193.23) | 0.49(-0.15 to 1.13) |
| **Coted'Ivoire** | 34(14 to 72) | 1.19(0.48 to 2.51) | 105(40 to 225) | 1.53(0.58 to 3.25) | 0.74(-0.01 to 1.49) | 3181(1580 to 5802) | 109.21(55.26 to 196.96) | 8986(4285 to 16938) | 128.86(62.02 to 241.61) | 0.48(-0.05 to 1.02) |
| **Croatia** | 15(9 to 21) | 1.20(0.76 to 1.70) | 3(2 to 5) | 0.34(0.21 to 0.49) | -4.13(-5.16 to -3.10) | 1366(876 to 1900) | 112.54(71.91 to 156.50) | 452(279 to 643) | 49.81(30.73 to 70.71) | -2.71(-3.29 to -2.12) |
| **Cuba** | 108(61 to 160) | 3.36(1.91 to 5.01) | 46(25 to 73) | 1.85(1.01 to 2.95) | -2.07(-3.10 to -1.03) | 7679(4369 to 11451) | 238.12(136.77 to 354.56) | 3237(1821 to 5074) | 134.72(75.23 to 210.12) | -2.04(-2.81 to -1.26) |
| **Cyprus** | 2(1 to 4) | 1.00(0.43 to 1.93) | 1(1 to 2) | 0.32(0.15 to 0.57) | -3.50(-3.97 to -3.02) | 171(89 to 295) | 86.55(44.87 to 149.50) | 151(86 to 242) | 43.17(24.01 to 70.39) | -2.12(-2.55 to -1.69) |
| **Czechia** | 34(22 to 45) | 1.28(0.84 to 1.70) | 9(5 to 12) | 0.35(0.22 to 0.47) | -4.31(-4.93 to -3.68) | 3145(2084 to 4193) | 121.83(80.95 to 162.08) | 1538(988 to 2141) | 65.96(42.41 to 92.09) | -2.04(-2.42 to -1.65) |
| **Democratic People's Republic of Korea** | 68(28 to 137) | 1.21(0.50 to 2.42) | 60(24 to 125) | 0.90(0.36 to 1.87) | -0.91(-1.09 to -0.72) | 9459(5139 to 15544) | 165.96(90.66 to 271.88) | 8765(4711 to 14448) | 133.63(71.65 to 220.37) | -0.70(-0.80 to -0.61) |
| **Democratic Republic of the Congo** | 82(33 to 163) | 0.92(0.38 to 1.84) | 241(104 to 475) | 1.09(0.47 to 2.13) | -0.50(-1.75 to 0.78) | 11263(5889 to 19124) | 125.00(66.26 to 210.83) | 29803(15835 to 50725) | 132.52(71.07 to 224.24) | -0.23(-0.93 to 0.48) |
| **Denmark** | 14(9 to 19) | 1.05(0.69 to 1.44) | 4(3 to 6) | 0.32(0.20 to 0.45) | -3.87(-4.31 to -3.43) | 1138(746 to 1554) | 87.27(57.04 to 118.97) | 526(331 to 753) | 42.42(26.61 to 60.79) | -2.40(-2.73 to -2.07) |
| **Djibouti** | 1(1 to 3) | 1.31(0.52 to 2.69) | 7(3 to 14) | 2.03(0.79 to 4.33) | 0.98(0.05 to 1.93) | 132(65 to 237) | 129.26(65.32 to 228.98) | 544(265 to 1030) | 169.23(81.96 to 320.70) | 0.59(-0.05 to 1.24) |
| **Dominica** | 0(0 to 0) | 1.50(0.76 to 2.59) | 0(0 to 1) | 2.15(0.90 to 4.08) | 0.78(0.37 to 1.20) | 21(11 to 34) | 118.26(64.09 to 192.82) | 26(12 to 48) | 159.82(73.37 to 290.71) | 0.65(0.33 to 0.98) |
| **Dominican Republic** | 53(26 to 88) | 2.69(1.36 to 4.50) | 113(52 to 206) | 3.85(1.77 to 7.04) | 0.94(0.28 to 1.61) | 3873(2018 to 6329) | 192.61(101.94 to 313.91) | 7674(3724 to 13620) | 261.46(126.93 to 464.12) | 0.81(0.24 to 1.38) |
| **Ecuador** | 45(27 to 69) | 1.78(1.06 to 2.71) | 69(37 to 114) | 1.46(0.77 to 2.39) | -0.82(-1.27 to -0.37) | 3524(2089 to 5467) | 135.73(81.34 to 209.25) | 5615(3156 to 9004) | 117.80(66.38 to 188.80) | -0.68(-1.06 to -0.30) |
| **Egypt** | 26(15 to 42) | 0.20(0.12 to 0.32) | 96(48 to 167) | 0.37(0.18 to 0.64) | 1.97(1.20 to 2.74) | 7415(4465 to 11250) | 56.64(34.31 to 85.73) | 15931(9292 to 24733) | 60.98(35.66 to 94.58) | 0.21(-0.18 to 0.60) |
| **El Salvador** | 12(6 to 21) | 0.94(0.49 to 1.63) | 23(11 to 43) | 1.31(0.63 to 2.41) | 1.16(-1.94 to 4.37) | 802(421 to 1407) | 60.10(31.97 to 103.93) | 1503(747 to 2756) | 83.91(41.81 to 153.33) | 1.18(-1.84 to 4.28) |
| **Equatorial Guinea** | 1(1 to 3) | 1.41(0.55 to 2.88) | 4(1 to 11) | 1.15(0.36 to 2.92) | -0.59(-1.23 to 0.05) | 144(72 to 250) | 141.97(71.03 to 245.09) | 457(211 to 942) | 119.93(55.93 to 245.54) | -0.52(-0.89 to -0.14) |
| **Eritrea** | 15(6 to 30) | 1.88(0.80 to 3.82) | 54(22 to 105) | 3.22(1.30 to 6.29) | 1.72(1.51 to 1.92) | 1315(667 to 2386) | 161.84(83.23 to 291.71) | 4188(1928 to 7610) | 245.57(114.14 to 444.47) | 1.32(1.14 to 1.50) |
| **Estonia** | 3(2 to 5) | 0.85(0.49 to 1.35) | 1(0 to 1) | 0.22(0.12 to 0.35) | -4.59(-5.60 to -3.56) | 248(145 to 392) | 64.75(37.45 to 103.03) | 61(35 to 98) | 22.21(12.46 to 36.25) | -3.59(-4.20 to -2.98) |
| **Eswatini** | 18(9 to 29) | 8.57(4.45 to 14.15) | 15(5 to 32) | 4.52(1.61 to 9.86) | -2.10(-2.39 to -1.81) | 1253(679 to 1983) | 591.06(323.28 to 941.57) | 1045(432 to 2107) | 317.63(132.61 to 640.50) | -2.05(-2.33 to -1.78) |
| **Ethiopia** | 442(192 to 828) | 3.88(1.68 to 7.27) | 658(342 to 1128) | 2.30(1.21 to 3.90) | -1.77(-2.24 to -1.29) | 34563(16788 to 60845) | 294.39(144.07 to 516.95) | 53137(29385 to 87465) | 181.67(101.90 to 295.43) | -1.64(-2.02 to -1.26) |
| **Fiji** | 2(1 to 4) | 1.12(0.53 to 2.04) | 3(1 to 5) | 1.19(0.50 to 2.40) | 0.19(-0.25 to 0.64) | 287(160 to 467) | 143.03(80.22 to 232.12) | 336(178 to 575) | 147.16(77.75 to 252.04) | 0.08(-0.16 to 0.32) |
| **Finland** | 21(13 to 29) | 1.60(1.02 to 2.25) | 8(5 to 11) | 0.69(0.44 to 0.97) | -2.59(-4.15 to -1.01) | 1612(1041 to 2238) | 128.88(82.52 to 179.38) | 799(519 to 1136) | 72.58(46.64 to 103.25) | -1.65(-2.97 to -0.32) |
| **France** | 102(62 to 148) | 0.69(0.42 to 1.01) | 31(18 to 46) | 0.21(0.13 to 0.31) | -3.83(-5.01 to -2.63) | 9316(5690 to 13645) | 64.44(39.18 to 94.56) | 4672(2781 to 7130) | 33.74(19.92 to 51.58) | -2.13(-2.78 to -1.47) |
| **Gabon** | 2(1 to 5) | 1.13(0.41 to 2.26) | 5(2 to 12) | 1.02(0.38 to 2.38) | -0.31(-0.50 to -0.12) | 273(136 to 467) | 118.17(59.82 to 201.89) | 546(278 to 1040) | 108.31(55.28 to 205.73) | -0.29(-0.42 to -0.17) |
| **Gambia** | 0(0 to 1) | 0.10(0.04 to 0.21) | 1(0 to 2) | 0.13(0.05 to 0.27) | 0.58(-0.35 to 1.51) | 69(39 to 109) | 29.79(17.16 to 46.74) | 174(96 to 285) | 27.29(15.27 to 44.40) | -0.37(-0.71 to -0.03) |
| **Georgia** | 7(4 to 11) | 0.50(0.29 to 0.79) | 1(1 to 2) | 0.14(0.08 to 0.24) | -3.91(-7.04 to -0.68) | 562(328 to 893) | 40.36(23.51 to 64.05) | 142(83 to 225) | 17.72(10.18 to 28.36) | -2.59(-4.95 to -0.18) |
| **Germany** | 108(66 to 164) | 0.55(0.34 to 0.84) | 36(22 to 53) | 0.21(0.13 to 0.32) | -3.11(-4.49 to -1.72) | 10034(6025 to 15793) | 52.46(31.23 to 82.97) | 5477(3285 to 8494) | 33.60(19.79 to 52.86) | -1.45(-2.24 to -0.65) |
| **Ghana** | 38(16 to 75) | 1.07(0.45 to 2.09) | 119(50 to 246) | 1.29(0.54 to 2.65) | -0.06(-1.12 to 1.02) | 3746(1940 to 6400) | 101.81(53.37 to 173.43) | 10536(5241 to 19056) | 112.28(56.16 to 202.72) | -0.17(-0.99 to 0.66) |
| **Greece** | 12(7 to 18) | 0.49(0.29 to 0.72) | 6(4 to 9) | 0.26(0.15 to 0.38) | -2.26(-3.43 to -1.08) | 1425(855 to 2120) | 57.11(34.22 to 84.94) | 776(465 to 1186) | 37.55(22.15 to 57.55) | -1.49(-2.06 to -0.91) |
| **Greenland** | 2(1 to 3) | 11.72(5.90 to 21.94) | 1(0 to 1) | 4.12(2.00 to 7.44) | -3.29(-3.52 to -3.06) | 112(57 to 209) | 733.38(374.77 to 1358.31) | 34(17 to 60) | 259.84(130.58 to 466.58) | -3.28(-3.50 to -3.06) |
| **Grenada** | 0(0 to 1) | 2.04(1.11 to 3.21) | 0(0 to 1) | 1.26(0.67 to 2.05) | -1.99(-4.09 to 0.17) | 32(18 to 49) | 152.77(85.28 to 237.17) | 26(15 to 42) | 102.17(56.97 to 162.79) | -1.67(-3.39 to 0.07) |
| **Guam** | 1(0 to 1) | 1.52(0.65 to 3.02) | 0(0 to 1) | 0.94(0.47 to 1.73) | -1.70(-2.96 to -0.42) | 59(31 to 102) | 165.09(86.07 to 284.11) | 45(25 to 74) | 125.10(69.57 to 206.43) | -0.84(-1.36 to -0.32) |
| **Guatemala** | 18(10 to 31) | 0.97(0.52 to 1.63) | 32(16 to 55) | 0.71(0.36 to 1.21) | -1.59(-4.35 to 1.25) | 1198(632 to 2036) | 62.52(33.33 to 105.25) | 2166(1095 to 3696) | 47.01(23.96 to 79.65) | -1.44(-4.03 to 1.23) |
| **Guinea** | 20(8 to 39) | 1.43(0.58 to 2.83) | 66(27 to 138) | 1.92(0.79 to 3.98) | 0.91(0.72 to 1.10) | 1767(873 to 3113) | 125.50(62.54 to 220.48) | 5452(2659 to 10226) | 155.14(76.42 to 288.79) | 0.67(0.54 to 0.80) |
| **Guinea-Bissau** | 10(4 to 20) | 4.43(1.94 to 8.40) | 20(8 to 43) | 3.87(1.55 to 8.10) | -0.43(-0.53 to -0.33) | 756(363 to 1356) | 314.92(153.05 to 563.48) | 1493(679 to 2911) | 275.31(125.40 to 534.14) | -0.43(-0.50 to -0.36) |
| **Guyana** | 9(5 to 15) | 4.33(2.42 to 6.90) | 14(7 to 23) | 6.61(3.36 to 11.17) | 1.55(0.29 to 2.82) | 648(366 to 1016) | 297.19(169.64 to 465.64) | 907(468 to 1515) | 434.14(225.54 to 722.65) | 1.39(0.17 to 2.62) |
| **Haiti** | 141(50 to 282) | 8.75(3.13 to 17.51) | 316(125 to 607) | 8.75(3.47 to 16.85) | 0.27(-0.18 to 0.72) | 9785(3920 to 18971) | 596.49(240.83 to 1151.89) | 21653(9296 to 40433) | 597.37(256.51 to 1115.78) | 0.18(-0.39 to 0.75) |
| **Honduras** | 37(18 to 68) | 3.52(1.66 to 6.40) | 99(40 to 196) | 3.50(1.42 to 6.91) | -0.46(-2.98 to 2.13) | 2448(1183 to 4421) | 222.66(107.75 to 400.39) | 6246(2636 to 12261) | 216.94(92.10 to 423.94) | -0.52(-3.02 to 2.04) |
| **Hungary** | 31(19 to 47) | 1.17(0.71 to 1.81) | 7(4 to 11) | 0.31(0.18 to 0.48) | -4.18(-5.38 to -2.95) | 2643(1602 to 4055) | 103.54(62.21 to 160.03) | 929(550 to 1454) | 44.58(26.11 to 70.35) | -2.69(-3.22 to -2.16) |
| **Iceland** | 0(0 to 1) | 0.53(0.32 to 0.80) | 0(0 to 0) | 0.22(0.13 to 0.33) | -2.83(-4.10 to -1.54) | 36(22 to 54) | 55.38(33.26 to 82.41) | 26(15 to 40) | 33.05(19.25 to 51.23) | -1.65(-2.43 to -0.87) |
| **India** | 2160(1191 to 3538) | 1.05(0.58 to 1.71) | 2211(1224 to 3602) | 0.58(0.32 to 0.95) | -1.71(-2.25 to -1.18) | 212657(120737 to 344701) | 102.11(58.40 to 164.54) | 242653(137463 to 401276) | 63.70(36.16 to 105.08) | -1.42(-1.74 to -1.10) |
| **Indonesia** | 180(97 to 292) | 0.35(0.19 to 0.57) | 167(87 to 290) | 0.23(0.12 to 0.39) | -1.42(-2.57 to -0.26) | 24990(14254 to 39441) | 49.81(28.70 to 78.25) | 32052(18476 to 50719) | 43.17(24.81 to 68.45) | -0.47(-0.88 to -0.06) |
| **Iran (Islamic Republic of)** | 129(77 to 185) | 0.98(0.59 to 1.40) | 207(124 to 294) | 0.94(0.55 to 1.34) | -0.11(-0.57 to 0.36) | 14954(9288 to 21233) | 114.17(71.33 to 161.88) | 24220(15038 to 34453) | 108.44(66.40 to 154.41) | -0.15(-0.38 to 0.08) |
| **Iraq** | 85(45 to 138) | 2.15(1.15 to 3.49) | 377(177 to 667) | 3.60(1.70 to 6.38) | 1.93(0.74 to 3.12) | 7837(4550 to 11901) | 192.44(113.57 to 291.04) | 29505(15747 to 48173) | 278.59(149.60 to 454.51) | 1.33(0.49 to 2.18) |
| **Ireland** | 2(1 to 4) | 0.26(0.16 to 0.41) | 1(1 to 2) | 0.10(0.06 to 0.16) | -2.83(-4.11 to -1.53) | 257(153 to 412) | 29.00(17.32 to 46.35) | 211(122 to 346) | 18.98(10.80 to 31.46) | -1.41(-1.59 to -1.22) |
| **Israel** | 13(8 to 19) | 1.08(0.65 to 1.60) | 9(6 to 14) | 0.42(0.25 to 0.63) | -3.08(-4.47 to -1.68) | 1083(654 to 1598) | 88.57(53.53 to 130.70) | 1025(616 to 1550) | 46.62(27.99 to 70.42) | -2.02(-2.92 to -1.12) |
| **Italy** | 64(39 to 91) | 0.45(0.27 to 0.64) | 23(14 to 33) | 0.19(0.12 to 0.27) | -2.80(-3.25 to -2.34) | 8309(4909 to 12844) | 58.80(34.71 to 90.86) | 4646(2666 to 7428) | 42.26(24.04 to 67.86) | -1.16(-1.35 to -0.96) |
| **Jamaica** | 25(15 to 39) | 4.35(2.56 to 6.65) | 80(43 to 131) | 10.23(5.48 to 16.73) | 2.93(1.40 to 4.48) | 1745(1013 to 2622) | 286.51(168.82 to 432.64) | 4812(2606 to 7768) | 615.30(333.55 to 993.08) | 2.92(2.24 to 3.60) |
| **Japan** | 54(32 to 85) | 0.17(0.10 to 0.26) | 26(15 to 40) | 0.10(0.06 to 0.16) | -1.28(-1.91 to -0.65) | 7799(4426 to 12692) | 24.84(13.95 to 40.73) | 4541(2565 to 7435) | 19.44(10.84 to 32.43) | -0.75(-1.18 to -0.31) |
| **Jordan** | 13(7 to 23) | 1.61(0.80 to 2.82) | 28(13 to 51) | 0.90(0.41 to 1.65) | -1.89(-2.65 to -1.11) | 1375(793 to 2132) | 163.41(95.87 to 252.46) | 3453(1946 to 5476) | 110.78(62.81 to 175.18) | -1.23(-1.59 to -0.88) |
| **Kazakhstan** | 83(49 to 132) | 2.03(1.21 to 3.22) | 36(21 to 56) | 0.72(0.42 to 1.13) | -3.31(-4.17 to -2.45) | 5847(3483 to 9255) | 141.23(84.20 to 222.77) | 2626(1552 to 4102) | 53.74(31.46 to 84.60) | -3.07(-3.75 to -2.39) |
| **Kenya** | 88(48 to 144) | 1.69(0.92 to 2.77) | 254(133 to 443) | 1.92(1.02 to 3.32) | 0.20(-0.37 to 0.77) | 8641(4866 to 13490) | 158.89(90.43 to 247.57) | 23209(12841 to 37495) | 170.17(95.39 to 272.59) | 0.07(-0.27 to 0.41) |
| **Kiribati** | 0(0 to 1) | 1.58(0.73 to 2.95) | 0(0 to 0) | 0.42(0.17 to 0.87) | -4.20(-4.39 to -4.02) | 42(23 to 68) | 216.93(121.95 to 352.09) | 40(21 to 68) | 121.82(65.56 to 207.86) | -1.86(-1.98 to -1.75) |
| **Kuwait** | 1(1 to 2) | 0.33(0.20 to 0.50) | 4(2 to 6) | 0.29(0.17 to 0.43) | -1.14(-5.84 to 3.79) | 307(185 to 465) | 72.68(43.95 to 110.39) | 953(578 to 1471) | 65.58(38.99 to 101.20) | -0.58(-2.13 to 0.99) |
| **Kyrgyzstan** | 20(12 to 33) | 2.11(1.23 to 3.39) | 10(5 to 16) | 0.55(0.30 to 0.92) | -4.12(-5.04 to -3.20) | 1420(825 to 2291) | 140.97(82.63 to 225.03) | 797(456 to 1299) | 45.86(26.25 to 74.79) | -3.46(-4.14 to -2.78) |
| **Lao People's Democratic Republic** | 30(10 to 63) | 3.11(1.09 to 6.44) | 34(14 to 68) | 1.67(0.69 to 3.39) | -2.03(-2.27 to -1.79) | 2305(982 to 4424) | 233.85(101.90 to 444.00) | 2567(1207 to 4789) | 127.25(60.07 to 237.09) | -1.97(-2.20 to -1.74) |
| **Latvia** | 9(5 to 14) | 1.34(0.78 to 2.16) | 2(1 to 3) | 0.40(0.22 to 0.66) | -3.87(-5.41 to -2.31) | 615(358 to 981) | 93.53(54.03 to 149.75) | 136(79 to 217) | 33.32(19.09 to 53.91) | -3.38(-4.18 to -2.57) |
| **Lebanon** | 108(69 to 145) | 14.37(9.29 to 19.35) | 11(5 to 21) | 0.70(0.31 to 1.36) | -8.62(-13.95 to -2.96) | 7736(4945 to 10389) | 1020.15(656.73 to 1369.23) | 1361(795 to 2176) | 89.85(52.15 to 143.54) | -5.41(-6.85 to -3.96) |
| **Lesotho** | 17(8 to 29) | 4.35(2.12 to 7.60) | 44(19 to 85) | 8.54(3.54 to 16.49) | 2.20(1.34 to 3.07) | 1262(664 to 2083) | 319.75(168.63 to 529.79) | 3072(1403 to 5635) | 578.52(264.00 to 1065.16) | 1.94(1.21 to 2.68) |
| **Liberia** | 7(3 to 11) | 1.10(0.57 to 1.85) | 14(5 to 28) | 0.92(0.35 to 1.90) | -3.31(-5.86 to -0.70) | 711(398 to 1123) | 118.64(67.09 to 186.68) | 1462(739 to 2554) | 99.81(50.91 to 173.62) | -2.73(-4.73 to -0.69) |
| **Libya** | 7(3 to 13) | 0.80(0.36 to 1.54) | 19(8 to 43) | 0.95(0.38 to 2.11) | 0.24(-5.25 to 6.04) | 890(502 to 1434) | 98.72(56.82 to 157.52) | 3171(1878 to 4882) | 158.86(93.74 to 244.40) | 1.41(-0.69 to 3.54) |
| **Lithuania** | 10(6 to 16) | 1.10(0.64 to 1.76) | 2(1 to 3) | 0.27(0.15 to 0.43) | -4.46(-5.90 to -2.99) | 711(413 to 1138) | 76.53(44.34 to 122.77) | 154(89 to 245) | 26.05(14.92 to 41.98) | -3.51(-4.51 to -2.50) |
| **Luxembourg** | 1(1 to 1) | 1.02(0.61 to 1.50) | 1(0 to 1) | 0.34(0.20 to 0.52) | -3.49(-4.80 to -2.16) | 82(50 to 120) | 83.94(51.06 to 123.93) | 63(38 to 95) | 41.85(24.91 to 63.64) | -2.31(-3.45 to -1.16) |
| **Madagascar** | 12(5 to 22) | 0.41(0.19 to 0.73) | 51(20 to 111) | 0.66(0.26 to 1.42) | 1.52(0.47 to 2.57) | 2058(1133 to 3420) | 71.73(39.94 to 118.72) | 6513(3336 to 11751) | 84.31(43.72 to 150.35) | 0.51(-0.06 to 1.08) |
| **Malawi** | 12(4 to 24) | 0.47(0.18 to 0.96) | 22(7 to 55) | 0.40(0.14 to 1.00) | -0.52(-1.16 to 0.13) | 2070(1079 to 3706) | 85.12(45.00 to 150.49) | 4646(2397 to 8511) | 86.74(45.48 to 156.10) | 0.04(-0.09 to 0.18) |
| **Malaysia** | 19(9 to 33) | 0.42(0.21 to 0.72) | 25(11 to 49) | 0.30(0.13 to 0.58) | -1.01(-1.71 to -0.31) | 2160(1197 to 3507) | 47.17(26.31 to 76.36) | 3092(1667 to 5203) | 36.43(19.63 to 61.36) | -0.80(-1.18 to -0.42) |
| **Maldives** | 0(0 to 0) | 0.40(0.18 to 0.77) | 0(0 to 0) | 0.12(0.05 to 0.24) | -3.93(-4.52 to -3.33) | 23(12 to 39) | 45.98(24.76 to 77.41) | 29(16 to 49) | 25.38(13.64 to 43.06) | -1.88(-2.18 to -1.58) |
| **Mali** | 43(18 to 85) | 2.19(0.90 to 4.33) | 154(70 to 288) | 2.70(1.24 to 5.03) | 0.75(0.37 to 1.14) | 3881(1941 to 6908) | 194.82(98.40 to 344.48) | 12816(6592 to 22348) | 219.42(114.29 to 378.96) | 0.42(0.13 to 0.71) |
| **Malta** | 1(0 to 1) | 0.71(0.43 to 1.05) | 0(0 to 1) | 0.34(0.21 to 0.51) | -1.90(-4.08 to 0.33) | 61(37 to 89) | 65.99(39.55 to 96.94) | 38(23 to 57) | 40.65(24.31 to 61.47) | -1.32(-2.68 to 0.05) |
| **Marshall Islands** | 0(0 to 0) | 2.35(1.07 to 4.42) | 0(0 to 1) | 2.03(0.79 to 4.01) | -0.55(-0.77 to -0.32) | 22(12 to 37) | 223.52(120.50 to 372.56) | 30(15 to 51) | 199.77(100.53 to 343.57) | -0.41(-0.55 to -0.27) |
| **Mauritania** | 6(2 to 12) | 1.27(0.50 to 2.55) | 13(5 to 28) | 1.22(0.50 to 2.49) | -0.11(-0.38 to 0.16) | 564(275 to 997) | 113.39(56.14 to 200.16) | 1176(569 to 2154) | 104.55(51.21 to 190.37) | -0.26(-0.46 to -0.07) |
| **Mauritius** | 2(1 to 2) | 0.51(0.29 to 0.81) | 1(1 to 2) | 0.34(0.19 to 0.56) | -1.38(-3.54 to 0.83) | 162(93 to 255) | 53.31(30.76 to 83.67) | 129(74 to 205) | 41.17(23.42 to 65.68) | -0.93(-2.27 to 0.43) |
| **Mexico** | 186(108 to 299) | 0.82(0.48 to 1.31) | 539(310 to 862) | 1.55(0.89 to 2.48) | 2.09(1.21 to 2.97) | 13704(7908 to 22480) | 58.99(34.32 to 95.96) | 35637(20517 to 57450) | 102.95(59.27 to 166.01) | 1.85(1.13 to 2.57) |
| **Micronesia (Federated States of)** | 1(0 to 1) | 2.93(1.26 to 5.70) | 1(0 to 1) | 2.85(1.19 to 5.75) | -0.08(-0.16 to 0.00) | 62(32 to 107) | 263.59(137.88 to 455.58) | 66(34 to 119) | 253.18(129.14 to 452.49) | -0.13(-0.18 to -0.08) |
| **Monaco** | 0(0 to 0) | 0.04(0.02 to 0.08) | 0(0 to 0) | 0.04(0.01 to 0.07) | -0.52(-0.60 to -0.45) | 1(1 to 2) | 23.07(12.65 to 37.70) | 1(1 to 2) | 22.47(12.32 to 36.78) | -0.09(-0.12 to -0.06) |
| **Mongolia** | 9(4 to 17) | 1.90(0.89 to 3.51) | 6(3 to 11) | 0.70(0.34 to 1.29) | -3.07(-4.00 to -2.13) | 665(337 to 1186) | 134.49(69.14 to 236.76) | 481(260 to 825) | 55.73(29.80 to 96.60) | -2.78(-3.42 to -2.14) |
| **Montenegro** | 2(1 to 3) | 1.08(0.55 to 1.91) | 1(0 to 2) | 0.58(0.29 to 1.04) | -1.68(-2.73 to -0.62) | 130(71 to 221) | 83.17(45.51 to 141.19) | 74(42 to 125) | 51.87(29.04 to 87.63) | -1.33(-2.17 to -0.49) |
| **Morocco** | 14(7 to 26) | 0.22(0.11 to 0.40) | 43(19 to 87) | 0.44(0.19 to 0.90) | 2.25(1.92 to 2.57) | 4200(2487 to 6503) | 66.15(39.50 to 102.19) | 7152(4225 to 11511) | 73.79(43.52 to 118.82) | 0.37(0.19 to 0.55) |
| **Mozambique** | 39(17 to 78) | 1.19(0.52 to 2.35) | 136(52 to 300) | 1.67(0.65 to 3.64) | 1.06(0.70 to 1.42) | 3949(2023 to 6969) | 118.95(61.83 to 207.69) | 12166(5622 to 23637) | 147.17(69.69 to 281.23) | 0.67(0.45 to 0.90) |
| **Myanmar** | 120(55 to 224) | 1.09(0.51 to 2.03) | 56(23 to 114) | 0.37(0.15 to 0.75) | -3.32(-3.76 to -2.88) | 9962(4973 to 17440) | 90.50(46.09 to 156.76) | 5780(2979 to 10212) | 38.16(19.71 to 67.30) | -2.70(-3.00 to -2.40) |
| **Namibia** | 16(7 to 30) | 4.58(2.08 to 8.58) | 25(10 to 52) | 3.70(1.48 to 7.75) | -0.75(-1.14 to -0.35) | 1183(590 to 2122) | 325.46(164.56 to 580.49) | 1780(816 to 3509) | 261.77(120.44 to 514.50) | -0.80(-1.30 to -0.31) |
| **Nauru** | 0(0 to 0) | 3.04(1.01 to 6.29) | 0(0 to 0) | 3.42(1.24 to 7.08) | 0.37(0.24 to 0.50) | 7(3 to 12) | 265.08(124.94 to 478.77) | 8(4 to 15) | 285.94(134.50 to 528.07) | 0.23(0.14 to 0.33) |
| **Nepal** | 18(8 to 36) | 0.38(0.17 to 0.75) | 49(19 to 102) | 0.53(0.21 to 1.10) | 1.11(0.86 to 1.36) | 3141(1662 to 5500) | 65.98(35.20 to 114.40) | 6619(3394 to 11872) | 71.08(36.63 to 126.73) | 0.28(0.05 to 0.52) |
| **Netherlands** | 21(13 to 32) | 0.54(0.33 to 0.81) | 10(6 to 16) | 0.28(0.17 to 0.42) | -2.21(-3.63 to -0.77) | 2192(1340 to 3331) | 55.92(34.08 to 85.17) | 1406(838 to 2159) | 39.35(23.26 to 60.63) | -1.19(-1.90 to -0.48) |
| **New Zealand** | 9(5 to 13) | 0.97(0.59 to 1.43) | 6(4 to 9) | 0.52(0.31 to 0.76) | -2.50(-2.97 to -2.02) | 764(468 to 1123) | 84.61(51.68 to 124.48) | 629(380 to 922) | 53.56(32.13 to 78.64) | -1.86(-2.18 to -1.54) |
| **Nicaragua** | 11(6 to 19) | 1.18(0.61 to 2.02) | 12(6 to 23) | 0.65(0.30 to 1.25) | -2.01(-2.90 to -1.10) | 844(447 to 1438) | 86.53(46.24 to 146.17) | 950(482 to 1739) | 51.68(26.26 to 94.51) | -1.73(-2.59 to -0.87) |
| **Niger** | 28(11 to 56) | 1.55(0.64 to 3.12) | 100(40 to 213) | 1.76(0.72 to 3.71) | 0.29(-0.15 to 0.73) | 2633(1338 to 4693) | 145.36(75.05 to 255.61) | 8701(4240 to 16515) | 151.35(75.67 to 280.55) | 0.06(-0.28 to 0.40) |
| **Nigeria** | 402(207 to 679) | 2.01(1.04 to 3.39) | 1022(451 to 2030) | 1.76(0.79 to 3.46) | -0.50(-2.47 to 1.51) | 34163(18761 to 53573) | 164.73(91.86 to 257.23) | 85624(43608 to 154354) | 144.00(74.54 to 255.78) | -0.60(-1.42 to 0.22) |
| **Niue** | 0(0 to 0) | 1.68(0.70 to 3.46) | 0(0 to 0) | 2.64(1.11 to 5.20) | 1.48(0.81 to 2.16) | 1(0 to 2) | 178.34(93.81 to 312.94) | 1(0 to 2) | 238.38(119.18 to 420.88) | 0.97(0.55 to 1.40) |
| **North Macedonia** | 4(2 to 6) | 0.71(0.38 to 1.24) | 1(1 to 3) | 0.27(0.13 to 0.49) | -3.06(-4.29 to -1.81) | 331(185 to 548) | 65.02(36.34 to 107.57) | 180(101 to 301) | 34.20(19.09 to 57.76) | -2.08(-2.72 to -1.43) |
| **Northern Mariana Islands** | 0(0 to 1) | 3.16(1.35 to 6.15) | 0(0 to 0) | 1.78(0.80 to 3.27) | -1.86(-2.11 to -1.62) | 37(19 to 66) | 258.53(130.18 to 460.57) | 19(10 to 32) | 175.17(93.71 to 293.44) | -1.24(-1.41 to -1.08) |
| **Norway** | 6(4 to 9) | 0.56(0.35 to 0.81) | 5(3 to 7) | 0.39(0.24 to 0.56) | -1.78(-3.18 to -0.37) | 573(354 to 832) | 54.88(33.81 to 79.62) | 522(321 to 769) | 44.86(27.30 to 66.15) | -1.03(-1.87 to -0.19) |
| **Oman** | 1(0 to 1) | 0.18(0.08 to 0.35) | 1(0 to 2) | 0.09(0.04 to 0.17) | -2.14(-2.85 to -1.44) | 211(121 to 332) | 61.94(35.79 to 97.43) | 541(312 to 874) | 52.61(30.03 to 84.98) | -0.53(-0.64 to -0.41) |
| **Pakistan** | 220(111 to 387) | 0.89(0.45 to 1.55) | 586(254 to 1122) | 0.94(0.41 to 1.79) | 0.32(-0.01 to 0.65) | 23626(12637 to 40322) | 94.70(51.32 to 159.61) | 59328(30066 to 103415) | 94.24(48.06 to 163.63) | 0.07(-0.13 to 0.26) |
| **Palau** | 0(0 to 0) | 4.31(1.80 to 8.70) | 0(0 to 0) | 4.85(2.04 to 9.18) | 0.37(0.25 to 0.49) | 14(7 to 27) | 342.27(166.21 to 628.81) | 13(6 to 23) | 385.31(181.17 to 681.53) | 0.38(0.27 to 0.48) |
| **Palestine** | 3(1 to 5) | 0.61(0.27 to 1.20) | 21(10 to 36) | 1.58(0.79 to 2.76) | 3.15(2.65 to 3.66) | 388(221 to 609) | 87.43(50.53 to 136.52) | 1875(1059 to 2963) | 141.42(80.61 to 223.23) | 1.61(1.30 to 1.93) |
| **Panama** | 5(3 to 9) | 0.86(0.47 to 1.43) | 12(6 to 21) | 1.14(0.60 to 1.98) | 1.16(-0.28 to 2.61) | 418(231 to 704) | 64.41(35.87 to 107.76) | 874(474 to 1486) | 81.83(44.46 to 139.01) | 0.96(-0.25 to 2.18) |
| **Papua New Guinea** | 20(7 to 43) | 2.09(0.75 to 4.37) | 103(43 to 204) | 3.87(1.60 to 7.64) | 1.93(1.10 to 2.77) | 2038(1009 to 3691) | 204.77(102.82 to 368.68) | 8796(4340 to 15566) | 329.01(162.80 to 580.79) | 1.52(1.04 to 2.00) |
| **Paraguay** | 9(4 to 16) | 0.91(0.45 to 1.65) | 21(9 to 40) | 1.09(0.49 to 2.09) | 0.65(-0.10 to 1.40) | 684(350 to 1216) | 68.95(35.53 to 121.72) | 1531(739 to 2853) | 79.18(38.31 to 147.34) | 0.52(-0.11 to 1.14) |
| **Peru** | 94(50 to 154) | 1.71(0.91 to 2.78) | 102(50 to 181) | 1.06(0.52 to 1.88) | -1.64(-2.77 to -0.49) | 7491(4287 to 11574) | 133.14(76.76 to 205.61) | 8796(5056 to 14136) | 91.54(52.47 to 147.35) | -1.27(-2.14 to -0.38) |
| **Philippines** | 592(350 to 897) | 3.79(2.27 to 5.71) | 465(267 to 736) | 1.60(0.92 to 2.54) | -2.72(-3.33 to -2.11) | 42720(25382 to 64714) | 267.63(161.34 to 402.99) | 35288(20737 to 55030) | 120.08(70.91 to 187.14) | -2.55(-3.05 to -2.06) |
| **Poland** | 38(23 to 60) | 0.39(0.23 to 0.62) | 10(6 to 16) | 0.11(0.06 to 0.17) | -3.99(-4.70 to -3.28) | 3126(1844 to 4998) | 32.78(19.12 to 52.88) | 1233(707 to 2000) | 14.26(8.03 to 23.42) | -2.62(-3.12 to -2.12) |
| **Portugal** | 32(20 to 45) | 1.28(0.80 to 1.77) | 11(7 to 15) | 0.45(0.28 to 0.62) | -3.42(-4.89 to -1.92) | 2632(1664 to 3634) | 104.51(66.11 to 144.25) | 1156(742 to 1606) | 50.93(32.54 to 70.80) | -2.26(-2.96 to -1.55) |
| **Puerto Rico** | 39(23 to 58) | 4.08(2.39 to 6.12) | 15(9 to 24) | 2.09(1.16 to 3.28) | -2.18(-3.67 to -0.66) | 2592(1527 to 3853) | 271.39(159.95 to 403.28) | 1041(596 to 1611) | 144.00(82.26 to 222.51) | -2.08(-3.43 to -0.72) |
| **Qatar** | 0(0 to 0) | 0.18(0.09 to 0.31) | 1(1 to 2) | 0.21(0.10 to 0.39) | 0.71(-0.73 to 2.18) | 51(30 to 79) | 63.24(37.10 to 98.48) | 338(197 to 534) | 60.20(34.29 to 95.64) | -0.14(-0.42 to 0.15) |
| **Republic of Korea** | 63(34 to 105) | 0.50(0.27 to 0.83) | 27(13 to 47) | 0.22(0.10 to 0.39) | -2.55(-3.12 to -1.98) | 5411(3020 to 8899) | 42.23(23.67 to 69.14) | 2632(1423 to 4461) | 23.09(12.28 to 39.84) | -1.86(-2.22 to -1.49) |
| **Republic of Moldova** | 16(9 to 26) | 1.42(0.82 to 2.26) | 4(2 to 7) | 0.44(0.25 to 0.71) | -3.87(-5.56 to -2.16) | 1136(663 to 1803) | 100.20(58.05 to 160.00) | 342(199 to 546) | 38.54(22.01 to 62.68) | -3.13(-4.03 to -2.21) |
| **Romania** | 65(40 to 99) | 1.15(0.71 to 1.76) | 14(8 to 22) | 0.33(0.19 to 0.53) | -3.97(-5.53 to -2.37) | 5330(3323 to 8158) | 95.25(59.25 to 145.97) | 1804(1050 to 2870) | 45.55(26.30 to 73.31) | -2.36(-3.42 to -1.30) |
| **Russian Federation** | 1023(619 to 1586) | 2.70(1.62 to 4.21) | 537(321 to 823) | 1.45(0.85 to 2.25) | -2.15(-3.67 to -0.60) | 70472(42134 to 110245) | 186.92(110.63 to 293.96) | 35340(21488 to 54068) | 99.25(59.35 to 154.28) | -1.90(-3.23 to -0.54) |
| **Rwanda** | 51(26 to 87) | 2.89(1.49 to 5.00) | 37(14 to 78) | 0.99(0.39 to 2.09) | -3.43(-3.67 to -3.19) | 4313(2359 to 7103) | 244.17(134.93 to 400.26) | 4180(2082 to 7435) | 112.83(56.79 to 199.54) | -2.48(-2.95 to -2.01) |
| **Saint Kitts and Nevis** | 0(0 to 0) | 2.28(1.28 to 3.58) | 0(0 to 0) | 0.89(0.46 to 1.49) | -3.21(-4.78 to -1.62) | 18(10 to 27) | 164.80(94.36 to 255.11) | 12(7 to 19) | 79.95(43.76 to 127.63) | -2.51(-3.82 to -1.19) |
| **Saint Lucia** | 1(1 to 2) | 3.43(1.94 to 5.38) | 1(1 to 2) | 2.51(1.34 to 3.99) | -1.15(-2.34 to 0.06) | 85(48 to 132) | 235.88(135.49 to 365.45) | 79(44 to 123) | 178.22(98.50 to 277.05) | -1.03(-2.10 to 0.06) |
| **Saint Vincent and the Grenadines** | 2(1 to 3) | 6.01(3.40 to 9.43) | 2(1 to 2) | 5.49(2.98 to 8.73) | -0.64(-3.06 to 1.85) | 116(65 to 179) | 402.12(228.20 to 623.39) | 100(55 to 156) | 366.37(201.92 to 571.40) | -0.62(-2.67 to 1.48) |
| **Samoa** | 1(0 to 2) | 2.00(0.81 to 4.33) | 1(0 to 2) | 2.22(0.94 to 4.49) | 0.35(0.25 to 0.44) | 71(36 to 131) | 191.22(98.28 to 349.98) | 98(50 to 175) | 200.14(104.14 to 356.36) | 0.15(0.09 to 0.20) |
| **San Marino** | 0(0 to 0) | 0.48(0.21 to 0.96) | 0(0 to 0) | 0.23(0.09 to 0.47) | -2.60(-3.01 to -2.18) | 3(2 to 5) | 48.48(25.37 to 83.92) | 2(1 to 4) | 33.22(17.97 to 55.76) | -1.25(-1.67 to -0.83) |
| **Sao Tome and Principe** | 0(0 to 1) | 1.47(0.56 to 3.07) | 1(0 to 2) | 1.50(0.56 to 3.46) | -0.04(-0.71 to 0.64) | 36(17 to 67) | 132.19(65.33 to 245.21) | 76(36 to 149) | 131.52(62.91 to 256.78) | -0.05(-0.57 to 0.47) |
| **Saudi Arabia** | 116(52 to 225) | 3.67(1.64 to 7.20) | 254(108 to 482) | 2.40(1.02 to 4.54) | -1.38(-1.62 to -1.14) | 8710(4355 to 15641) | 265.07(133.22 to 478.00) | 19017(9668 to 32455) | 182.82(92.47 to 311.64) | -1.18(-1.39 to -0.97) |
| **Senegal** | 7(3 to 14) | 0.38(0.16 to 0.75) | 11(4 to 25) | 0.28(0.11 to 0.60) | -0.58(-1.05 to -0.11) | 976(528 to 1622) | 53.50(29.35 to 88.52) | 1815(970 to 3069) | 44.54(24.04 to 74.86) | -0.39(-0.61 to -0.17) |
| **Serbia** | 25(13 to 43) | 1.04(0.55 to 1.82) | 8(4 to 13) | 0.36(0.19 to 0.62) | -4.29(-4.59 to -3.98) | 1995(1114 to 3289) | 85.56(47.52 to 141.63) | 766(439 to 1257) | 37.54(21.27 to 62.09) | -3.27(-3.48 to -3.05) |
| **Seychelles** | 0(0 to 1) | 1.57(0.77 to 2.82) | 0(0 to 0) | 0.89(0.39 to 1.68) | -1.51(-2.12 to -0.90) | 23(12 to 39) | 121.48(64.57 to 205.99) | 18(9 to 31) | 73.36(36.38 to 129.57) | -1.35(-1.84 to -0.85) |
| **Sierra Leone** | 3(1 to 7) | 0.31(0.12 to 0.69) | 11(4 to 24) | 0.43(0.16 to 0.99) | 0.78(0.24 to 1.33) | 591(322 to 994) | 55.84(30.76 to 93.47) | 1414(726 to 2509) | 58.17(30.18 to 102.13) | -0.04(-0.27 to 0.18) |
| **Singapore** | 3(2 to 6) | 0.37(0.21 to 0.60) | 2(1 to 2) | 0.11(0.06 to 0.18) | -3.75(-4.76 to -2.73) | 341(195 to 555) | 36.07(20.66 to 58.75) | 236(134 to 387) | 18.18(10.03 to 30.81) | -2.18(-2.66 to -1.69) |
| **Slovakia** | 13(7 to 21) | 0.94(0.50 to 1.59) | 5(2 to 9) | 0.38(0.19 to 0.68) | -2.82(-3.44 to -2.19) | 1014(578 to 1654) | 75.98(43.08 to 124.44) | 494(273 to 817) | 39.57(21.52 to 65.87) | -2.11(-2.45 to -1.78) |
| **Slovenia** | 4(2 to 6) | 0.81(0.49 to 1.21) | 1(0 to 1) | 0.13(0.08 to 0.21) | -6.06(-7.08 to -5.03) | 459(280 to 691) | 91.94(55.78 to 138.56) | 161(95 to 249) | 38.46(22.34 to 59.80) | -2.81(-3.28 to -2.33) |
| **Solomon Islands** | 1(0 to 2) | 1.32(0.42 to 2.80) | 3(1 to 5) | 1.68(0.77 to 3.21) | 0.67(0.33 to 1.01) | 122(62 to 210) | 163.98(84.58 to 282.22) | 305(165 to 514) | 177.17(96.08 to 297.74) | 0.20(-0.07 to 0.46) |
| **Somalia** | 39(16 to 79) | 2.27(0.91 to 4.59) | 188(68 to 435) | 3.77(1.38 to 8.62) | -0.29(-1.49 to 0.93) | 3361(1631 to 6137) | 193.32(94.40 to 351.29) | 14549(6183 to 30556) | 284.07(123.23 to 588.90) | -0.29(-1.31 to 0.73) |
| **South Africa** | 1960(1202 to 2844) | 20.06(12.36 to 29.29) | 1068(584 to 1673) | 6.82(3.72 to 10.69) | -3.41(-4.36 to -2.46) | 127574(79218 to 183178) | 1282.23(800.75 to 1850.71) | 68924(38816 to 106080) | 441.80(248.22 to 680.10) | -3.32(-4.18 to -2.45) |
| **South Sudan** | 20(8 to 44) | 1.51(0.57 to 3.25) | 112(57 to 206) | 4.78(2.47 to 8.67) | 3.58(2.28 to 4.90) | 1955(930 to 3727) | 140.98(68.01 to 264.63) | 8189(4314 to 14521) | 341.06(182.51 to 595.70) | 2.72(1.71 to 3.74) |
| **Spain** | 43(29 to 58) | 0.45(0.30 to 0.60) | 26(17 to 37) | 0.25(0.16 to 0.36) | -1.91(-3.39 to -0.41) | 5166(3417 to 7265) | 53.42(35.41 to 74.98) | 3775(2338 to 5551) | 40.88(24.95 to 60.70) | -0.95(-1.49 to -0.40) |
| **Sri Lanka** | 71(35 to 120) | 1.50(0.75 to 2.55) | 26(10 to 53) | 0.47(0.19 to 0.95) | -3.64(-4.34 to -2.92) | 5816(3125 to 9568) | 122.93(66.49 to 201.55) | 2636(1346 to 4743) | 47.35(24.11 to 85.44) | -2.98(-3.40 to -2.55) |
| **Sudan** | 9(5 to 14) | 0.18(0.10 to 0.30) | 113(64 to 189) | 0.99(0.56 to 1.66) | 3.51(-8.74 to 17.40) | 3066(1792 to 4764) | 65.11(38.51 to 101.12) | 12601(7638 to 19345) | 109.71(66.75 to 167.99) | 0.13(-1.33 to 1.62) |
| **Suriname** | 3(2 to 6) | 3.38(1.54 to 5.87) | 5(2 to 9) | 3.16(1.37 to 5.97) | -0.01(-0.58 to 0.56) | 248(119 to 419) | 238.91(116.56 to 402.54) | 318(152 to 575) | 221.94(106.24 to 401.48) | -0.06(-0.55 to 0.44) |
| **Sweden** | 16(9 to 23) | 0.75(0.45 to 1.09) | 8(5 to 12) | 0.37(0.22 to 0.55) | -2.41(-3.02 to -1.79) | 1386(845 to 2070) | 69.01(41.91 to 102.96) | 982(584 to 1483) | 46.61(27.35 to 70.75) | -1.41(-2.17 to -0.64) |
| **Switzerland** | 21(17 to 26) | 1.18(0.94 to 1.47) | 6(4 to 8) | 0.30(0.19 to 0.43) | -4.58(-5.77 to -3.37) | 1725(1391 to 2132) | 99.11(79.67 to 122.95) | 869(544 to 1245) | 46.46(28.71 to 66.95) | -2.49(-3.12 to -1.86) |
| **Syrian Arab Republic** | 20(11 to 33) | 0.74(0.39 to 1.23) | 51(30 to 81) | 1.42(0.84 to 2.20) | 3.63(1.05 to 6.28) | 2713(1609 to 4085) | 97.43(58.54 to 146.38) | 4896(2949 to 7381) | 132.98(81.51 to 197.57) | 1.44(-0.13 to 3.02) |
| **Taiwan (Province of China)** | 60(36 to 87) | 1.07(0.65 to 1.56) | 20(12 to 32) | 0.35(0.20 to 0.55) | -3.60(-4.75 to -2.44) | 8489(5098 to 13002) | 151.35(90.67 to 232.29) | 5663(3171 to 9352) | 103.38(57.56 to 170.22) | -1.25(-1.58 to -0.93) |
| **Tajikistan** | 11(6 to 19) | 1.00(0.53 to 1.69) | 6(3 to 12) | 0.24(0.10 to 0.47) | -4.02(-6.12 to -1.88) | 887(485 to 1479) | 76.05(42.16 to 124.96) | 703(370 to 1221) | 27.20(14.35 to 47.05) | -2.90(-4.32 to -1.46) |
| **Thailand** | 237(91 to 513) | 1.47(0.58 to 3.16) | 147(69 to 269) | 0.90(0.42 to 1.66) | -1.54(-2.44 to -0.63) | 18746(8612 to 37030) | 114.92(53.36 to 224.75) | 11397(5955 to 19562) | 72.15(37.28 to 124.64) | -1.45(-2.12 to -0.79) |
| **Timor-Leste** | 2(1 to 4) | 0.88(0.35 to 1.88) | 3(1 to 7) | 0.85(0.33 to 1.84) | -0.20(-1.16 to 0.78) | 155(75 to 293) | 79.60(39.10 to 149.23) | 274(126 to 553) | 73.72(34.68 to 144.99) | -0.29(-0.93 to 0.36) |
| **Togo** | 15(6 to 28) | 1.70(0.72 to 3.21) | 41(17 to 86) | 1.89(0.77 to 3.93) | 0.26(-0.04 to 0.57) | 1247(614 to 2159) | 138.47(69.45 to 238.60) | 3278(1572 to 6180) | 148.19(71.48 to 278.53) | 0.14(-0.11 to 0.39) |
| **Tokelau** | 0(0 to 0) | 1.85(0.68 to 4.23) | 0(0 to 0) | 2.49(0.96 to 5.57) | 1.22(0.68 to 1.76) | 1(0 to 1) | 195.27(99.14 to 364.23) | 1(0 to 1) | 230.95(110.05 to 445.48) | 0.70(0.35 to 1.05) |
| **Tonga** | 1(0 to 1) | 2.66(1.29 to 4.67) | 0(0 to 0) | 0.46(0.19 to 0.92) | -5.48(-6.02 to -4.94) | 49(26 to 80) | 223.57(121.94 to 365.90) | 22(12 to 37) | 85.23(46.12 to 144.18) | -3.14(-3.25 to -3.03) |
| **Trinidad and Tobago** | 12(7 to 19) | 3.82(2.20 to 5.96) | 17(9 to 28) | 5.03(2.62 to 8.23) | 0.69(-1.58 to 3.01) | 823(480 to 1260) | 258.61(151.06 to 397.17) | 1078(584 to 1730) | 329.75(177.36 to 528.90) | 0.59(-0.85 to 2.06) |
| **Tunisia** | 6(3 to 12) | 0.32(0.15 to 0.60) | 7(3 to 13) | 0.21(0.09 to 0.40) | -2.14(-5.79 to 1.65) | 1425(840 to 2244) | 69.28(41.24 to 108.85) | 1819(1045 to 2907) | 59.42(33.88 to 95.09) | -0.81(-2.27 to 0.68) |
| **Turkey** | 138(64 to 253) | 0.98(0.46 to 1.80) | 136(73 to 223) | 0.63(0.34 to 1.03) | -1.35(-2.01 to -0.69) | 15978(9498 to 25065) | 111.91(67.29 to 174.81) | 19557(12249 to 29258) | 91.12(56.96 to 136.18) | -0.59(-0.74 to -0.44) |
| **Turkmenistan** | 10(5 to 16) | 1.13(0.64 to 1.79) | 7(4 to 13) | 0.59(0.30 to 1.02) | -2.07(-3.54 to -0.57) | 732(420 to 1171) | 82.84(48.15 to 131.15) | 615(330 to 1036) | 48.74(26.18 to 82.05) | -1.68(-2.85 to -0.51) |
| **Tuvalu** | 0(0 to 0) | 3.05(1.28 to 6.03) | 0(0 to 0) | 2.59(1.06 to 5.21) | -0.51(-0.62 to -0.41) | 7(3 to 12) | 273.23(140.08 to 469.27) | 7(3 to 12) | 234.58(119.79 to 414.49) | -0.48(-0.55 to -0.42) |
| **Uganda** | 40(15 to 86) | 0.96(0.36 to 2.05) | 145(53 to 325) | 1.27(0.47 to 2.85) | 1.05(0.30 to 1.81) | 5493(2807 to 9747) | 131.03(68.42 to 229.92) | 17211(8529 to 31254) | 151.49(76.19 to 272.47) | 0.38(0.10 to 0.67) |
| **Ukraine** | 174(103 to 276) | 1.35(0.79 to 2.15) | 89(40 to 165) | 0.82(0.37 to 1.52) | -1.57(-2.39 to -0.75) | 12233(7227 to 19342) | 95.76(56.18 to 152.48) | 6376(3191 to 11142) | 61.28(30.23 to 107.66) | -1.38(-2.09 to -0.66) |
| **United Arab Emirates** | 1(0 to 2) | 0.30(0.13 to 0.57) | 3(1 to 5) | 0.19(0.08 to 0.37) | -1.44(-2.33 to -0.54) | 246(143 to 390) | 70.50(40.96 to 111.84) | 968(565 to 1556) | 59.31(34.01 to 95.18) | -0.54(-0.79 to -0.30) |
| **United Kingdom** | 67(42 to 95) | 0.47(0.30 to 0.67) | 24(15 to 34) | 0.15(0.10 to 0.21) | -3.48(-5.08 to -1.86) | 7387(4490 to 10987) | 52.81(32.01 to 78.61) | 4762(2809 to 7425) | 32.35(18.88 to 50.77) | -1.52(-2.16 to -0.87) |
| **United Republic of Tanzania** | 120(52 to 238) | 1.86(0.82 to 3.67) | 254(96 to 531) | 1.60(0.61 to 3.31) | -0.48(-0.71 to -0.25) | 11181(5643 to 20269) | 171.17(88.25 to 306.37) | 24232(11517 to 44134) | 152.29(74.13 to 274.70) | -0.38(-0.60 to -0.15) |
| **United States of America** | 1695(1048 to 2567) | 2.51(1.54 to 3.82) | 1125(687 to 1707) | 1.48(0.90 to 2.26) | -1.72(-2.15 to -1.28) | 113381(69622 to 173983) | 169.98(103.42 to 262.57) | 77725(47023 to 119596) | 104.24(62.69 to 161.28) | -1.59(-1.97 to -1.21) |
| **United States Virgin Islands** | 2(1 to 3) | 6.98(3.43 to 12.44) | 1(0 to 1) | 4.33(1.91 to 8.58) | -0.66(-0.93 to -0.40) | 124(62 to 216) | 445.19(224.57 to 776.58) | 45(21 to 86) | 278.68(130.83 to 536.33) | -0.65(-0.91 to -0.39) |
| **Uruguay** | 9(5 to 14) | 1.17(0.66 to 1.91) | 9(5 to 15) | 1.08(0.59 to 1.76) | -0.25(-1.47 to 1.00) | 662(379 to 1080) | 88.22(50.48 to 143.94) | 664(378 to 1071) | 80.90(45.86 to 131.09) | -0.27(-1.26 to 0.73) |
| **Uzbekistan** | 50(30 to 80) | 1.11(0.66 to 1.74) | 31(17 to 52) | 0.34(0.19 to 0.57) | -3.78(-4.84 to -2.70) | 3759(2204 to 6048) | 79.44(47.35 to 126.11) | 3010(1728 to 4879) | 33.39(19.05 to 54.31) | -2.84(-3.52 to -2.15) |
| **Vanuatu** | 0(0 to 0) | 0.22(0.09 to 0.44) | 0(0 to 0) | 0.23(0.10 to 0.47) | 0.10(-0.23 to 0.44) | 32(17 to 54) | 87.61(47.91 to 148.34) | 67(36 to 117) | 84.09(45.52 to 145.98) | -0.15(-0.22 to -0.08) |
| **Venezuela (Bolivarian Republic of)** | 31(17 to 52) | 0.61(0.35 to 1.02) | 97(50 to 172) | 1.48(0.76 to 2.64) | 3.15(1.22 to 5.12) | 2309(1306 to 3881) | 44.96(25.55 to 75.15) | 6503(3436 to 11333) | 100.57(52.79 to 176.20) | 2.86(1.10 to 4.64) |
| **Viet Nam** | 63(26 to 129) | 0.36(0.15 to 0.73) | 53(22 to 106) | 0.21(0.09 to 0.42) | -1.76(-1.86 to -1.65) | 7852(4162 to 13905) | 44.25(23.79 to 77.25) | 8200(4403 to 13658) | 32.71(17.35 to 54.98) | -0.98(-1.03 to -0.93) |
| **Yemen** | 19(7 to 39) | 0.76(0.29 to 1.55) | 71(28 to 160) | 0.88(0.35 to 1.97) | 0.96(-1.93 to 3.94) | 2610(1453 to 4184) | 98.33(55.18 to 156.57) | 10088(5755 to 16313) | 121.54(69.66 to 196.21) | 0.87(-0.45 to 2.21) |
| **Zambia** | 42(20 to 74) | 2.10(1.03 to 3.69) | 128(49 to 273) | 2.51(0.99 to 5.32) | 0.57(0.38 to 0.76) | 3840(2033 to 6384) | 190.56(102.77 to 312.74) | 10789(5007 to 20880) | 206.57(97.81 to 394.87) | 0.27(0.13 to 0.41) |
| **Zimbabwe** | 51(25 to 89) | 2.05(1.02 to 3.54) | 131(57 to 255) | 3.13(1.35 to 6.10) | 1.28(0.34 to 2.24) | 4655(2603 to 7356) | 180.95(102.01 to 284.64) | 10530(5245 to 18796) | 247.10(123.59 to 441.11) | 0.99(0.68 to 1.31) |

**Notes:** Rates are reported per 100,000 person-years. Data in parentheses are 95% uncertainty intervals for cases and age-standardized rates of mortality and DALYs, and 95% confidence intervals for AAPCs. **Abbreviations:** DALYs, disability-adjusted life-years; ASMR, age-standardized mortality rate; ASDR, age-standardized DALYs rate; AAPC, average annual percent change; SDI, socio-demographic index; UI, uncertainty interval; CI, confidence interval.

**Table S4. Age-standardised mortality rates and DALY rates in 1990 and 2021, and AAPC from 1990 to 2021 for the disease burden attributable to IPV among women of childbearing age , by country.**

| **Location** | **Mortality** | | | | | **DALYs** | | | | |
| --- | --- | --- | --- | --- | --- | --- | --- | --- | --- | --- |
|  | **Mortality cases in 1990 (95% UI)** | **ASMR in 1990 (95% UI)** | **Mortality cases in 2021 (95% UI)** | **ASMR in 2021 (95% UI)** | **AAPC% (95%CI),**  **1990–2021** | **DALYs cases in 1990 (95% UI)** | **ASDR in 1990 (95% UI)** | **DALYs cases in 2021 (95% UI)** | **ASDR in 2021 (95% UI)** | **AAPC% (95%CI),**  **1990–2021** |
| **Afghanistan** | 82(37 to 152) | 4.08(1.83 to 7.59) | 261(117 to 499) | 3.82(1.72 to 7.31) | -0.66(-2.34 to 1.05) | 10202(4690 to 18016) | 510.55(231.70 to 897.51) | 36864(16198 to 64601) | 546.65(232.84 to 962.33) | 0.01(-0.23 to 0.25) |
| **Albania** | 9(5 to 16) | 1.06(0.54 to 1.85) | 3(1 to 6) | 0.47(0.21 to 0.90) | -2.79(-3.74 to -1.83) | 1231(540 to 2252) | 148.92(64.07 to 273.44) | 848(212 to 1898) | 134.78(34.20 to 301.44) | -0.33(-0.58 to -0.08) |
| **Algeria** | 37(17 to 69) | 0.65(0.30 to 1.21) | 48(23 to 84) | 0.41(0.20 to 0.73) | -1.03(-1.40 to -0.65) | 11938(4319 to 25188) | 219.68(75.62 to 469.11) | 24501(6740 to 55447) | 211.71(59.21 to 478.49) | -0.08(-0.31 to 0.14) |
| **American Samoa** | 0(0 to 0) | 1.29(0.62 to 2.41) | 0(0 to 0) | 1.38(0.77 to 2.32) | 0.28(-0.39 to 0.95) | 26(13 to 43) | 210.92(108.05 to 351.17) | 25(13 to 42) | 220.65(112.32 to 364.55) | 0.10(-0.15 to 0.35) |
| **Andorra** | 0(0 to 0) | 0.02(0.01 to 0.03) | 0(0 to 0) | 0.01(0.00 to 0.02) | -2.73(-3.46 to -2.00) | 17(2 to 47) | 112.59(16.82 to 302.32) | 28(3 to 79) | 135.67(17.44 to 378.74) | 0.75(0.41 to 1.10) |
| **Angola** | 37(18 to 69) | 1.65(0.78 to 3.05) | 1185(545 to 2260) | 18.04(8.32 to 34.16) | 8.10(7.24 to 8.97) | 7536(2787 to 15687) | 337.87(120.09 to 713.46) | 86553(43085 to 150990) | 1268.49(635.98 to 2200.00) | 4.37(3.75 to 4.99) |
| **Antigua and Barbuda** | 0(0 to 1) | 2.24(1.36 to 3.36) | 0(0 to 0) | 1.35(0.83 to 2.01) | -1.44(-1.89 to -0.99) | 36(19 to 62) | 221.93(116.11 to 384.05) | 46(20 to 92) | 188.68(81.43 to 374.61) | -0.60(-0.91 to -0.30) |
| **Argentina** | 90(62 to 127) | 1.13(0.78 to 1.58) | 132(81 to 201) | 1.11(0.67 to 1.68) | -0.04(-0.68 to 0.61) | 10870(5592 to 20201) | 136.11(69.87 to 253.43) | 17213(7763 to 33944) | 144.07(65.08 to 283.86) | 0.23(-0.20 to 0.66) |
| **Armenia** | 6(3 to 9) | 0.66(0.39 to 1.04) | 1(1 to 2) | 0.14(0.09 to 0.23) | -4.99(-5.92 to -4.05) | 605(331 to 1105) | 69.13(37.32 to 127.82) | 337(121 to 738) | 42.76(15.70 to 93.27) | -1.46(-2.19 to -0.72) |
| **Australia** | 48(30 to 71) | 1.06(0.66 to 1.60) | 25(16 to 38) | 0.41(0.25 to 0.62) | -3.24(-5.49 to -0.94) | 8664(3029 to 16437) | 192.51(68.06 to 364.34) | 11169(2177 to 25855) | 185.44(36.76 to 431.80) | -0.11(-0.51 to 0.30) |
| **Austria** | 19(12 to 27) | 0.96(0.61 to 1.34) | 8(5 to 11) | 0.39(0.24 to 0.55) | -2.69(-3.91 to -1.45) | 2975(1331 to 5854) | 148.26(66.86 to 290.57) | 2276(838 to 4710) | 113.51(43.19 to 232.91) | -0.85(-1.25 to -0.45) |
| **Azerbaijan** | 19(11 to 33) | 1.03(0.57 to 1.73) | 9(4 to 16) | 0.32(0.15 to 0.59) | -3.73(-4.11 to -3.35) | 2456(1183 to 4339) | 128.59(62.02 to 228.01) | 2169(730 to 4817) | 78.05(26.40 to 174.10) | -1.63(-1.82 to -1.44) |
| **Bahamas** | 5(3 to 7) | 6.41(3.95 to 9.43) | 8(5 to 11) | 7.04(4.19 to 10.72) | 0.48(-1.55 to 2.55) | 338(203 to 517) | 468.08(281.06 to 719.71) | 574(334 to 906) | 536.92(312.13 to 847.60) | 0.57(-1.22 to 2.40) |
| **Bahrain** | 1(0 to 1) | 0.66(0.33 to 1.22) | 3(1 to 5) | 0.80(0.39 to 1.42) | 0.55(-0.30 to 1.41) | 289(87 to 643) | 252.96(73.98 to 561.64) | 881(250 to 2032) | 265.27(76.33 to 611.14) | 0.23(0.05 to 0.41) |
| **Bangladesh** | 200(97 to 368) | 0.75(0.37 to 1.37) | 204(94 to 392) | 0.44(0.20 to 0.84) | -1.59(-2.04 to -1.14) | 74520(20113 to 148875) | 315.66(75.76 to 640.05) | 137639(26475 to 297679) | 301.39(56.64 to 652.16) | -0.06(-0.24 to 0.11) |
| **Barbados** | 3(2 to 5) | 4.38(2.60 to 6.54) | 3(1 to 4) | 3.96(2.15 to 6.30) | -0.15(-1.93 to 1.66) | 251(142 to 396) | 360.82(202.95 to 571.65) | 248(130 to 424) | 357.65(189.32 to 603.88) | 0.02(-1.36 to 1.42) |
| **Belarus** | 33(20 to 52) | 1.29(0.76 to 2.04) | 17(11 to 25) | 0.67(0.42 to 1.00) | -1.92(-3.66 to -0.16) | 3378(1714 to 6161) | 130.84(66.19 to 238.85) | 2653(962 to 5441) | 107.72(39.87 to 219.21) | -0.50(-1.33 to 0.34) |
| **Belgium** | 29(18 to 41) | 1.15(0.72 to 1.67) | 14(9 to 21) | 0.55(0.34 to 0.80) | -2.41(-2.93 to -1.89) | 4368(1825 to 8374) | 176.16(74.15 to 337.02) | 4584(1106 to 10197) | 178.85(44.41 to 397.12) | 0.03(-0.46 to 0.52) |
| **Belize** | 1(1 to 2) | 2.99(1.77 to 4.54) | 4(3 to 6) | 3.56(2.16 to 5.29) | 0.95(0.21 to 1.70) | 105(61 to 164) | 242.91(140.99 to 383.38) | 344(200 to 535) | 284.93(166.19 to 443.43) | 0.55(-0.16 to 1.26) |
| **Benin** | 16(7 to 30) | 1.40(0.61 to 2.64) | 104(48 to 194) | 3.48(1.63 to 6.36) | 3.09(2.01 to 4.17) | 2274(973 to 4363) | 210.22(86.87 to 409.81) | 10198(5294 to 17377) | 334.38(173.51 to 562.06) | 1.61(0.64 to 2.59) |
| **Bermuda** | 0(0 to 1) | 2.29(1.35 to 3.44) | 0(0 to 0) | 1.17(0.70 to 1.75) | -1.94(-2.38 to -1.50) | 45(22 to 80) | 253.13(122.14 to 449.68) | 28(10 to 59) | 200.35(77.78 to 418.96) | -0.63(-1.06 to -0.20) |
| **Bhutan** | 1(0 to 1) | 0.46(0.19 to 0.96) | 1(0 to 1) | 0.30(0.13 to 0.60) | -1.44(-2.24 to -0.62) | 206(71 to 448) | 156.50(48.62 to 351.72) | 275(84 to 602) | 133.62(39.96 to 294.13) | -0.54(-0.66 to -0.42) |
| **Bolivia (Plurinational State of)** | 43(19 to 82) | 2.77(1.20 to 5.20) | 63(29 to 118) | 2.02(0.94 to 3.76) | -0.99(-1.38 to -0.61) | 5546(2383 to 9715) | 367.39(153.15 to 645.56) | 10608(3952 to 19882) | 341.37(126.08 to 641.36) | -0.15(-0.69 to 0.39) |
| **Bosnia and Herzegovina** | 3(1 to 6) | 0.25(0.11 to 0.52) | 1(0 to 2) | 0.12(0.06 to 0.22) | -2.45(-2.91 to -1.99) | 1243(325 to 2848) | 106.60(27.75 to 244.80) | 763(144 to 1971) | 98.84(19.71 to 254.13) | -0.22(-0.35 to -0.10) |
| **Botswana** | 83(28 to 183) | 28.46(9.74 to 62.49) | 344(140 to 693) | 51.15(20.77 to 103.65) | 1.84(0.67 to 3.02) | 5961(2424 to 12039) | 1969.37(808.55 to 3966.22) | 21943(10373 to 41269) | 3235.43(1524.44 to 6114.45) | 1.55(0.64 to 2.46) |
| **Brazil** | 998(619 to 1538) | 2.51(1.58 to 3.83) | 1355(847 to 2042) | 2.32(1.43 to 3.53) | -0.29(-0.71 to 0.13) | 128800(55531 to 225795) | 331.39(138.19 to 584.51) | 154347(71436 to 276863) | 262.04(122.64 to 466.76) | -0.69(-1.00 to -0.38) |
| **Brunei Darussalam** | 0(0 to 0) | 0.32(0.14 to 0.61) | 0(0 to 0) | 0.17(0.09 to 0.30) | -2.11(-2.32 to -1.90) | 45(16 to 97) | 65.94(23.43 to 141.86) | 80(19 to 191) | 62.35(15.44 to 149.20) | -0.13(-0.23 to -0.03) |
| **Bulgaria** | 6(4 to 9) | 0.29(0.18 to 0.45) | 2(1 to 3) | 0.15(0.09 to 0.22) | -2.18(-2.65 to -1.69) | 2005(411 to 4708) | 94.90(20.23 to 222.46) | 1702(176 to 4162) | 111.78(12.68 to 274.25) | 0.54(0.27 to 0.81) |
| **Burkina Faso** | 302(121 to 641) | 16.09(6.36 to 34.24) | 177(83 to 329) | 3.28(1.58 to 6.03) | -4.97(-5.48 to -4.46) | 19459(8341 to 39604) | 1005.67(427.58 to 2057.00) | 15108(7728 to 26371) | 276.85(143.78 to 478.09) | -4.06(-4.53 to -3.60) |
| **Burundi** | 290(89 to 716) | 25.88(8.04 to 64.11) | 70(27 to 146) | 2.76(1.04 to 5.81) | -7.13(-8.27 to -5.98) | 20138(7109 to 45991) | 1731.72(619.71 to 3952.03) | 10192(4301 to 18563) | 368.58(153.47 to 672.31) | -4.94(-5.99 to -3.87) |
| **Cabo Verde** | 3(2 to 6) | 4.30(2.10 to 7.45) | 5(2 to 9) | 3.19(1.53 to 5.98) | -1.06(-1.57 to -0.54) | 278(147 to 460) | 361.38(191.68 to 601.13) | 470(232 to 839) | 312.45(153.93 to 559.59) | -0.50(-0.92 to -0.07) |
| **Cambodia** | 35(16 to 68) | 1.33(0.60 to 2.58) | 46(20 to 94) | 1.03(0.45 to 2.09) | -0.83(-1.19 to -0.46) | 3628(1776 to 6466) | 140.80(69.04 to 249.16) | 5761(2602 to 10247) | 128.00(57.65 to 227.64) | -0.31(-0.57 to -0.05) |
| **Cameroon** | 116(53 to 219) | 5.29(2.38 to 10.06) | 1054(501 to 1860) | 16.38(7.79 to 28.92) | 3.60(2.89 to 4.31) | 11523(5827 to 19730) | 511.16(255.26 to 876.58) | 73616(39626 to 122344) | 1092.20(587.86 to 1815.04) | 2.43(1.79 to 3.08) |
| **Canada** | 77(50 to 114) | 1.03(0.66 to 1.55) | 34(20 to 55) | 0.41(0.24 to 0.67) | -3.05(-4.34 to -1.75) | 13399(4641 to 25651) | 179.78(63.27 to 343.51) | 9230(2578 to 21955) | 111.72(31.98 to 265.15) | -1.44(-1.94 to -0.94) |
| **Central African Republic** | 122(42 to 273) | 19.32(6.52 to 43.62) | 267(124 to 499) | 22.43(10.45 to 41.61) | 0.36(-0.95 to 1.69) | 9415(3888 to 18891) | 1452.54(591.86 to 2940.12) | 18928(9605 to 32924) | 1529.31(779.79 to 2643.78) | 0.06(-1.22 to 1.36) |
| **Chad** | 45(21 to 85) | 3.65(1.73 to 6.90) | 185(90 to 326) | 5.40(2.63 to 9.55) | 1.14(0.19 to 2.11) | 4713(2314 to 8431) | 370.88(180.23 to 669.02) | 17623(9236 to 29413) | 498.72(260.10 to 831.97) | 0.98(0.40 to 1.56) |
| **Chile** | 42(25 to 66) | 1.18(0.71 to 1.85) | 33(21 to 50) | 0.68(0.43 to 1.04) | -1.53(-2.08 to -0.98) | 9565(2637 to 19358) | 264.54(72.44 to 535.11) | 11790(2165 to 28604) | 245.83(45.62 to 599.66) | -0.05(-0.44 to 0.35) |
| **China** | 3797(2157 to 6038) | 1.18(0.68 to 1.87) | 1254(768 to 1891) | 0.38(0.23 to 0.58) | -3.64(-3.87 to -3.41) | 749227(379533 to 1224412) | 234.37(117.48 to 383.28) | 438528(214153 to 729303) | 133.81(66.44 to 222.34) | -1.77(-1.93 to -1.60) |
| **Colombia** | 107(64 to 165) | 1.22(0.75 to 1.85) | 99(63 to 143) | 0.75(0.47 to 1.08) | -1.62(-2.43 to -0.80) | 12015(5499 to 21006) | 139.77(61.87 to 246.73) | 12273(5171 to 22507) | 92.55(39.09 to 169.64) | -1.35(-1.86 to -0.84) |
| **Comoros** | 2(1 to 5) | 2.10(0.70 to 4.36) | 6(3 to 12) | 3.08(1.29 to 5.94) | 1.16(-1.65 to 4.05) | 217(87 to 417) | 201.45(82.17 to 387.00) | 520(236 to 961) | 261.29(118.80 to 482.78) | 0.76(-1.34 to 2.91) |
| **Congo** | 121(42 to 270) | 24.68(8.68 to 54.81) | 286(128 to 541) | 21.26(9.60 to 40.07) | -0.51(-1.54 to 0.53) | 8826(3379 to 17982) | 1724.08(665.19 to 3488.37) | 20326(9691 to 36202) | 1488.65(710.06 to 2641.48) | -0.51(-1.31 to 0.29) |
| **Cook Islands** | 0(0 to 0) | 1.07(0.47 to 2.08) | 0(0 to 0) | 0.70(0.33 to 1.25) | -1.39(-1.87 to -0.90) | 10(4 to 18) | 219.49(98.25 to 390.08) | 9(4 to 17) | 207.00(87.62 to 389.61) | -0.20(-0.32 to -0.07) |
| **Costa Rica** | 12(8 to 17) | 1.56(0.99 to 2.24) | 25(15 to 38) | 1.89(1.14 to 2.92) | 0.36(-0.16 to 0.88) | 1583(726 to 2879) | 206.61(92.27 to 379.45) | 3360(1345 to 6475) | 255.08(103.17 to 490.03) | 0.58(0.26 to 0.90) |
| **Coted'Ivoire** | 937(332 to 2025) | 38.11(13.40 to 81.86) | 747(338 to 1308) | 12.56(5.60 to 22.29) | -3.74(-4.60 to -2.87) | 61156(23765 to 126672) | 2378.67(918.85 to 4906.37) | 54127(28469 to 88422) | 876.24(455.35 to 1444.85) | -3.39(-4.24 to -2.54) |
| **Croatia** | 15(10 to 21) | 1.21(0.77 to 1.70) | 3(2 to 5) | 0.35(0.22 to 0.50) | -4.09(-5.10 to -3.07) | 2178(1118 to 3797) | 177.72(91.63 to 309.38) | 1028(384 to 2188) | 109.21(42.01 to 230.82) | -1.59(-2.01 to -1.16) |
| **Cuba** | 109(62 to 162) | 3.42(1.97 to 5.06) | 48(27 to 75) | 1.92(1.07 to 3.02) | -2.04(-2.92 to -1.16) | 11677(5687 to 20304) | 372.34(178.08 to 657.40) | 6068(2528 to 12120) | 240.71(103.27 to 470.53) | -1.49(-2.09 to -0.89) |
| **Cyprus** | 2(1 to 4) | 1.01(0.44 to 1.94) | 1(1 to 2) | 0.33(0.16 to 0.58) | -3.39(-3.85 to -2.93) | 286(124 to 557) | 143.59(62.70 to 279.56) | 399(125 to 893) | 108.15(35.23 to 241.64) | -0.85(-1.24 to -0.46) |
| **Czechia** | 34(22 to 45) | 1.29(0.85 to 1.71) | 9(6 to 12) | 0.35(0.22 to 0.48) | -4.28(-4.88 to -3.67) | 5745(2783 to 9929) | 219.49(107.53 to 377.46) | 3664(1377 to 7446) | 150.61(58.68 to 304.95) | -1.19(-1.52 to -0.85) |
| **Democratic People's Republic of Korea** | 68(28 to 137) | 1.21(0.50 to 2.43) | 68(29 to 134) | 1.01(0.44 to 2.01) | -0.61(-0.86 to -0.37) | 12122(6439 to 20355) | 214.26(114.30 to 359.33) | 12309(6310 to 20660) | 184.87(94.85 to 310.24) | -0.45(-0.58 to -0.31) |
| **Democratic Republic of the Congo** | 1529(712 to 2878) | 20.58(9.62 to 38.55) | 654(325 to 1189) | 3.39(1.68 to 6.25) | -5.81(-6.80 to -4.81) | 117352(59097 to 200947) | 1511.32(760.92 to 2585.76) | 102468(43950 to 178693) | 509.32(217.68 to 884.71) | -3.39(-4.19 to -2.59) |
| **Denmark** | 15(10 to 20) | 1.09(0.73 to 1.49) | 4(3 to 6) | 0.34(0.22 to 0.47) | -3.79(-4.26 to -3.32) | 3056(1045 to 6184) | 228.28(79.69 to 460.28) | 2597(473 to 5770) | 200.34(38.29 to 447.19) | -0.36(-0.63 to -0.09) |
| **Djibouti** | 1(1 to 3) | 1.34(0.55 to 2.73) | 58(25 to 107) | 18.08(7.91 to 33.45) | 8.40(6.95 to 9.87) | 236(93 to 457) | 249.28(94.30 to 488.47) | 3784(1862 to 6586) | 1175.74(579.39 to 2044.54) | 5.07(4.06 to 6.10) |
| **Dominica** | 0(0 to 1) | 1.95(1.09 to 3.08) | 0(0 to 1) | 2.42(1.15 to 4.35) | 1.24(0.77 to 1.72) | 35(17 to 61) | 207.80(100.03 to 371.76) | 43(19 to 81) | 261.52(118.20 to 490.30) | 0.94(0.62 to 1.25) |
| **Dominican Republic** | 60(31 to 98) | 3.09(1.64 to 5.04) | 124(60 to 218) | 4.26(2.06 to 7.46) | 0.98(0.33 to 1.63) | 5829(2967 to 9637) | 302.97(152.77 to 508.25) | 11708(5661 to 20133) | 401.89(193.76 to 691.90) | 0.92(0.63 to 1.21) |
| **Ecuador** | 47(28 to 71) | 1.87(1.15 to 2.82) | 89(55 to 136) | 1.88(1.16 to 2.86) | -0.09(-0.49 to 0.31) | 5436(2753 to 9195) | 218.25(108.84 to 371.96) | 12712(5380 to 23107) | 267.98(113.26 to 487.32) | 0.75(0.20 to 1.29) |
| **Egypt** | 28(16 to 43) | 0.22(0.13 to 0.34) | 104(56 to 176) | 0.40(0.22 to 0.68) | 2.07(1.31 to 2.84) | 16790(6089 to 35123) | 132.85(47.03 to 280.19) | 40857(13265 to 88271) | 158.93(51.04 to 344.17) | 0.66(0.48 to 0.84) |
| **El Salvador** | 14(8 to 23) | 1.10(0.64 to 1.81) | 28(16 to 48) | 1.58(0.89 to 2.70) | 1.20(-0.32 to 2.75) | 1844(684 to 3664) | 144.30(52.61 to 287.30) | 3623(1319 to 7315) | 204.13(74.27 to 412.09) | 1.18(0.24 to 2.14) |
| **Equatorial Guinea** | 3(1 to 6) | 3.34(1.59 to 6.19) | 157(59 to 329) | 48.77(18.46 to 101.49) | 9.23(8.15 to 10.32) | 475(203 to 885) | 498.95(209.49 to 928.27) | 10398(4556 to 21309) | 3134.79(1377.13 to 6388.85) | 6.47(5.22 to 7.73) |
| **Eritrea** | 48(20 to 101) | 6.57(2.78 to 14.13) | 97(50 to 166) | 6.23(3.27 to 10.52) | -0.16(-0.79 to 0.47) | 4306(1993 to 8411) | 580.01(267.15 to 1140.02) | 8894(4753 to 14714) | 557.17(300.50 to 919.16) | -0.20(-0.43 to 0.03) |
| **Estonia** | 3(2 to 5) | 0.89(0.53 to 1.39) | 1(1 to 2) | 0.43(0.28 to 0.64) | -2.26(-2.93 to -1.59) | 691(207 to 1537) | 175.97(53.62 to 389.60) | 412(86 to 945) | 137.19(29.81 to 315.12) | -0.80(-1.24 to -0.36) |
| **Eswatini** | 19(11 to 31) | 9.40(5.20 to 15.04) | 246(93 to 510) | 85.18(31.86 to 177.34) | 7.38(6.27 to 8.50) | 1617(920 to 2462) | 793.36(448.39 to 1215.43) | 15856(6769 to 31152) | 5354.47(2288.34 to 10531.71) | 6.28(5.25 to 7.31) |
| **Ethiopia** | 1195(623 to 2043) | 11.37(5.92 to 19.46) | 1386(745 to 2346) | 5.72(3.06 to 9.75) | -2.13(-2.68 to -1.59) | 97790(55085 to 154843) | 908.38(510.91 to 1436.33) | 120018(67378 to 190190) | 473.08(264.05 to 746.48) | -2.21(-2.64 to -1.78) |
| **Fiji** | 3(2 to 5) | 1.50(0.85 to 2.45) | 4(2 to 7) | 1.73(0.95 to 2.95) | 0.63(-0.10 to 1.37) | 465(239 to 763) | 236.78(121.97 to 387.84) | 608(304 to 1009) | 266.34(133.25 to 441.76) | 0.42(0.02 to 0.82) |
| **Finland** | 21(14 to 29) | 1.62(1.04 to 2.27) | 8(5 to 11) | 0.71(0.46 to 0.98) | -2.57(-4.12 to -1.00) | 3662(1394 to 7045) | 281.13(110.58 to 537.13) | 3103(710 to 6674) | 270.12(64.47 to 581.08) | -0.14(-0.97 to 0.71) |
| **France** | 129(84 to 182) | 0.88(0.57 to 1.24) | 38(25 to 53) | 0.26(0.16 to 0.36) | -3.94(-5.15 to -2.72) | 26916(9612 to 57290) | 183.76(65.92 to 391.59) | 28298(4455 to 64233) | 193.11(31.90 to 437.94) | 0.11(-0.28 to 0.49) |
| **Gabon** | 14(6 to 29) | 7.37(3.22 to 14.79) | 81(36 to 148) | 18.97(8.39 to 34.54) | 3.15(1.64 to 4.68) | 1448(682 to 2626) | 708.69(331.77 to 1289.54) | 6083(3037 to 10250) | 1371.54(686.53 to 2308.45) | 2.20(1.26 to 3.16) |
| **Gambia** | 1(0 to 2) | 0.53(0.20 to 1.17) | 38(16 to 73) | 7.64(3.17 to 14.63) | 8.98(7.98 to 9.98) | 419(94 to 1059) | 208.82(43.23 to 525.96) | 3165(1398 to 5672) | 609.02(272.84 to 1073.75) | 3.66(2.82 to 4.51) |
| **Georgia** | 7(4 to 11) | 0.50(0.29 to 0.79) | 1(1 to 2) | 0.15(0.09 to 0.24) | -3.73(-6.81 to -0.55) | 805(419 to 1411) | 57.75(30.00 to 101.72) | 290(114 to 644) | 34.57(13.91 to 75.45) | -1.78(-3.42 to -0.11) |
| **Germany** | 127(84 to 185) | 0.65(0.42 to 0.95) | 42(28 to 60) | 0.24(0.16 to 0.35) | -3.22(-4.58 to -1.83) | 31124(9695 to 60470) | 156.63(50.06 to 303.78) | 31627(5289 to 73095) | 180.15(32.30 to 418.22) | 0.35(-0.34 to 1.03) |
| **Ghana** | 140(62 to 269) | 4.34(1.92 to 8.33) | 624(306 to 1126) | 7.46(3.67 to 13.40) | 2.04(1.36 to 2.72) | 12342(6072 to 22179) | 371.34(180.98 to 667.55) | 46532(25302 to 78481) | 541.30(295.54 to 909.84) | 1.46(0.95 to 1.98) |
| **Greece** | 12(7 to 18) | 0.49(0.30 to 0.72) | 6(4 to 9) | 0.26(0.16 to 0.39) | -2.28(-3.49 to -1.05) | 4836(1204 to 11534) | 190.36(48.19 to 453.06) | 4674(674 to 11676) | 208.40(32.60 to 524.93) | 0.32(-0.39 to 1.04) |
| **Greenland** | 2(1 to 3) | 11.97(6.14 to 22.20) | 1(0 to 1) | 4.27(2.14 to 7.61) | -3.22(-3.49 to -2.96) | 152(76 to 265) | 995.09(495.12 to 1724.09) | 72(26 to 145) | 550.61(198.48 to 1111.06) | -1.84(-2.17 to -1.51) |
| **Grenada** | 1(0 to 1) | 2.53(1.48 to 3.83) | 0(0 to 1) | 1.49(0.87 to 2.31) | -2.03(-4.01 to 0.00) | 48(25 to 82) | 246.02(127.70 to 428.08) | 51(22 to 101) | 199.65(85.39 to 394.03) | -0.73(-1.99 to 0.55) |
| **Guam** | 1(0 to 1) | 1.66(0.77 to 3.15) | 1(0 to 1) | 1.61(0.98 to 2.46) | -0.20(-2.07 to 1.71) | 88(44 to 146) | 247.85(121.92 to 410.41) | 92(46 to 157) | 255.19(128.07 to 436.06) | 0.09(-0.67 to 0.86) |
| **Guatemala** | 25(15 to 38) | 1.40(0.84 to 2.15) | 40(23 to 64) | 0.90(0.53 to 1.43) | -1.46(-3.50 to 0.63) | 2939(1198 to 5666) | 174.23(67.64 to 342.28) | 6507(2019 to 13846) | 152.24(45.53 to 326.73) | -0.44(-1.78 to 0.92) |
| **Guinea** | 36(17 to 66) | 2.69(1.29 to 4.94) | 185(87 to 332) | 6.27(2.94 to 11.19) | 2.75(2.04 to 3.45) | 4090(1892 to 7637) | 305.74(139.46 to 574.41) | 15971(8049 to 27367) | 521.99(263.18 to 888.37) | 1.78(1.24 to 2.32) |
| **Guinea-Bissau** | 12(6 to 21) | 5.12(2.44 to 9.17) | 53(23 to 105) | 11.01(4.83 to 22.05) | 2.52(1.95 to 3.09) | 1003(510 to 1708) | 433.68(221.68 to 734.65) | 3846(1859 to 7099) | 779.42(373.91 to 1448.17) | 1.92(1.41 to 2.43) |
| **Guyana** | 12(7 to 18) | 5.77(3.45 to 8.67) | 17(10 to 27) | 8.30(4.74 to 13.04) | 1.24(0.36 to 2.12) | 992(545 to 1595) | 483.35(265.13 to 781.74) | 1421(779 to 2306) | 691.71(380.71 to 1122.76) | 1.19(0.33 to 2.06) |
| **Haiti** | 294(142 to 526) | 19.67(9.51 to 35.38) | 445(220 to 761) | 12.54(6.24 to 21.41) | -1.36(-2.01 to -0.70) | 20025(10347 to 34368) | 1307.68(676.31 to 2253.36) | 33561(17776 to 54520) | 938.76(498.61 to 1523.00) | -1.02(-1.54 to -0.50) |
| **Honduras** | 42(22 to 73) | 3.99(2.13 to 6.87) | 103(44 to 201) | 3.65(1.58 to 7.09) | -0.65(-3.05 to 1.82) | 3143(1681 to 5346) | 298.14(160.47 to 502.20) | 9450(4190 to 17324) | 335.92(148.03 to 613.99) | 0.42(-1.09 to 1.95) |
| **Hungary** | 32(19 to 48) | 1.21(0.73 to 1.84) | 7(4 to 11) | 0.33(0.20 to 0.51) | -4.09(-5.09 to -3.07) | 4225(2043 to 7773) | 162.52(78.92 to 297.86) | 2444(744 to 5266) | 106.75(35.14 to 225.17) | -1.40(-1.75 to -1.05) |
| **Iceland** | 0(0 to 1) | 0.54(0.32 to 0.80) | 0(0 to 0) | 0.22(0.13 to 0.33) | -2.83(-4.09 to -1.56) | 49(26 to 86) | 74.92(40.39 to 132.60) | 41(19 to 84) | 51.55(23.91 to 104.53) | -1.15(-1.78 to -0.51) |
| **India** | 2163(1195 to 3541) | 1.05(0.58 to 1.71) | 3105(1905 to 4677) | 0.83(0.51 to 1.24) | -0.69(-1.16 to -0.22) | 387537(154353 to 713186) | 195.12(74.93 to 362.15) | 683970(234409 to 1274361) | 182.35(61.99 to 340.02) | -0.09(-0.36 to 0.19) |
| **Indonesia** | 188(105 to 301) | 0.37(0.21 to 0.59) | 222(127 to 357) | 0.30(0.17 to 0.48) | -0.73(-1.50 to 0.06) | 38277(17970 to 69010) | 79.44(36.56 to 145.90) | 64434(26355 to 126922) | 85.15(35.14 to 167.15) | 0.23(-0.05 to 0.51) |
| **Iran (Islamic Republic of)** | 130(78 to 185) | 0.98(0.59 to 1.40) | 212(129 to 300) | 0.96(0.57 to 1.36) | -0.04(-0.49 to 0.41) | 33439(12019 to 69256) | 274.40(92.95 to 579.65) | 70714(20294 to 158045) | 296.35(89.87 to 652.86) | 0.34(0.23 to 0.44) |
| **Iraq** | 86(45 to 138) | 2.16(1.16 to 3.49) | 379(179 to 670) | 3.62(1.72 to 6.40) | 1.94(0.75 to 3.13) | 11399(5808 to 19025) | 293.93(146.81 to 498.86) | 37173(19150 to 61160) | 355.20(183.23 to 584.80) | 0.68(0.08 to 1.27) |
| **Ireland** | 2(2 to 4) | 0.27(0.17 to 0.43) | 1(1 to 2) | 0.11(0.07 to 0.17) | -2.72(-4.01 to -1.42) | 852(229 to 2002) | 97.55(25.90 to 229.71) | 1627(184 to 4141) | 137.67(16.41 to 348.88) | 1.19(0.77 to 1.60) |
| **Israel** | 14(9 to 20) | 1.14(0.72 to 1.67) | 10(6 to 15) | 0.45(0.28 to 0.66) | -2.77(-4.44 to -1.08) | 3050(987 to 6122) | 252.20(80.79 to 506.34) | 5107(942 to 12327) | 229.47(42.73 to 553.73) | -0.40(-0.73 to -0.06) |
| **Italy** | 95(63 to 132) | 0.67(0.44 to 0.92) | 31(22 to 43) | 0.25(0.17 to 0.33) | -3.37(-4.41 to -2.33) | 30606(8296 to 65261) | 212.87(58.49 to 452.93) | 30188(4381 to 66105) | 242.98(38.98 to 531.91) | 0.53(0.05 to 1.02) |
| **Jamaica** | 29(17 to 42) | 4.98(3.09 to 7.36) | 88(50 to 139) | 11.26(6.45 to 17.81) | 2.84(1.13 to 4.58) | 2176(1309 to 3251) | 369.22(224.71 to 553.97) | 6088(3626 to 9228) | 778.26(463.41 to 1180.20) | 2.44(0.89 to 4.00) |
| **Japan** | 54(32 to 85) | 0.17(0.10 to 0.26) | 26(16 to 41) | 0.11(0.06 to 0.17) | -1.25(-1.86 to -0.63) | 22379(6114 to 48442) | 70.62(19.43 to 152.89) | 20824(3589 to 50903) | 84.21(15.32 to 206.81) | 0.64(0.42 to 0.86) |
| **Jordan** | 13(7 to 23) | 1.62(0.81 to 2.82) | 29(13 to 52) | 0.92(0.43 to 1.67) | -1.83(-2.61 to -1.05) | 2052(1027 to 3521) | 259.99(126.75 to 454.22) | 5846(2622 to 10599) | 191.53(85.12 to 348.82) | -0.95(-1.22 to -0.68) |
| **Kazakhstan** | 84(50 to 133) | 2.05(1.22 to 3.23) | 38(23 to 58) | 0.76(0.45 to 1.17) | -3.17(-4.01 to -2.32) | 6904(4055 to 10920) | 167.67(98.33 to 264.92) | 4599(2151 to 8443) | 92.55(43.28 to 169.80) | -1.87(-2.56 to -1.19) |
| **Kenya** | 1039(445 to 2014) | 23.72(10.11 to 45.80) | 1703(898 to 2878) | 14.44(7.48 to 24.76) | -1.47(-2.51 to -0.42) | 72315(33930 to 134265) | 1571.23(736.25 to 2899.70) | 129759(77097 to 203132) | 1059.13(622.66 to 1671.08) | -1.26(-2.11 to -0.39) |
| **Kiribati** | 0(0 to 1) | 2.10(1.19 to 3.49) | 0(0 to 0) | 0.93(0.58 to 1.44) | -2.56(-3.72 to -1.40) | 66(36 to 104) | 347.83(188.82 to 543.24) | 81(38 to 133) | 252.05(119.28 to 413.59) | -1.14(-1.65 to -0.63) |
| **Kuwait** | 1(1 to 2) | 0.35(0.22 to 0.51) | 4(3 to 6) | 0.29(0.18 to 0.44) | -1.15(-5.88 to 3.81) | 819(252 to 1806) | 199.83(60.03 to 442.93) | 3002(797 to 6940) | 190.58(53.80 to 437.36) | -0.23(-0.93 to 0.46) |
| **Kyrgyzstan** | 21(12 to 34) | 2.16(1.29 to 3.46) | 13(8 to 19) | 0.73(0.46 to 1.12) | -3.33(-4.15 to -2.52) | 2052(1090 to 3436) | 207.83(109.87 to 347.48) | 2424(802 to 4926) | 139.78(46.14 to 283.92) | -1.19(-1.67 to -0.70) |
| **Lao People's Democratic Republic** | 30(10 to 63) | 3.11(1.09 to 6.44) | 35(16 to 69) | 1.78(0.78 to 3.48) | -1.81(-2.03 to -1.60) | 2635(1216 to 4936) | 270.13(126.62 to 501.14) | 3343(1623 to 6052) | 166.95(81.25 to 301.51) | -1.55(-1.74 to -1.37) |
| **Latvia** | 10(6 to 15) | 1.42(0.86 to 2.23) | 8(5 to 11) | 1.75(1.11 to 2.50) | 0.97(-0.42 to 2.38) | 1576(544 to 3067) | 234.48(82.08 to 456.04) | 1189(419 to 2279) | 276.65(96.95 to 535.74) | 0.49(-0.03 to 1.02) |
| **Lebanon** | 108(69 to 145) | 14.39(9.31 to 19.36) | 13(7 to 23) | 0.82(0.43 to 1.50) | -7.35(-11.26 to -3.26) | 8651(5551 to 11836) | 1147.36(739.91 to 1571.81) | 4647(1230 to 10745) | 299.02(80.28 to 694.24) | -2.98(-4.04 to -1.91) |
| **Lesotho** | 52(26 to 93) | 14.22(7.06 to 25.96) | 446(205 to 831) | 100.30(45.67 to 187.41) | 6.72(3.34 to 10.22) | 4303(2285 to 7286) | 1171.22(617.11 to 1995.32) | 28175(14008 to 49550) | 6157.48(3040.44 to 10842.73) | 5.26(3.77 to 6.77) |
| **Liberia** | 14(7 to 26) | 2.45(1.24 to 4.82) | 77(35 to 139) | 6.08(2.76 to 11.04) | 1.91(-0.50 to 4.38) | 1930(866 to 3511) | 353.49(152.87 to 652.33) | 7260(3497 to 12452) | 557.97(268.97 to 953.63) | 0.85(-0.94 to 2.67) |
| **Libya** | 7(3 to 14) | 0.83(0.37 to 1.59) | 22(8 to 55) | 1.06(0.41 to 2.67) | 0.55(-4.56 to 5.92) | 1926(719 to 4054) | 231.48(82.33 to 489.80) | 6358(2596 to 12394) | 313.77(129.18 to 608.58) | 0.88(-0.58 to 2.36) |
| **Lithuania** | 11(7 to 17) | 1.19(0.72 to 1.87) | 5(3 to 7) | 0.84(0.54 to 1.19) | -1.08(-2.04 to -0.10) | 1781(604 to 3718) | 189.53(64.74 to 394.65) | 1260(291 to 2730) | 204.16(48.28 to 443.24) | 0.27(-0.19 to 0.73) |
| **Luxembourg** | 1(1 to 2) | 1.07(0.66 to 1.56) | 1(0 to 1) | 0.37(0.23 to 0.54) | -3.43(-5.15 to -1.67) | 201(73 to 420) | 200.34(73.47 to 416.26) | 244(56 to 532) | 152.48(37.20 to 331.73) | -0.81(-1.26 to -0.36) |
| **Madagascar** | 12(5 to 22) | 0.41(0.19 to 0.74) | 234(100 to 433) | 3.69(1.57 to 6.77) | 7.55(7.11 to 8.00) | 5135(1538 to 10817) | 201.11(54.44 to 429.12) | 26089(11867 to 46697) | 391.53(177.26 to 695.53) | 2.15(1.91 to 2.38) |
| **Malawi** | 773(302 to 1551) | 39.13(15.27 to 78.51) | 896(374 to 1740) | 22.64(9.43 to 44.03) | -1.81(-2.71 to -0.91) | 51766(22508 to 98234) | 2508.61(1089.48 to 4766.17) | 63399(31043 to 111652) | 1524.36(739.08 to 2692.56) | -1.72(-2.31 to -1.14) |
| **Malaysia** | 20(10 to 34) | 0.45(0.23 to 0.75) | 39(21 to 64) | 0.46(0.25 to 0.76) | 0.08(-0.57 to 0.73) | 4023(1636 to 8262) | 91.35(36.14 to 190.11) | 8263(3002 to 17547) | 97.53(35.39 to 207.39) | 0.20(-0.06 to 0.46) |
| **Maldives** | 0(0 to 0) | 0.41(0.19 to 0.78) | 0(0 to 0) | 0.16(0.09 to 0.28) | -3.06(-3.56 to -2.54) | 44(16 to 96) | 96.32(34.24 to 215.35) | 79(25 to 169) | 66.88(21.07 to 142.98) | -1.15(-1.29 to -1.00) |
| **Mali** | 65(32 to 119) | 3.42(1.69 to 6.28) | 340(178 to 603) | 7.05(3.71 to 12.42) | 2.39(1.33 to 3.46) | 6647(3514 to 11281) | 347.70(183.40 to 590.35) | 28571(16152 to 45584) | 565.90(320.65 to 900.58) | 1.66(1.09 to 2.22) |
| **Malta** | 1(0 to 1) | 0.74(0.45 to 1.08) | 0(0 to 1) | 0.35(0.22 to 0.53) | -1.95(-4.02 to 0.17) | 125(50 to 263) | 131.25(52.94 to 273.89) | 111(33 to 247) | 114.37(35.43 to 252.83) | -0.52(-1.34 to 0.32) |
| **Marshall Islands** | 0(0 to 0) | 2.38(1.09 to 4.44) | 0(0 to 1) | 3.02(1.53 to 5.29) | 0.70(0.40 to 1.00) | 29(15 to 47) | 295.82(155.12 to 482.92) | 49(27 to 79) | 333.52(182.49 to 539.44) | 0.34(0.18 to 0.50) |
| **Mauritania** | 6(2 to 12) | 1.28(0.51 to 2.56) | 13(5 to 28) | 1.23(0.50 to 2.49) | -0.13(-0.39 to 0.12) | 875(382 to 1632) | 187.40(79.13 to 354.65) | 1934(775 to 3786) | 182.96(71.08 to 362.81) | -0.01(-0.16 to 0.14) |
| **Mauritius** | 2(1 to 2) | 0.53(0.31 to 0.83) | 2(1 to 3) | 0.53(0.32 to 0.79) | -0.04(-1.45 to 1.40) | 385(126 to 871) | 128.61(41.49 to 292.16) | 421(131 to 969) | 130.21(40.82 to 298.58) | 0.08(-0.62 to 0.79) |
| **Mexico** | 235(150 to 354) | 1.09(0.71 to 1.61) | 628(390 to 960) | 1.80(1.12 to 2.75) | 1.51(0.75 to 2.27) | 36449(12992 to 66595) | 174.87(58.70 to 323.26) | 92409(33655 to 182567) | 262.95(96.68 to 518.36) | 1.39(0.92 to 1.85) |
| **Micronesia (Federated States of)** | 1(0 to 1) | 2.96(1.29 to 5.73) | 1(0 to 2) | 3.91(1.99 to 7.05) | 0.93(0.77 to 1.09) | 78(42 to 133) | 339.64(180.66 to 571.91) | 101(55 to 167) | 391.17(217.14 to 643.72) | 0.46(0.37 to 0.55) |
| **Monaco** | 0(0 to 0) | 0.06(0.03 to 0.12) | 0(0 to 0) | 0.04(0.02 to 0.08) | -1.13(-1.35 to -0.90) | 10(1 to 26) | 129.06(19.35 to 350.32) | 12(1 to 33) | 156.87(18.72 to 448.52) | 0.65(0.59 to 0.71) |
| **Mongolia** | 9(4 to 17) | 1.90(0.89 to 3.51) | 6(3 to 11) | 0.73(0.36 to 1.31) | -2.98(-3.92 to -2.03) | 1157(471 to 2196) | 241.12(97.33 to 457.24) | 1492(391 to 3025) | 169.49(44.94 to 344.03) | -1.23(-1.44 to -1.02) |
| **Montenegro** | 2(1 to 3) | 1.09(0.56 to 1.92) | 1(0 to 2) | 0.59(0.30 to 1.04) | -1.67(-2.71 to -0.62) | 220(96 to 410) | 140.74(61.21 to 262.58) | 196(58 to 448) | 131.92(39.95 to 300.24) | -0.09(-0.53 to 0.36) |
| **Morocco** | 17(9 to 28) | 0.27(0.15 to 0.46) | 50(24 to 95) | 0.51(0.25 to 0.97) | 2.00(1.52 to 2.48) | 13523(3569 to 30961) | 226.87(56.76 to 521.62) | 25422(6126 to 59872) | 259.90(63.05 to 611.97) | 0.62(0.40 to 0.83) |
| **Mozambique** | 132(68 to 229) | 4.41(2.27 to 7.66) | 2645(1429 to 4214) | 40.82(22.34 to 64.22) | 7.32(6.81 to 7.83) | 13843(6985 to 23346) | 455.88(228.27 to 771.36) | 174484(97620 to 273030) | 2615.58(1482.66 to 4043.14) | 5.75(5.44 to 6.06) |
| **Myanmar** | 121(56 to 226) | 1.10(0.52 to 2.04) | 168(73 to 320) | 1.12(0.48 to 2.12) | 0.07(-0.91 to 1.06) | 12918(6361 to 22476) | 121.38(59.69 to 209.75) | 16478(8110 to 28428) | 109.34(53.91 to 188.48) | -0.40(-1.06 to 0.27) |
| **Namibia** | 31(16 to 53) | 9.46(4.87 to 16.47) | 138(62 to 265) | 22.70(10.13 to 43.79) | 2.67(1.60 to 3.76) | 2383(1314 to 3881) | 721.13(397.55 to 1182.62) | 8955(4425 to 16174) | 1445.60(713.18 to 2615.33) | 2.16(1.34 to 2.99) |
| **Nauru** | 0(0 to 0) | 3.05(1.02 to 6.30) | 0(0 to 0) | 3.78(1.52 to 7.52) | 0.68(0.56 to 0.81) | 9(4 to 14) | 350.04(167.46 to 593.38) | 12(6 to 20) | 404.42(195.13 to 703.24) | 0.45(0.35 to 0.54) |
| **Nepal** | 18(8 to 36) | 0.38(0.17 to 0.75) | 55(24 to 109) | 0.61(0.27 to 1.19) | 1.56(0.92 to 2.22) | 8038(2369 to 16734) | 181.99(50.43 to 384.16) | 19729(5007 to 42429) | 222.81(54.30 to 482.43) | 0.75(0.55 to 0.95) |
| **Netherlands** | 24(15 to 34) | 0.59(0.38 to 0.86) | 11(7 to 16) | 0.30(0.19 to 0.44) | -2.27(-3.56 to -0.97) | 7344(2016 to 14649) | 182.11(51.00 to 362.25) | 6492(1263 to 15270) | 173.60(35.18 to 406.24) | -0.21(-0.55 to 0.14) |
| **New Zealand** | 9(6 to 13) | 1.00(0.62 to 1.45) | 6(4 to 9) | 0.53(0.32 to 0.76) | -2.52(-2.98 to -2.05) | 2130(655 to 4282) | 234.98(72.46 to 472.18) | 2326(554 to 5030) | 195.22(47.20 to 424.42) | -0.65(-1.31 to 0.01) |
| **Nicaragua** | 12(6 to 20) | 1.29(0.71 to 2.12) | 15(8 to 26) | 0.83(0.46 to 1.44) | -1.38(-1.98 to -0.77) | 1468(664 to 2677) | 165.17(70.71 to 307.85) | 2754(864 to 5606) | 152.00(47.21 to 310.14) | -0.27(-0.61 to 0.08) |
| **Niger** | 40(18 to 74) | 2.33(1.09 to 4.35) | 125(58 to 248) | 2.41(1.15 to 4.63) | -0.03(-0.60 to 0.54) | 4924(2272 to 9014) | 293.90(132.79 to 545.35) | 14557(6691 to 26980) | 286.81(130.84 to 528.05) | -0.07(-0.51 to 0.38) |
| **Nigeria** | 748(427 to 1194) | 3.94(2.27 to 6.27) | 3931(2096 to 6354) | 8.15(4.37 to 13.01) | 2.35(1.47 to 3.24) | 70807(40869 to 109781) | 364.13(210.61 to 565.26) | 277524(162819 to 428183) | 551.01(324.55 to 842.93) | 1.35(0.70 to 2.01) |
| **Niue** | 0(0 to 0) | 1.69(0.71 to 3.48) | 0(0 to 0) | 2.93(1.36 to 5.47) | 1.57(0.79 to 2.36) | 1(1 to 2) | 263.27(125.57 to 457.05) | 1(1 to 2) | 352.50(176.64 to 607.54) | 0.98(0.72 to 1.24) |
| **North Macedonia** | 4(2 to 6) | 0.71(0.38 to 1.24) | 1(1 to 3) | 0.27(0.14 to 0.49) | -3.03(-4.26 to -1.79) | 593(249 to 1161) | 116.33(48.85 to 227.72) | 590(142 to 1462) | 106.20(26.78 to 261.03) | -0.19(-0.45 to 0.07) |
| **Northern Mariana Islands** | 0(0 to 1) | 3.33(1.53 to 6.35) | 0(0 to 0) | 2.20(1.20 to 3.73) | -0.96(-1.45 to -0.46) | 46(24 to 77) | 325.16(169.08 to 539.61) | 31(16 to 51) | 276.18(145.21 to 453.71) | -0.42(-0.71 to -0.12) |
| **Norway** | 6(4 to 9) | 0.58(0.37 to 0.82) | 5(3 to 7) | 0.41(0.26 to 0.58) | -1.77(-3.06 to -0.47) | 1909(501 to 3915) | 179.09(47.74 to 366.54) | 2132(450 to 4776) | 174.67(38.53 to 390.04) | -0.08(-0.55 to 0.39) |
| **Oman** | 1(0 to 1) | 0.21(0.11 to 0.39) | 2(1 to 4) | 0.22(0.12 to 0.34) | 0.13(-0.31 to 0.57) | 592(177 to 1314) | 183.72(52.60 to 408.68) | 2175(508 to 5352) | 205.82(48.94 to 503.12) | 0.45(0.29 to 0.60) |
| **Pakistan** | 220(111 to 387) | 0.89(0.45 to 1.55) | 659(283 to 1343) | 1.07(0.46 to 2.20) | 0.63(0.44 to 0.82) | 40934(16822 to 77898) | 178.52(69.48 to 347.18) | 110070(44234 to 207210) | 182.94(71.20 to 348.54) | 0.16(-0.04 to 0.37) |
| **Palau** | 0(0 to 0) | 4.32(1.81 to 8.71) | 0(0 to 0) | 5.15(2.31 to 9.44) | 0.55(0.42 to 0.67) | 18(9 to 32) | 427.91(208.75 to 756.35) | 17(9 to 30) | 502.04(249.22 to 864.24) | 0.52(0.38 to 0.66) |
| **Palestine** | 3(1 to 5) | 0.62(0.28 to 1.20) | 21(11 to 36) | 1.61(0.82 to 2.78) | 3.20(2.69 to 3.71) | 1569(326 to 3410) | 379.20(73.46 to 821.88) | 4996(1545 to 10225) | 391.62(117.68 to 805.64) | 0.17(-0.04 to 0.38) |
| **Panama** | 8(5 to 13) | 1.31(0.77 to 2.06) | 19(11 to 28) | 1.75(1.07 to 2.65) | 1.10(0.14 to 2.07) | 908(416 to 1765) | 151.02(67.51 to 297.69) | 2068(986 to 3818) | 193.51(92.48 to 356.97) | 0.88(0.24 to 1.53) |
| **Papua New Guinea** | 20(7 to 43) | 2.10(0.76 to 4.38) | 139(68 to 249) | 5.38(2.64 to 9.56) | 3.04(2.28 to 3.81) | 2898(1363 to 4966) | 298.20(140.28 to 507.80) | 13539(7510 to 21901) | 516.22(287.45 to 831.57) | 1.77(1.38 to 2.16) |
| **Paraguay** | 9(5 to 17) | 0.97(0.51 to 1.71) | 24(13 to 44) | 1.28(0.66 to 2.29) | 1.00(0.28 to 1.73) | 1129(491 to 2166) | 120.25(50.60 to 234.40) | 3419(1250 to 6819) | 179.82(65.29 to 360.90) | 1.38(0.92 to 1.84) |
| **Peru** | 103(58 to 163) | 1.90(1.10 to 2.99) | 126(73 to 207) | 1.31(0.75 to 2.14) | -1.25(-2.59 to 0.12) | 11499(6044 to 18238) | 215.53(111.85 to 343.62) | 17102(8435 to 28837) | 176.76(87.22 to 297.89) | -0.56(-0.98 to -0.12) |
| **Philippines** | 592(350 to 897) | 3.79(2.27 to 5.71) | 505(309 to 779) | 1.75(1.08 to 2.69) | -2.42(-2.85 to -1.99) | 46772(28062 to 70132) | 295.75(179.06 to 442.42) | 46491(27562 to 71252) | 159.33(94.78 to 244.14) | -2.00(-2.29 to -1.70) |
| **Poland** | 38(23 to 60) | 0.39(0.23 to 0.62) | 11(7 to 17) | 0.12(0.07 to 0.19) | -3.69(-4.41 to -2.97) | 5366(2393 to 9698) | 55.98(24.84 to 101.08) | 3991(1049 to 8818) | 42.68(11.89 to 93.81) | -0.91(-1.43 to -0.39) |
| **Portugal** | 37(24 to 50) | 1.45(0.96 to 1.96) | 14(9 to 18) | 0.55(0.37 to 0.73) | -3.04(-4.51 to -1.55) | 6738(2541 to 14392) | 266.79(100.97 to 569.58) | 5653(1173 to 13190) | 232.97(50.89 to 543.66) | -0.35(-0.73 to 0.03) |
| **Puerto Rico** | 48(30 to 69) | 5.02(3.18 to 7.18) | 17(10 to 26) | 2.31(1.37 to 3.51) | -2.46(-3.63 to -1.27) | 3532(2203 to 5085) | 370.13(231.04 to 532.70) | 1639(851 to 2723) | 220.99(116.09 to 365.14) | -1.69(-2.75 to -0.62) |
| **Qatar** | 0(0 to 0) | 0.23(0.13 to 0.36) | 1(1 to 2) | 0.24(0.12 to 0.42) | 0.22(-0.96 to 1.41) | 168(43 to 375) | 209.71(53.37 to 466.14) | 1237(278 to 2966) | 206.06(48.65 to 489.05) | -0.06(-0.22 to 0.11) |
| **Republic of Korea** | 63(34 to 105) | 0.50(0.27 to 0.83) | 27(13 to 48) | 0.22(0.11 to 0.39) | -2.54(-3.11 to -1.97) | 10912(4124 to 22566) | 85.77(32.31 to 177.09) | 8540(2088 to 19510) | 71.89(18.14 to 164.41) | -0.53(-0.86 to -0.20) |
| **Republic of Moldova** | 16(10 to 26) | 1.43(0.83 to 2.28) | 7(5 to 11) | 0.74(0.49 to 1.08) | -2.41(-3.68 to -1.12) | 1794(882 to 3113) | 158.68(77.48 to 276.05) | 1262(446 to 2620) | 128.91(46.56 to 266.01) | -0.74(-1.21 to -0.26) |
| **Romania** | 67(42 to 101) | 1.19(0.75 to 1.80) | 16(10 to 24) | 0.39(0.24 to 0.59) | -3.52(-4.98 to -2.03) | 8054(4263 to 13404) | 143.40(76.04 to 238.62) | 5375(1601 to 11468) | 128.40(40.28 to 272.63) | -0.23(-0.56 to 0.10) |
| **Russian Federation** | 1068(666 to 1639) | 2.81(1.74 to 4.34) | 854(573 to 1204) | 2.22(1.47 to 3.18) | -0.53(-1.81 to 0.76) | 100526(55896 to 160239) | 264.68(146.66 to 423.95) | 77394(42514 to 124219) | 208.05(113.31 to 338.10) | -0.81(-1.80 to 0.20) |
| **Rwanda** | 181(81 to 422) | 12.32(5.39 to 29.72) | 226(97 to 420) | 7.25(3.11 to 13.59) | -1.72(-2.52 to -0.91) | 14793(7392 to 30404) | 982.13(482.28 to 2054.23) | 20595(10317 to 34530) | 638.59(319.62 to 1071.77) | -1.44(-2.23 to -0.64) |
| **Saint Kitts and Nevis** | 0(0 to 0) | 2.42(1.39 to 3.74) | 0(0 to 1) | 1.93(1.05 to 3.23) | -0.88(-2.04 to 0.29) | 26(14 to 46) | 261.40(129.31 to 469.39) | 39(17 to 78) | 244.25(105.24 to 484.20) | -0.42(-1.41 to 0.58) |
| **Saint Lucia** | 1(1 to 2) | 3.91(2.35 to 5.94) | 1(1 to 2) | 2.70(1.51 to 4.19) | -1.23(-2.13 to -0.33) | 113(64 to 181) | 328.29(185.23 to 531.31) | 128(63 to 233) | 280.85(140.57 to 503.86) | -0.59(-1.45 to 0.28) |
| **Saint Vincent and the Grenadines** | 2(1 to 3) | 7.44(4.52 to 11.16) | 2(1 to 3) | 6.33(3.73 to 9.73) | -0.75(-2.66 to 1.20) | 148(86 to 226) | 545.36(318.81 to 833.70) | 138(78 to 216) | 499.98(283.97 to 782.96) | -0.51(-2.19 to 1.19) |
| **Samoa** | 1(0 to 2) | 2.02(0.83 to 4.35) | 1(1 to 2) | 2.93(1.50 to 5.27) | 1.19(1.01 to 1.37) | 88(45 to 154) | 244.00(123.86 to 422.29) | 139(77 to 234) | 291.36(163.26 to 486.02) | 0.54(0.39 to 0.69) |
| **San Marino** | 0(0 to 0) | 0.50(0.23 to 0.98) | 0(0 to 0) | 0.23(0.09 to 0.48) | -2.60(-3.11 to -2.09) | 10(2 to 23) | 153.68(37.86 to 372.11) | 12(2 to 34) | 171.29(26.74 to 459.95) | 0.45(0.21 to 0.69) |
| **Sao Tome and Principe** | 0(0 to 1) | 1.48(0.56 to 3.08) | 1(0 to 2) | 1.50(0.57 to 3.47) | -0.06(-0.72 to 0.61) | 54(23 to 103) | 213.32(90.18 to 409.49) | 123(49 to 242) | 221.16(86.34 to 437.20) | -0.17(-1.38 to 1.06) |
| **Saudi Arabia** | 118(54 to 228) | 3.78(1.73 to 7.31) | 282(136 to 512) | 2.64(1.26 to 4.80) | -1.15(-1.42 to -0.87) | 12408(6020 to 21836) | 391.35(187.95 to 689.83) | 35657(15538 to 66183) | 336.09(146.37 to 623.21) | -0.42(-0.70 to -0.13) |
| **Senegal** | 17(7 to 33) | 1.04(0.46 to 2.05) | 46(20 to 87) | 1.33(0.56 to 2.56) | 0.74(0.06 to 1.43) | 2454(1080 to 5029) | 151.50(65.26 to 316.51) | 6092(2926 to 10896) | 168.23(80.32 to 300.29) | 0.31(-0.35 to 0.97) |
| **Serbia** | 25(13 to 43) | 1.05(0.55 to 1.82) | 8(4 to 13) | 0.36(0.19 to 0.63) | -4.23(-4.53 to -3.92) | 3126(1444 to 5706) | 132.45(61.41 to 241.03) | 1921(600 to 4470) | 89.84(29.14 to 206.99) | -1.70(-2.19 to -1.21) |
| **Seychelles** | 0(0 to 1) | 1.64(0.81 to 2.88) | 0(0 to 1) | 1.25(0.68 to 2.11) | -0.60(-0.95 to -0.25) | 28(15 to 48) | 155.25(81.22 to 262.14) | 33(17 to 58) | 132.99(67.27 to 235.40) | -0.26(-0.70 to 0.17) |
| **Sierra Leone** | 12(5 to 25) | 1.24(0.54 to 2.70) | 107(46 to 199) | 5.59(2.36 to 10.40) | 4.90(4.12 to 5.70) | 2390(863 to 4547) | 250.22(86.57 to 480.35) | 10323(5120 to 17485) | 510.33(251.92 to 861.37) | 2.44(1.80 to 3.08) |
| **Singapore** | 4(2 to 6) | 0.37(0.21 to 0.60) | 2(1 to 2) | 0.12(0.07 to 0.19) | -3.65(-4.60 to -2.68) | 1147(276 to 2717) | 119.28(29.13 to 281.73) | 1026(191 to 2666) | 69.90(14.22 to 181.17) | -1.60(-1.85 to -1.35) |
| **Slovakia** | 13(7 to 21) | 0.94(0.51 to 1.59) | 5(3 to 9) | 0.38(0.19 to 0.68) | -2.91(-3.42 to -2.40) | 1830(775 to 3503) | 136.28(57.76 to 260.50) | 1562(392 to 3440) | 117.03(31.12 to 256.25) | -0.54(-0.73 to -0.36) |
| **Slovenia** | 4(2 to 6) | 0.81(0.49 to 1.21) | 1(0 to 1) | 0.14(0.08 to 0.21) | -6.03(-7.04 to -5.00) | 814(359 to 1519) | 161.06(71.50 to 299.47) | 445(129 to 952) | 100.29(30.89 to 213.02) | -1.54(-1.81 to -1.28) |
| **Solomon Islands** | 1(0 to 2) | 1.35(0.45 to 2.83) | 5(3 to 8) | 2.76(1.54 to 4.57) | 2.24(1.74 to 2.74) | 182(84 to 314) | 253.23(116.80 to 433.70) | 555(308 to 903) | 330.02(183.91 to 535.43) | 0.83(0.63 to 1.02) |
| **Somalia** | 40(17 to 80) | 2.34(0.98 to 4.67) | 323(152 to 632) | 7.23(3.46 to 13.84) | 2.45(0.14 to 4.81) | 5565(2311 to 10200) | 335.02(136.06 to 616.00) | 29351(14165 to 52871) | 641.71(313.02 to 1133.93) | 1.14(-0.36 to 2.67) |
| **South Africa** | 1998(1239 to 2886) | 20.49(12.78 to 29.76) | 2686(1630 to 4079) | 16.94(10.28 to 25.73) | -0.64(-1.47 to 0.21) | 133605(84523 to 189664) | 1349.73(859.07 to 1924.48) | 183806(112461 to 276882) | 1159.33(709.22 to 1747.48) | -0.53(-1.28 to 0.23) |
| **South Sudan** | 38(17 to 83) | 3.13(1.37 to 6.83) | 290(134 to 591) | 13.79(6.32 to 28.27) | 4.95(3.92 to 5.98) | 4521(1980 to 8669) | 366.94(155.44 to 708.44) | 21397(10949 to 39343) | 994.66(508.75 to 1836.32) | 3.30(2.60 to 4.00) |
| **Spain** | 59(41 to 81) | 0.61(0.42 to 0.84) | 30(20 to 41) | 0.29(0.19 to 0.40) | -2.54(-3.98 to -1.08) | 13197(5244 to 26875) | 137.18(54.33 to 279.70) | 16552(3498 to 38453) | 161.75(37.67 to 371.60) | 0.67(-0.03 to 1.37) |
| **Sri Lanka** | 71(36 to 121) | 1.51(0.77 to 2.56) | 28(12 to 55) | 0.50(0.22 to 0.98) | -3.46(-4.16 to -2.75) | 8136(3940 to 13884) | 174.84(84.33 to 298.88) | 4912(1999 to 9383) | 86.66(35.59 to 165.25) | -2.24(-2.85 to -1.63) |
| **Sudan** | 22(10 to 51) | 0.50(0.23 to 1.20) | 469(217 to 1013) | 4.60(2.11 to 9.98) | 7.08(1.13 to 13.38) | 11089(3062 to 23920) | 251.91(66.92 to 542.85) | 52670(23734 to 95494) | 496.70(223.64 to 896.36) | 1.29(0.70 to 1.88) |
| **Suriname** | 5(2 to 7) | 4.68(2.50 to 7.52) | 6(3 to 10) | 4.32(2.29 to 7.16) | -0.07(-0.59 to 0.46) | 408(202 to 697) | 417.16(207.06 to 715.24) | 644(301 to 1199) | 444.01(208.36 to 825.81) | 0.29(-0.36 to 0.95) |
| **Sweden** | 16(10 to 23) | 0.76(0.46 to 1.11) | 9(5 to 12) | 0.39(0.23 to 0.56) | -2.37(-3.00 to -1.73) | 3990(1135 to 9110) | 190.90(56.07 to 432.89) | 5701(854 to 12924) | 252.08(40.11 to 571.57) | 0.96(0.59 to 1.34) |
| **Switzerland** | 22(18 to 27) | 1.22(0.98 to 1.52) | 6(4 to 9) | 0.32(0.20 to 0.44) | -4.55(-5.58 to -3.51) | 3025(1659 to 5598) | 168.97(94.45 to 309.12) | 2418(736 to 5856) | 118.52(38.45 to 282.97) | -1.13(-1.68 to -0.58) |
| **Syrian Arab Republic** | 20(11 to 34) | 0.75(0.40 to 1.24) | 52(31 to 82) | 1.45(0.86 to 2.23) | 3.62(1.10 to 6.20) | 5608(2215 to 11584) | 217.36(81.36 to 456.44) | 10117(3866 to 20738) | 275.49(106.56 to 565.06) | 1.07(0.25 to 1.89) |
| **Taiwan (Province of China)** | 60(37 to 87) | 1.07(0.65 to 1.56) | 21(12 to 33) | 0.37(0.21 to 0.56) | -3.51(-4.61 to -2.39) | 10265(6058 to 15742) | 184.16(108.08 to 283.78) | 8317(4218 to 14418) | 145.58(74.78 to 249.57) | -0.77(-1.06 to -0.49) |
| **Tajikistan** | 12(7 to 20) | 1.13(0.63 to 1.82) | 10(6 to 16) | 0.39(0.22 to 0.63) | -2.95(-4.81 to -1.05) | 1444(708 to 2558) | 127.97(63.11 to 225.93) | 2649(751 to 5619) | 104.78(29.32 to 222.58) | -0.73(-1.68 to 0.23) |
| **Thailand** | 511(284 to 853) | 3.19(1.79 to 5.28) | 326(200 to 481) | 1.87(1.14 to 2.79) | -1.69(-2.58 to -0.79) | 42506(23744 to 68378) | 264.14(147.75 to 422.31) | 32017(17320 to 51006) | 187.70(101.68 to 300.01) | -1.18(-1.77 to -0.59) |
| **Timor-Leste** | 2(1 to 4) | 0.92(0.39 to 1.93) | 9(4 to 15) | 2.85(1.52 to 4.84) | 3.86(3.44 to 4.28) | 329(114 to 615) | 173.83(59.08 to 324.65) | 755(396 to 1250) | 238.29(127.22 to 385.44) | 1.01(0.60 to 1.43) |
| **Togo** | 34(16 to 68) | 4.26(1.95 to 8.56) | 110(52 to 201) | 5.38(2.53 to 9.83) | 0.65(-0.08 to 1.39) | 3080(1454 to 5760) | 378.52(176.32 to 718.67) | 9135(4726 to 15189) | 439.11(227.43 to 731.78) | 0.45(-0.03 to 0.94) |
| **Tokelau** | 0(0 to 0) | 1.86(0.69 to 4.24) | 0(0 to 0) | 2.78(1.20 to 5.79) | 1.57(1.14 to 2.00) | 1(0 to 2) | 281.22(131.78 to 503.98) | 1(1 to 2) | 346.06(159.75 to 633.26) | 0.79(0.60 to 0.98) |
| **Tonga** | 1(0 to 1) | 2.73(1.36 to 4.74) | 0(0 to 0) | 0.58(0.30 to 1.04) | -4.90(-5.06 to -4.74) | 63(34 to 102) | 287.70(158.17 to 464.04) | 40(19 to 73) | 160.38(74.12 to 289.74) | -1.88(-2.05 to -1.71) |
| **Trinidad and Tobago** | 15(9 to 22) | 4.77(2.94 to 7.05) | 20(11 to 31) | 5.82(3.34 to 9.03) | 0.61(0.12 to 1.11) | 1245(696 to 1977) | 400.33(222.97 to 637.74) | 1669(913 to 2755) | 493.86(269.53 to 813.50) | 0.60(-0.61 to 1.82) |
| **Tunisia** | 6(3 to 12) | 0.32(0.15 to 0.60) | 8(3 to 14) | 0.24(0.11 to 0.44) | -1.62(-4.88 to 1.75) | 4270(1222 to 9726) | 220.17(59.73 to 502.90) | 8335(1538 to 21302) | 260.96(49.89 to 667.96) | 0.42(0.08 to 0.76) |
| **Turkey** | 138(65 to 254) | 0.99(0.47 to 1.81) | 143(81 to 230) | 0.66(0.37 to 1.06) | -1.21(-1.87 to -0.54) | 41662(13743 to 78369) | 299.83(96.33 to 564.45) | 62122(17729 to 129572) | 283.28(82.46 to 589.48) | -0.15(-0.48 to 0.18) |
| **Turkmenistan** | 10(6 to 16) | 1.19(0.71 to 1.87) | 9(5 to 14) | 0.68(0.39 to 1.12) | -1.74(-3.11 to -0.36) | 1031(537 to 1811) | 120.11(62.30 to 213.35) | 1152(495 to 2419) | 91.60(39.32 to 192.40) | -0.79(-1.45 to -0.12) |
| **Tuvalu** | 0(0 to 0) | 3.06(1.29 to 6.05) | 0(0 to 0) | 2.88(1.30 to 5.51) | -0.17(-0.31 to -0.04) | 9(5 to 15) | 357.94(185.19 to 595.85) | 10(5 to 17) | 349.04(175.14 to 595.78) | -0.09(-0.17 to -0.01) |
| **Uganda** | 4002(1607 to 7783) | 126.47(51.15 to 243.05) | 1834(768 to 3364) | 21.29(8.78 to 39.27) | -5.62(-6.19 to -5.05) | 246141(103409 to 466388) | 7429.80(3140.12 to 13927.64) | 147088(76850 to 240584) | 1634.24(848.23 to 2681.09) | -4.83(-5.36 to -4.29) |
| **Ukraine** | 187(115 to 289) | 1.44(0.88 to 2.25) | 163(97 to 253) | 1.40(0.82 to 2.21) | -0.07(-0.87 to 0.74) | 22557(10377 to 41813) | 172.98(80.13 to 319.60) | 17505(8292 to 34011) | 154.35(73.28 to 301.12) | -0.36(-0.90 to 0.18) |
| **United Arab Emirates** | 1(0 to 2) | 0.31(0.14 to 0.59) | 3(1 to 6) | 0.21(0.09 to 0.39) | -1.23(-2.08 to -0.36) | 664(201 to 1437) | 195.83(57.25 to 428.72) | 3588(815 to 8565) | 192.10(48.22 to 456.02) | 0.00(-0.20 to 0.21) |
| **United Kingdom** | 70(45 to 98) | 0.49(0.31 to 0.69) | 29(20 to 39) | 0.18(0.12 to 0.25) | -3.03(-4.55 to -1.49) | 26775(6184 to 59271) | 186.05(44.10 to 410.96) | 33921(4158 to 77016) | 213.94(28.11 to 487.52) | 0.67(0.23 to 1.10) |
| **United Republic of Tanzania** | 1555(647 to 2998) | 30.54(12.59 to 58.89) | 2126(963 to 3874) | 16.31(7.33 to 29.79) | -2.04(-3.04 to -1.03) | 104449(49208 to 189396) | 1959.62(915.97 to 3553.55) | 162895(85377 to 270161) | 1208.19(630.70 to 2003.64) | -1.59(-2.47 to -0.70) |
| **United States of America** | 2002(1331 to 2870) | 2.94(1.93 to 4.24) | 1205(762 to 1791) | 1.58(0.99 to 2.36) | -2.03(-2.42 to -1.63) | 216496(108849 to 347237) | 319.26(160.31 to 513.09) | 231236(71958 to 478660) | 306.53(95.73 to 633.99) | -0.03(-0.36 to 0.29) |
| **United States Virgin Islands** | 2(1 to 4) | 7.57(3.96 to 13.03) | 1(0 to 1) | 4.65(2.20 to 8.88) | -0.78(-1.30 to -0.25) | 156(84 to 260) | 554.32(297.83 to 926.19) | 66(31 to 119) | 397.49(186.43 to 711.77) | -0.56(-0.99 to -0.13) |
| **Uruguay** | 9(6 to 15) | 1.25(0.74 to 2.01) | 11(7 to 17) | 1.31(0.80 to 2.02) | 0.20(-0.89 to 1.30) | 1204(529 to 2333) | 160.44(70.55 to 310.94) | 1720(639 to 3734) | 205.06(76.61 to 443.36) | 0.76(0.30 to 1.23) |
| **Uzbekistan** | 51(30 to 81) | 1.13(0.68 to 1.77) | 36(21 to 57) | 0.39(0.23 to 0.63) | -3.39(-4.34 to -2.43) | 5393(2825 to 9685) | 116.53(60.72 to 210.73) | 6823(2533 to 15910) | 74.83(27.89 to 173.96) | -1.41(-1.88 to -0.94) |
| **Vanuatu** | 0(0 to 0) | 0.25(0.11 to 0.47) | 1(0 to 2) | 1.14(0.55 to 2.10) | 4.93(4.08 to 5.78) | 62(24 to 114) | 176.74(67.02 to 327.77) | 176(84 to 306) | 227.89(109.54 to 395.94) | 0.81(0.70 to 0.93) |
| **Venezuela (Bolivarian Republic of)** | 40(24 to 63) | 0.82(0.50 to 1.27) | 126(73 to 205) | 1.87(1.07 to 3.08) | 2.92(1.42 to 4.45) | 6152(2107 to 13553) | 130.94(42.56 to 291.68) | 14166(5977 to 28171) | 206.96(89.43 to 408.07) | 1.51(0.69 to 2.34) |
| **Viet Nam** | 66(29 to 132) | 0.38(0.17 to 0.75) | 110(59 to 194) | 0.42(0.22 to 0.74) | 0.52(0.08 to 0.96) | 15407(6143 to 29272) | 90.39(34.92 to 172.02) | 27538(9359 to 53962) | 105.52(36.26 to 207.72) | 0.48(0.35 to 0.61) |
| **Yemen** | 19(7 to 40) | 0.78(0.31 to 1.57) | 81(38 to 169) | 1.01(0.48 to 2.09) | 1.35(-1.41 to 4.18) | 6657(2128 to 14630) | 258.98(81.24 to 573.06) | 23159(8325 to 49432) | 288.54(102.16 to 618.45) | 0.37(-0.26 to 1.01) |
| **Zambia** | 1038(434 to 2055) | 64.61(26.47 to 130.18) | 1405(589 to 2702) | 34.68(14.34 to 67.57) | -2.06(-2.84 to -1.28) | 69395(31706 to 128668) | 4115.43(1854.45 to 7755.56) | 93361(45271 to 165692) | 2220.83(1064.56 to 3997.77) | -2.07(-2.80 to -1.33) |
| **Zimbabwe** | 1223(491 to 2499) | 57.82(23.01 to 119.07) | 1159(513 to 2195) | 32.48(14.16 to 62.21) | -1.84(-2.47 to -1.20) | 81075(35595 to 157984) | 3658.94(1595.77 to 7182.52) | 74691(37520 to 130992) | 2037.44(1010.59 to 3613.37) | -1.89(-2.38 to -1.40) |

**Notes:** Rates are reported per 100,000 person-years. Data in parentheses are 95% uncertainty intervals for cases and age-standardized rates of mortality and DALYs, and 95% confidence intervals for AAPCs. **Abbreviations:** DALYs, disability-adjusted life-years; ASMR, age-standardized mortality rate; ASDR, age-standardized DALYs rate; AAPC, average annual percent change; UI, uncertainty interval; CI, confidence interval.

**
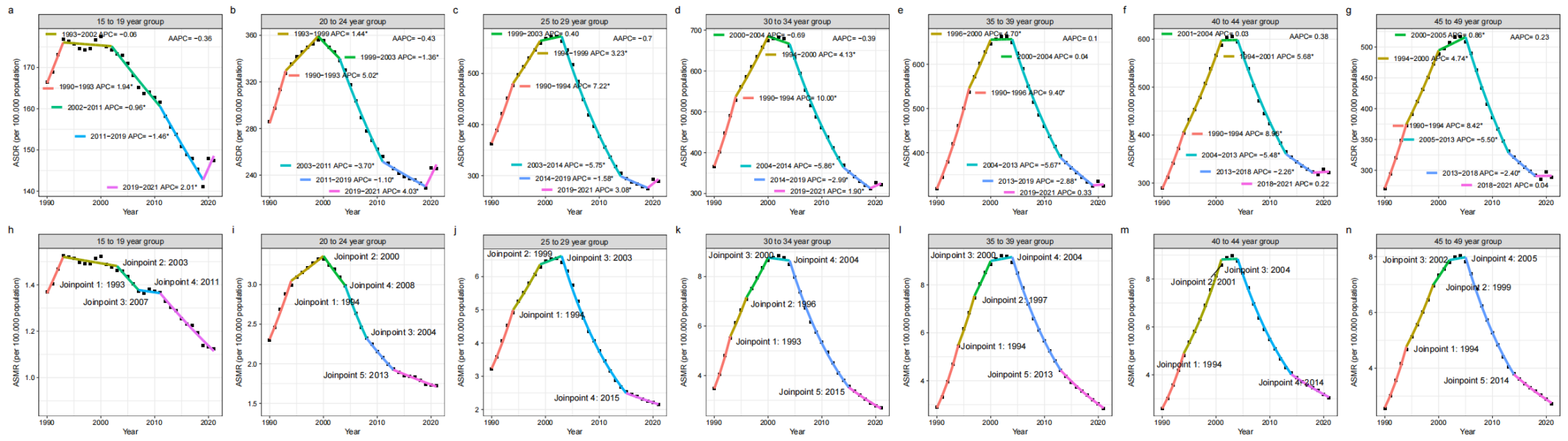
**

**Figure S1. Joinpoint regression analysis** **in** **age-specific** **mortality rate (a-g) and age-specific DALY rate (h-n) of** **global disease burden** **attributable to** **IPV among women of childbearing age from 1990 to 2021.**

**Abbreviations:** AAPC, average annual percent change; APC, annual percentage change; DALYs, disability-adjusted life-years; ASDR, age-specific DALYs rate; ASMR, age-standardized mortality rate; IPV, Intimate Partner Violence.


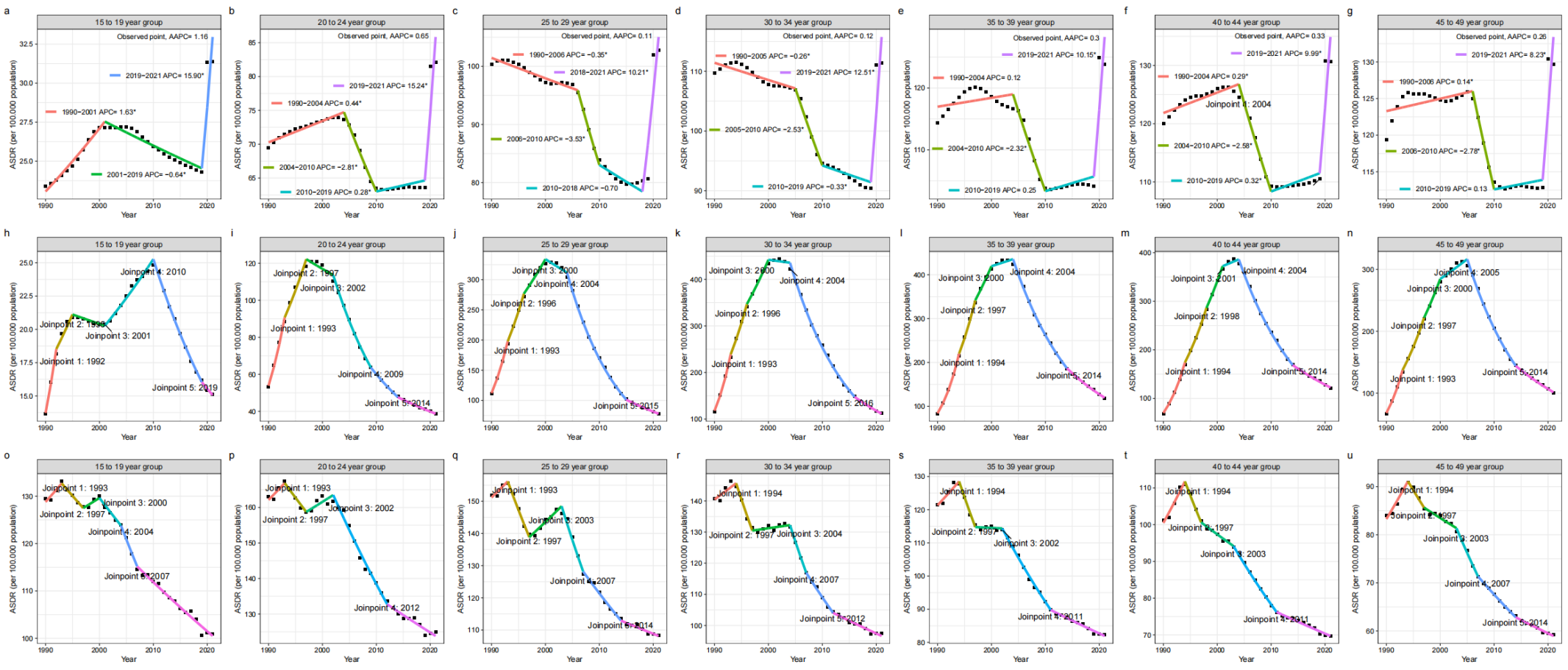


**Figure S2. Joinpoint regression analysis in age-specific DALY rate of global depressive disorders (a-g),** **HIV/AIDS (h-n), and** **interpersonal violence (o-u) burden attributable to IPV among women of childbearing age from 1990 to 2021.**

**Abbreviations:** AAPC, average annual percent change; APC, annual percentage change; DALYs, disability-adjusted life-years; ASMR, age-specific DALYs rate; IPV, Intimate Partner Violence.


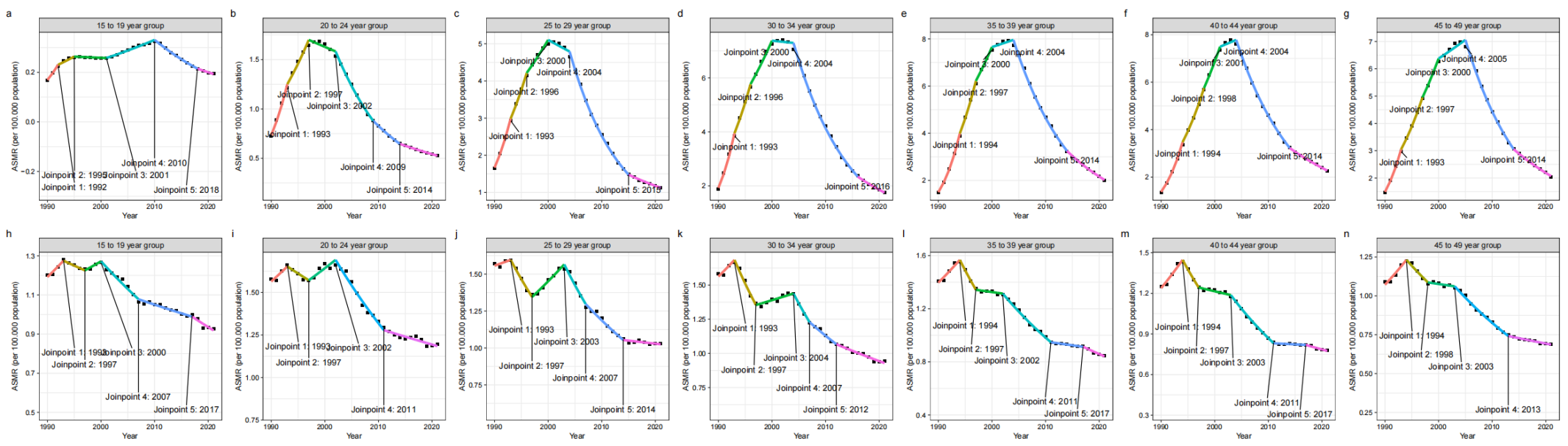


**Figure S3. Joinpoint regression analysis in age-specific mortality rate of global HIV/AIDS (a-g) and interpersonal violence (h-n) burden attributable to IPV among women of childbearing age from 1990 to 2021.**

**Abbreviations:** AAPC, average annual percent change; APC, annual percentage change; DALYs, disability-adjusted life-years; ASDR, age-specific mortality rate; IPV, Intimate Partner Violence.


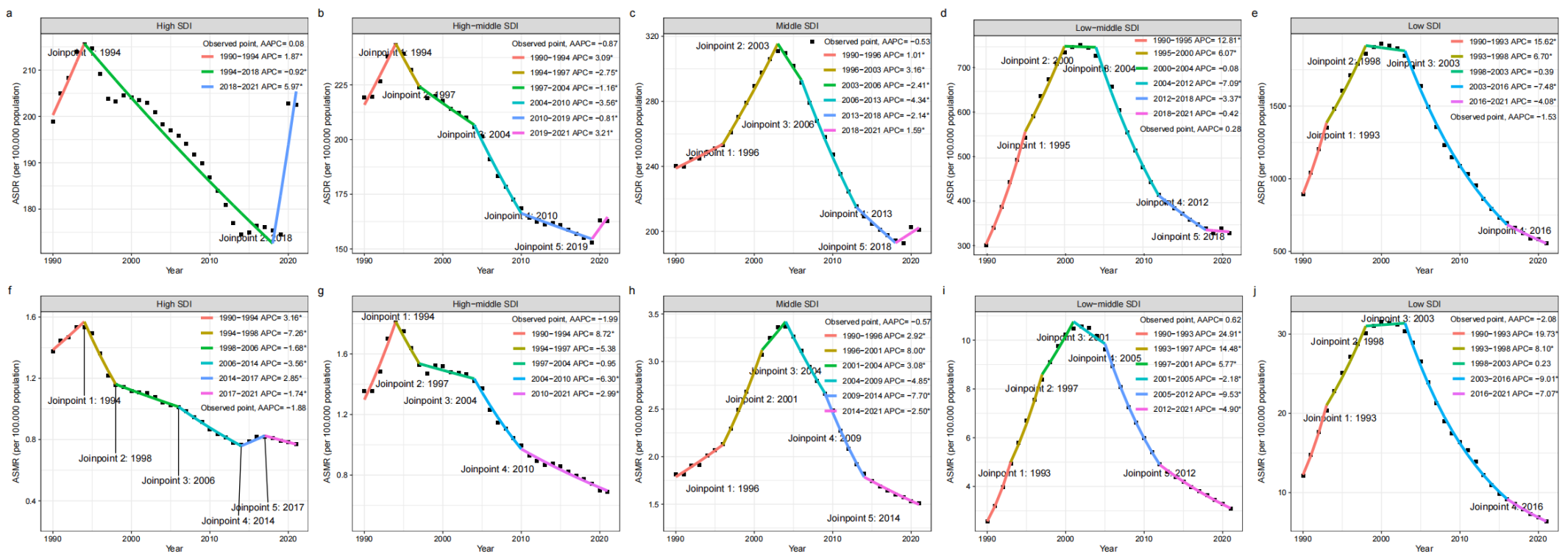


**Figure S4. Joinpoint regression analysis of the disease burden attributable to IPV among women of childbearing age in 5 SDI regions from 1990 to 2021 in terms of ASDR (a-e) and ASMR (f-j).**

**Abbreviations:** AAPC, average annual percent change; APC, annual percentage change; DALYs, disability-adjusted life-years; ASMR, age-standardized mortality rate; ASDR, age-standardized DALYs rate; IPV, Intimate Partner Violence.


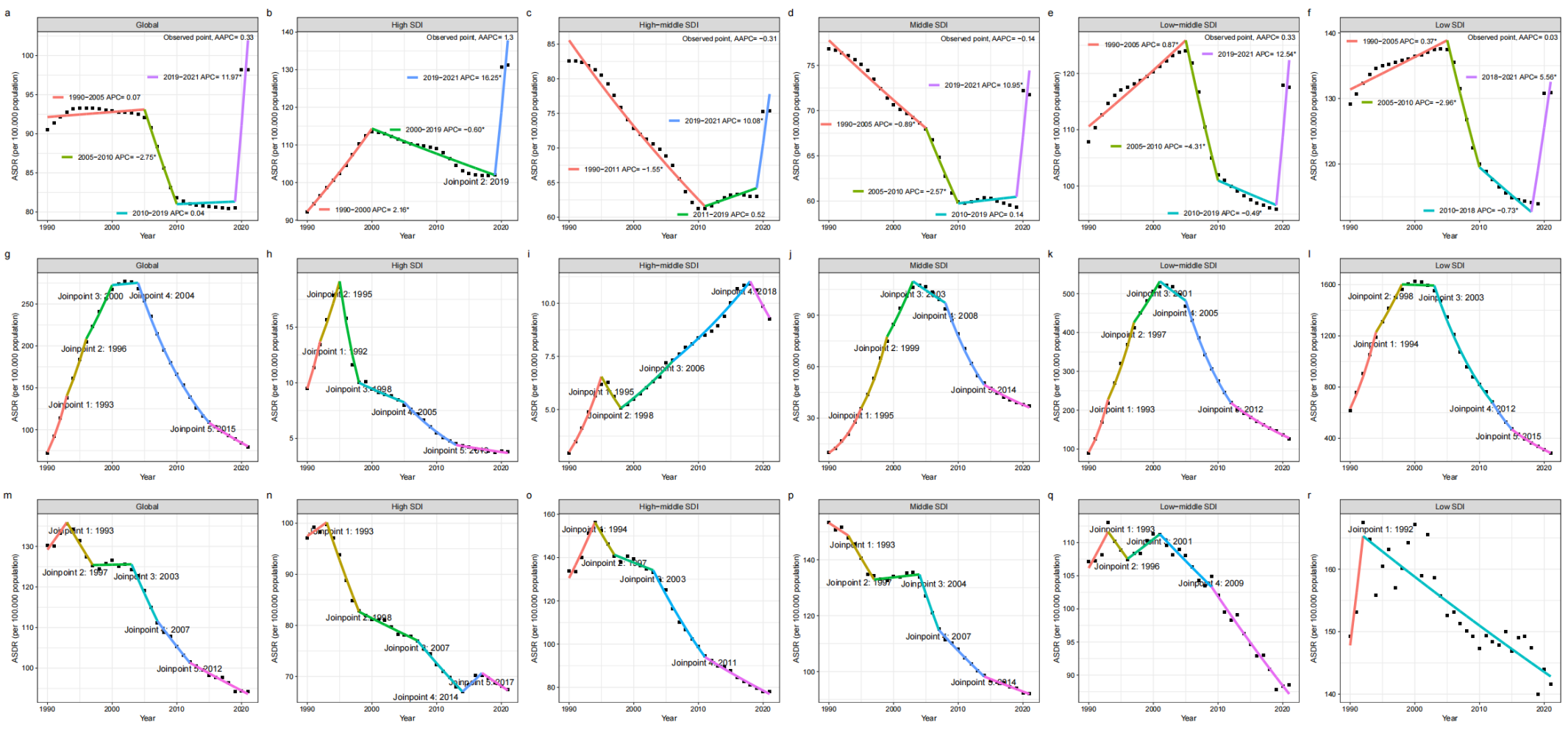


**Figure S5. Joinpoint regression analysis of** **the depressive disorders (a-f), HIV/AIDS (g-l), and interpersonal violence (m-f) burden attributable to IPV among women of childbearing age in global and 5 SDI regions from 1990 to 2021 in terms of ASDR.**

**Abbreviations:** AAPC, average annual percent change; APC, annual percentage change; DALYs, disability-adjusted life-years; ASDR, age-standardized DALYs rate; IPV, Intimate Partner Violence.


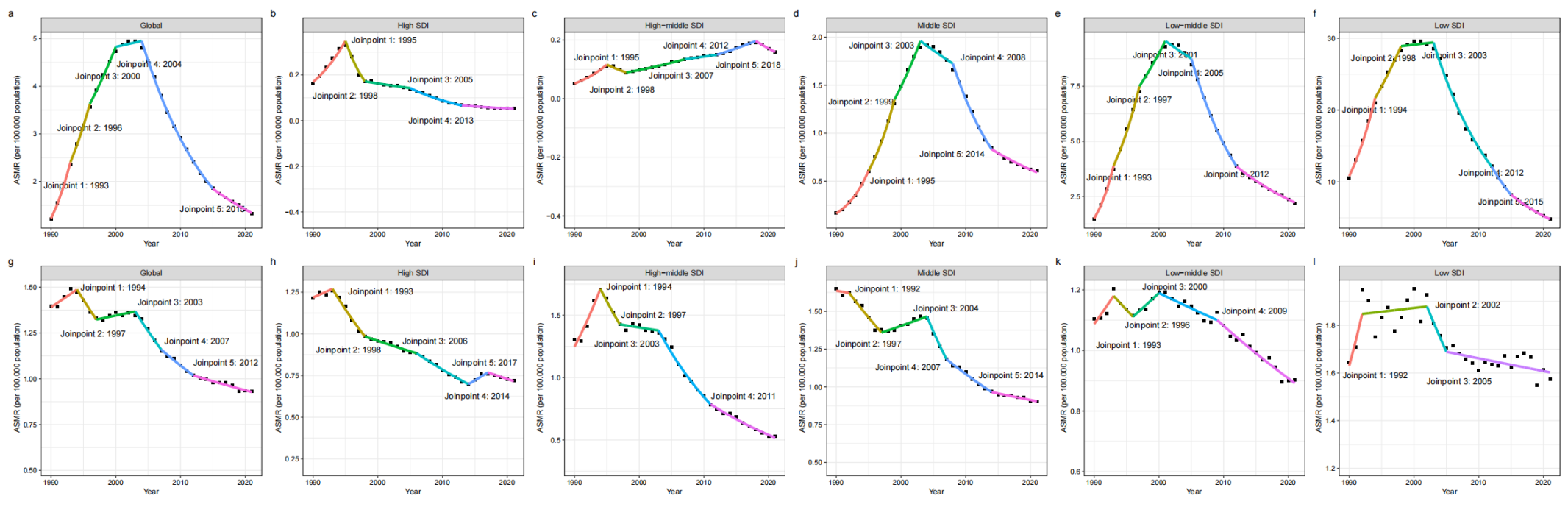


**Figure S6. Joinpoint regression analysis of the HIV/AIDS (a-f) and interpersonal violence (g-l)** **burden attributable to IPV among women of childbearing age in global and 5 SDI regions from 1990 to 2021 in terms of ASMR.**

**Abbreviations:** AAPC, average annual percent change; APC, annual percentage change; DALYs, disability-adjusted life-years; ASMR, age-standardized mortality rate; IPV, Intimate Partner Violence.


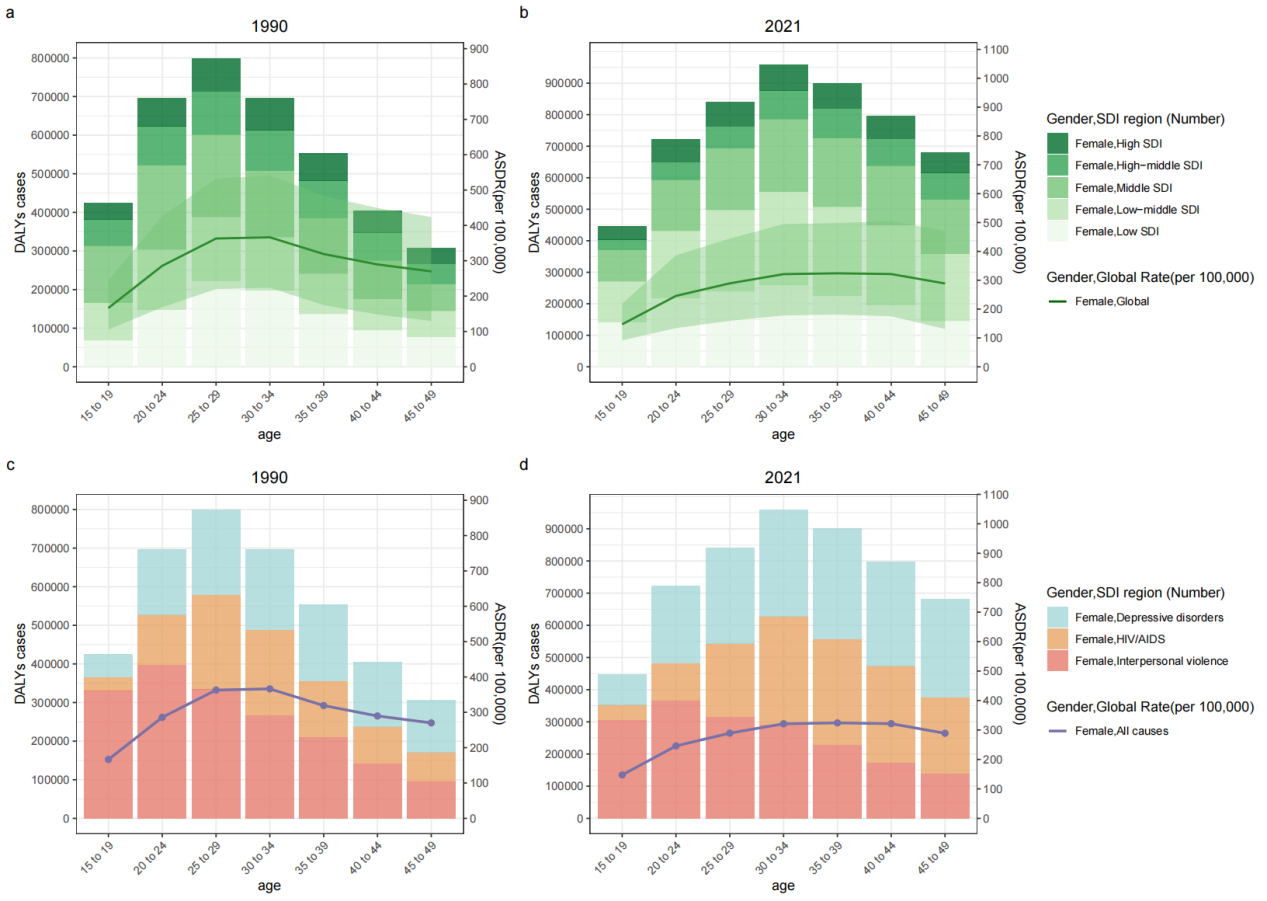


**Figure S7. Burden attributable to IPV in age-specific** **DALYs rates and numbers among women of childbearing age by SDI Regions (a,b) and Cause (c,d) , 1990 vs. 2021.**

**Abbreviations:** DALYs, disability-adjusted life-years; SDI, sociodemographic index; ASDR, age-specific DALYs rate; IPV, Intimate Partner Violence.


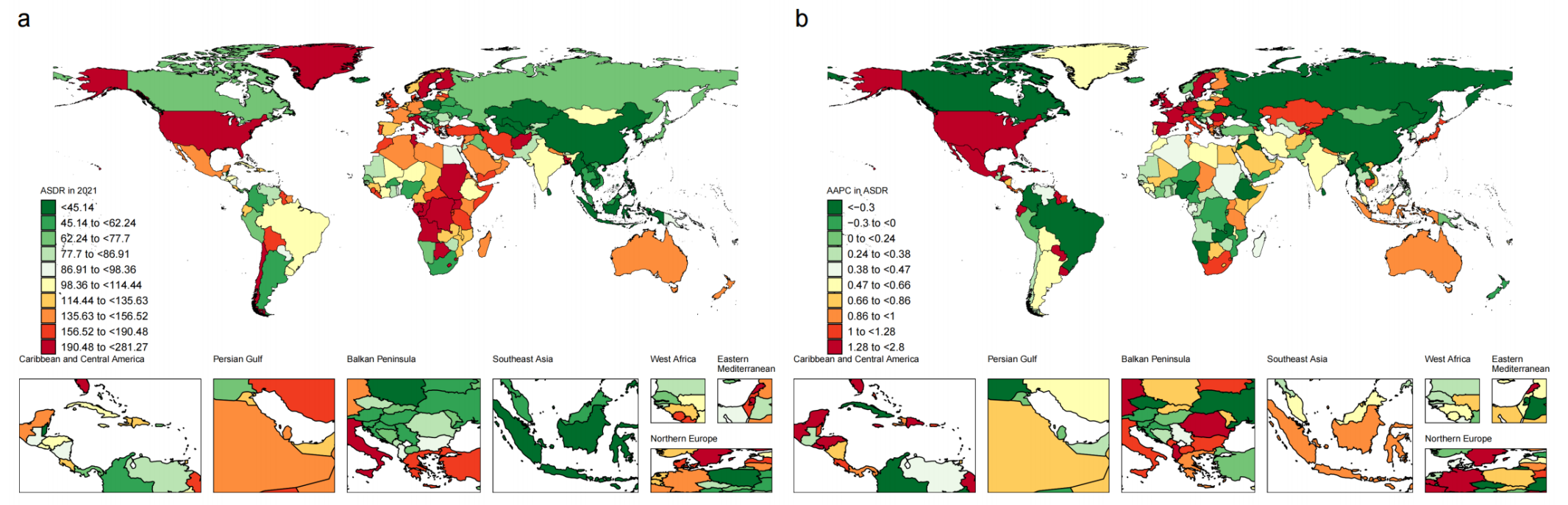


**Figure S8. ASDR in 2021 (a), and their AAPC from 1990 to 2021 (b) for depressive disorders burden attributable to IPV among women of childbearing age, by country**

**Abbreviations:** AAPC, average annual percent change; DALYs, disability-adjusted life-years; ASDR, age-standardized DALYs rate; IPV, Intimate Partner Violence.


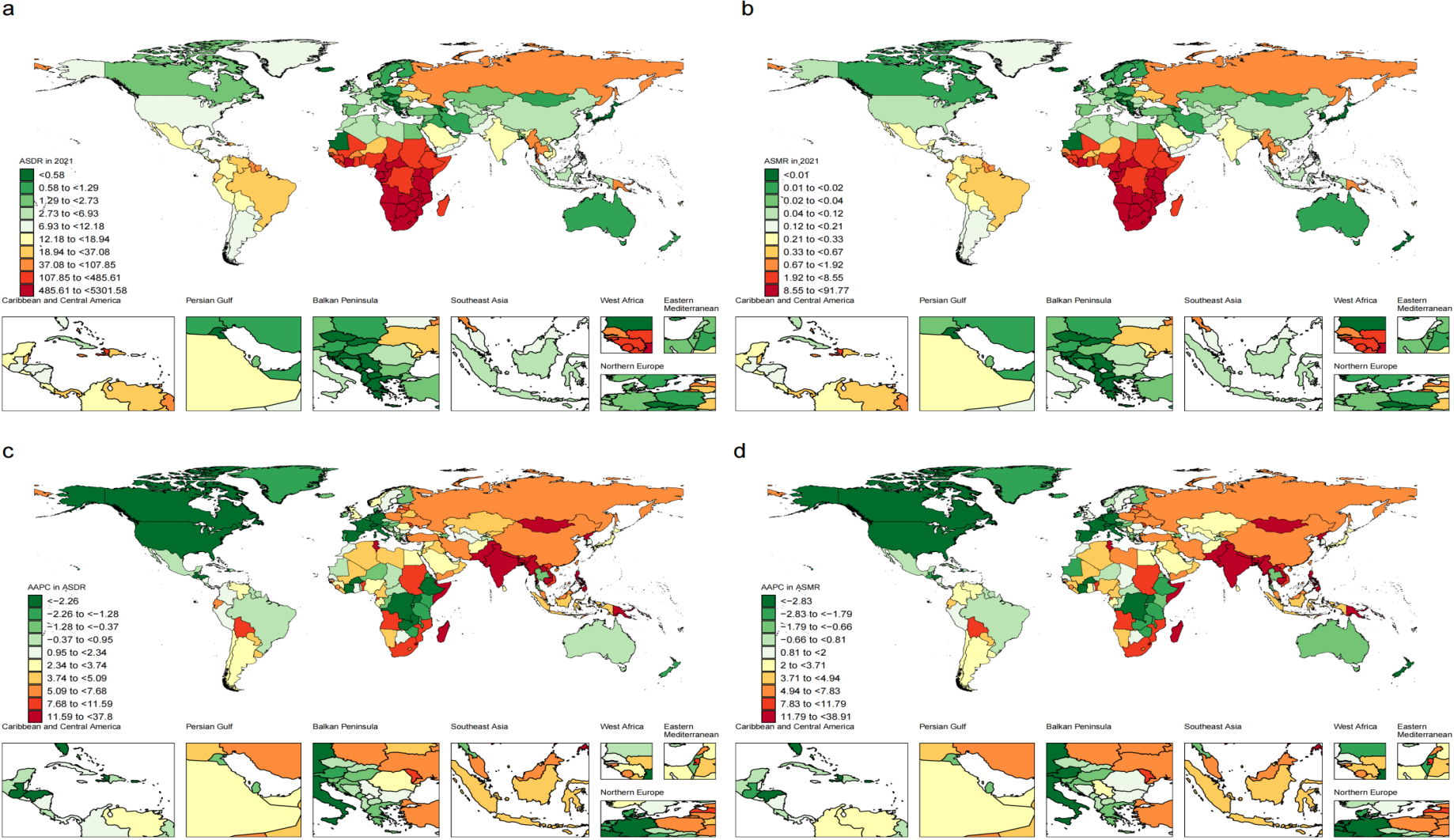


**Figure S9. ASDR and ASMR in 2021 (a-b), and their AAPC from 1990 to 2021 (c-d) for HIV/AIDS burden attributable to IPV among women of childbearing age, by country**

**Abbreviations:** AAPC, average annual percent change; DALYs, disability-adjusted life-years; ASMR, age-standardized mortality rate; ASDR, age-standardized DALYs rate; IPV, Intimate Partner Violence.


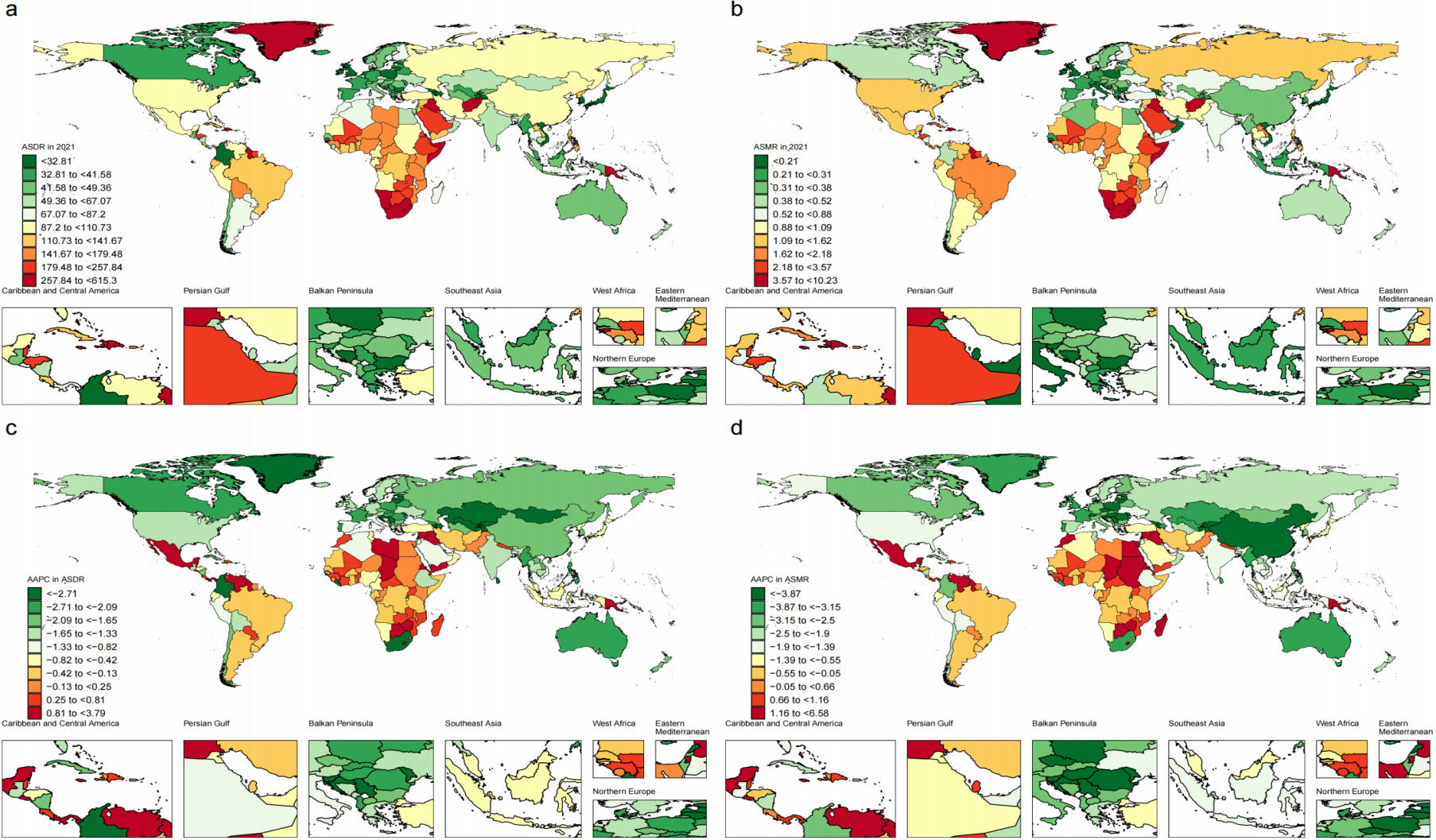


**Figure S10. ASDR and ASMR in 2021 (a-b), and their AAPC from 1990 to 2021 (c-d) for interpersonal violence burden attributable to IPV among women of childbearing age, by country**

**Abbreviations:** AAPC, average annual percent change; DALYs, disability-adjusted life-years; ASMR, age-standardized mortality rate; ASDR, age-standardized DALYs rate; IPV, Intimate Partner Violence.


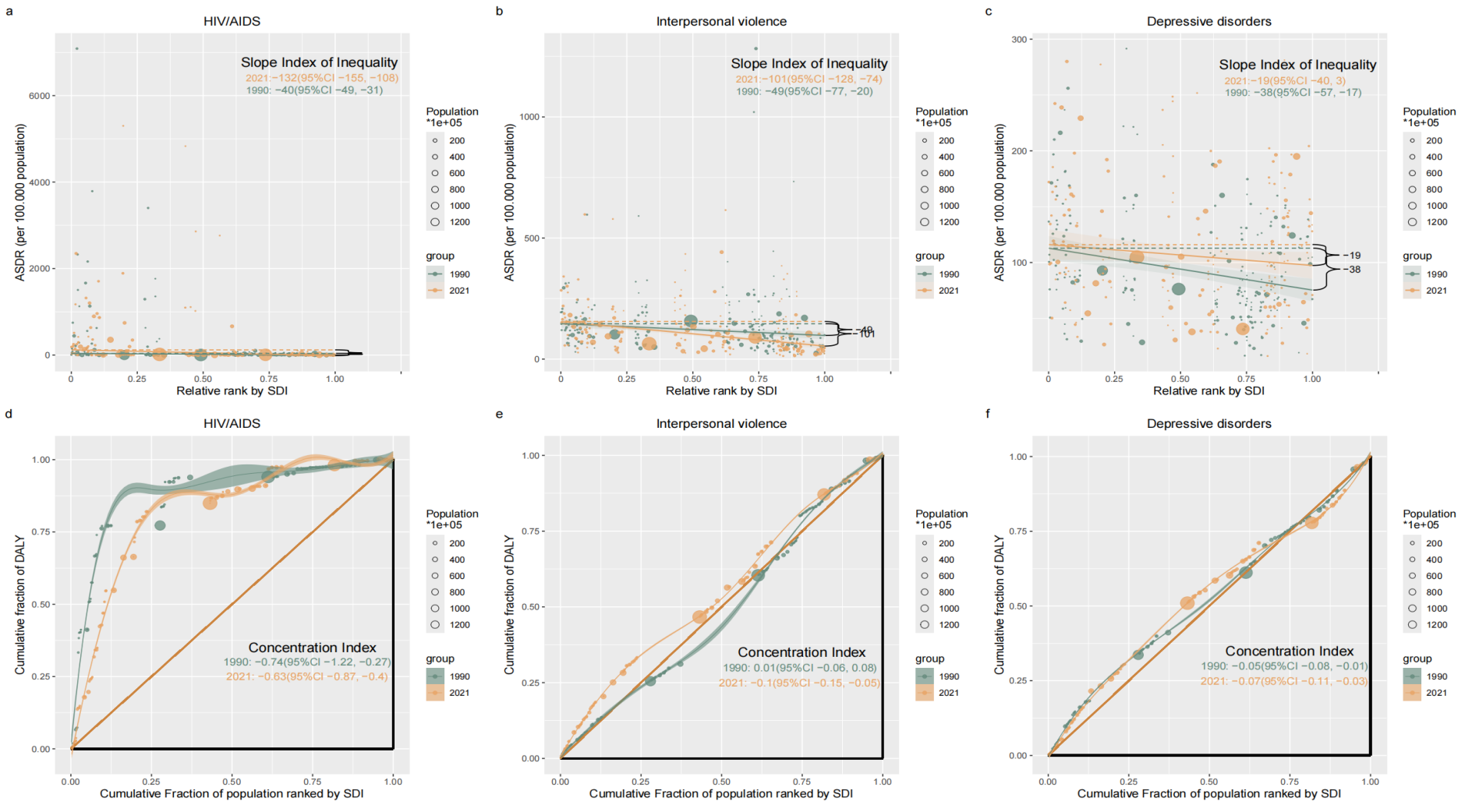


**Figure S11. Absolute healthy inequality (a-c) and relative healthy inequality (d-f) for ASDR of the HIV/AIDS (a,d), interpersonal violence (b,e), and depressive disorders (c,f) burden attributable to IPV among women of childbearing age, 1990 vs. 2021.**

**Abbreviations:** DALYs, disability-adjusted life-years; ASDR, age-standardized DALYs rate; IPV, Intimate Partner Violence; SDI, Socio-demographic index.


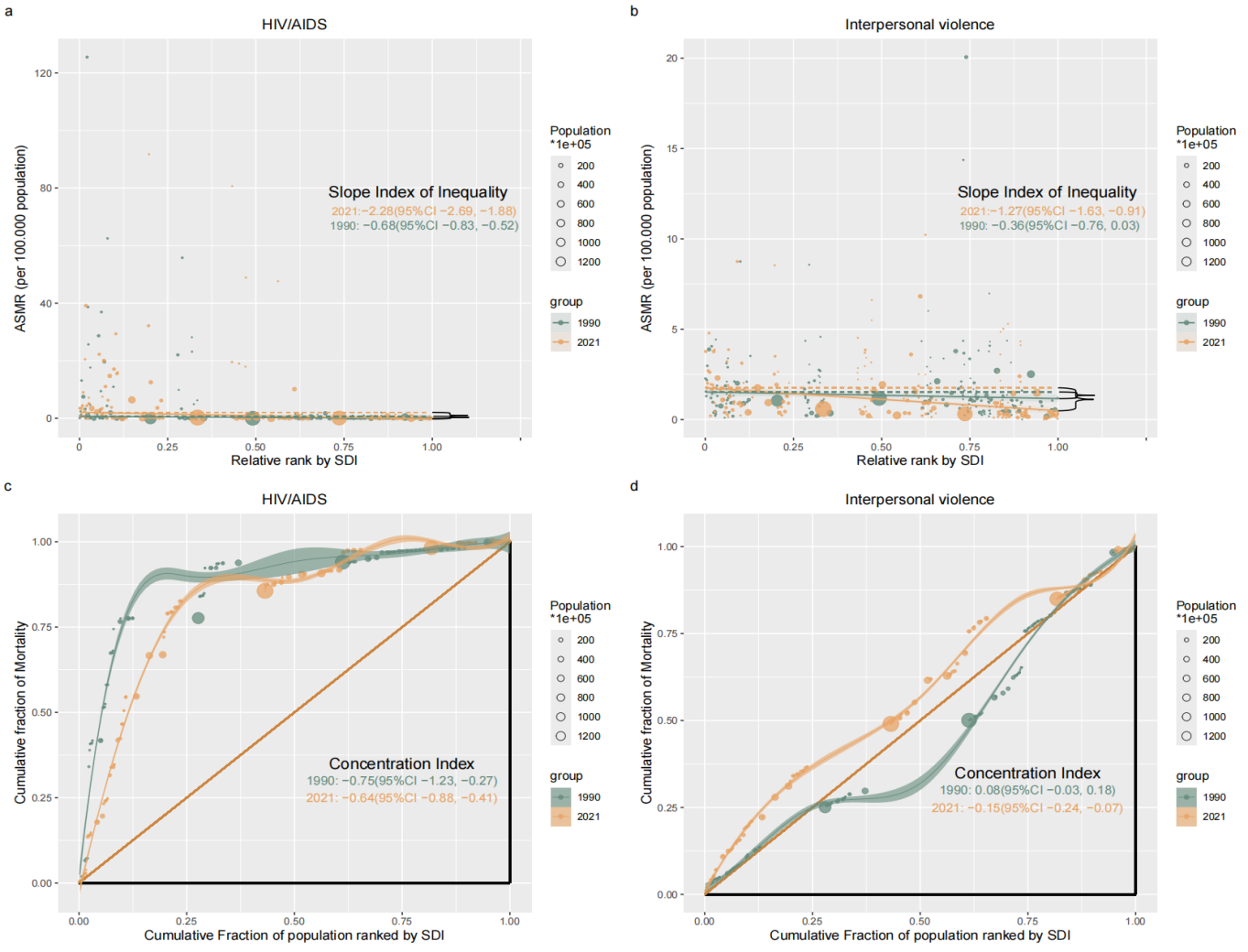


**Figure S12. Absolute healthy inequality (a,b) and relative healthy inequality (c,d) for ASMR of the HIV/AIDS (a,c) and depressive disorders (b,d) burden attributable to IPV among women of childbearing age, 1990 vs. 2021.**

**Abbreviations:** DALYs, disability-adjusted life-years; ASMR, age-standardized mortality rate; IPV, Intimate Partner Violence; SDI, Socio-demographic index.
